# Significant benefits of pollution alerts for cleaner air and better health

**DOI:** 10.1093/pnasnexus/pgag054

**Published:** 2026-03-03

**Authors:** Yuqing Dai, Juncheng Qian, Yue Yang, Bowen Liu, Shuyu Li, Kun Zhang, Qiaorong Xie, Chengxu Tong, Ying Chen, Angus Robert MacKenzie, Zongbo Shi

**Affiliations:** School of Geography, Earth and Environmental Science, University of Birmingham, Birmingham B15 2TT, United Kingdom; School of Geography, Earth and Environmental Science, University of Birmingham, Birmingham B15 2TT, United Kingdom; China Metallurgical Industry Planning and Research Institute, Beijing 10013, China; Department of Management, Business School, University of Birmingham, Birmingham B15 2TT, United Kingdom; Department of Economics, Business School, University of Birmingham, Birmingham B15 2TT, United Kingdom; School of Environmental Science and Engineering, Southern University of Science and Technology, Shenzhen 518055, China; Department of Chemistry, Purdue University, West Lafayette, IN 47907, USA; Institute of Surface-Earth System Science, School of Earth System Science, Tianjin University, Tianjin 300072, China; School of Geography, Earth and Environmental Science, University of Birmingham, Birmingham B15 2TT, United Kingdom; School of Geography, Earth and Environmental Science, University of Birmingham, Birmingham B15 2TT, United Kingdom; School of Geography, Earth and Environmental Science, University of Birmingham, Birmingham B15 2TT, United Kingdom; School of Geography, Earth and Environmental Science, University of Birmingham, Birmingham B15 2TT, United Kingdom

**Keywords:** air quality, air pollution alert, short-term interventions, gaseous pollutants, particulate matter

## Abstract

While air quality has improved in many cities, short-term spikes in urban pollution continue to cause elevated health risks. To mitigate such risks, pollution alerts trigger short-term interventions (e.g. temporary industrial curtailments or shutdowns, on-road traffic restrictions, construction bans with dust control, and public health advisories) to rapidly cut emissions and exposure. However, the effectiveness of such alerts has remained uncertain. Here, we analyzed air quality and weather data from 57 cities across northern China between 2018 and 2022 and used a two-step machine learning chain to predict counterfactual concentrations under a no-alert (no-intervention) scenario. Our findings show that interventions enacted under alerts effectively reduced pollutant concentrations, with particulate matter (PM) decreasing by 20–40% and nitrogen dioxide (NO_2_) by 5–25% across different cities, reflecting the variability in alert effectiveness among locations. The reduction in PM_2.5_ is estimated to have prevented nearly 54,000 ± 6,000 (∼11%) premature deaths during the study period. Over 80% of these avoided deaths occurred in regions characterized by heavy industries, high coal consumption, and dense population (e.g. Henan, Hebei, and Shandong), where alert-driven interventions had greater impacts. In contrast, service-oriented cities such as Beijing showed moderate but still measurable PM reductions (∼30 μg m^−3^) and correspondingly smaller health benefits. These results provide the first multi-year, multi-city evidence that pollution alerts—through the interventions they trigger—deliver significant and repeatable air quality and public health benefits, offering actionable support for short-term response protocols that complement long-term emission controls in cities worldwide.

Significance Statement Frequent winter haze led China to adopt a multitiered alert system to mitigate acute health risks. In practice, alerts activate enforceable, short-term intervention measures (e.g. curtailments or temporary shutdowns, construction suspensions with dust control, restrictions on heavy-duty diesel vehicles, traffic controls, and public-health advisories) drawn from contingency plans. These measures vary across cities mainly in their stringency, timing, enforcement, and local emission sources. Using weather-controlled counterfactuals for 57 cities (2018–2022), we found that air pollution alert-activated interventions significantly reduced PM_2.5_ (particulate matter), PM_10_, and nitrogen dioxide levels and prevented ∼54,000 ± 6,000 (∼11%) premature deaths. The results provide solid multi-year, multi-city evidence that alert-activated short-term measures yield measurable air quality and health benefits and complement long-term air quality control strategies.

## Introduction

Ambient air pollution remains a leading environmental health risk, responsible for millions of premature deaths worldwide each year (1). Exposure to fine particles (particulate matter, PM_2.5_) in particular contributes to elevated risks of cardiovascular and respiratory diseases, particularly among vulnerable populations (2). Pollution alerts (and similar early-warning systems) have been widely adopted as emergency measures for public health in many countries (3, 4). When air quality is forecast to deteriorate beyond set limits, authorities or service providers issue alerts that either trigger short-term mitigation actions or simply inform the public of the impending pollution event depending on enforcement capacities (3, 5). China's color-coded alert system provides a useful example. City authorities issue tiered pollution alerts (yellow, orange, and red) when forecasts predict high Air Quality Index (i.e. mainly due to PM_2.5_). In practice, an alert activates a package of short-term interventions, for example, traffic restrictions, temporary factory curtailments or shutdowns, construction activity bans with dust suppression, and public health advisories, intended to rapidly cut emissions during the high-pollution event (6, 7); these short-term interventions seek to reduce peak pollutant concentrations and thereby lessen the immediate health impacts of extreme haze.

Previous studies have evaluated the effectiveness of pollution alerts or similar short-term control campaigns. In China, Beijing's first Red Alert in December 2015 reported a 10–20% short-term reduction in PM_2.5_ and associated health risks on alert days (8, 9). Indian authorities have likewise implemented high-pollution action days, though analyses found the “odd–even” traffic rationing scheme deployed during extreme smog events achieved no significant drop in particle levels (10). An administrative-data study in Sydney, Australia, found that air quality alerts reduced bicycle traffic by 14–35%, indicating immediate behavioral responses to warnings (11). North American cities also issued air quality advisories (e.g. “Spare the Air Days” and “Air Alerts” in the United States) to encourage voluntary emission cuts; some regions have reported increases in public transit use on alert days, but consistent reductions in pollutant levels have not been demonstrated (12). A population-wide regression-discontinuity study in Toronto showed alerts produced a 25% drop in asthma-related emergency department visits but no significant change in cardiovascular or respiratory mortality (4). Collectively, these case studies indicate that alert-based interventions can sometimes prompt behavioral changes and air quality benefits. However, the magnitude and consistency of those benefits remain uncertain across different settings and alert designs, which naturally raises several important knowledge gaps regarding the overall efficacy of pollution alert system.

First, prior studies focused on single cities and the immediate aftermath (e.g. hours or days) of an alert, lacking a long-term perspective and leaving it unclear whether pollution drops observed in one event persist across years or repeated alerts. Second, results from one city may not generalize to others, as there is substantial spatial heterogeneity in emissions and meteorology across different cities, yet no study has compared alert impacts across regions. Compounding these issues, meteorological variability, seasonal emission patterns, and overlapping emission control policies complicate attribution of emission changes to the alerts. A quasi-experimental approach (synthetic control) has been used to isolate the alert effect (13), but there are serious limitations when alerts are widespread and recurrent. It is nearly impossible to find a truly unaffected control group, as air pollution is often at a regional scale, and frequent interventions violate key assumptions of these models. Finally, while short-term pollution reductions are the immediate goal of alerts, the broader health benefits (e.g. illnesses or deaths are averted) across multiple cities and years have not been well quantified.

In this study, we evaluate the effects of pollution alerts on air quality across 57 cities in northern China over a 5-year period from 2018 to 2022 using high-resolution air quality data (14–17), official alert logs, and reanalysis meteorology. The cities are selected as they experience persistently high-pollution levels during the winter heating seasons. Here, we construct counterfactuals at given weather conditions with a two-step machine learning (ML) model chain trained on nonalert days and apply a historical bias correction calibrated on winter high-pollution seasons. The policy effect is defined as observed to quantify pollution reductions attributable to pollution alerts while accounting for seasonal emission surges and ongoing baseline pollution controls in the model. Next, we compare these predicted concentrations to the actual observed levels on alert days to isolate the reductions attributable to alert-triggered measures. Finally, we estimate the public health benefits of these alerts by calculating the number of premature deaths avoided due to alert-triggered PM_2.5_ improvements.

We focus on three questions: (i) what are the long-term trends and seasonal patterns in pollution and alert issuance? (ii) how effective are alerts in lowering concentrations of major pollutants, including PM_2.5_, particles <10 µm in diameter (PM_10_), nitrogen dioxide (NO_2_), and sulfur dioxide (SO_2_)? and (iii) what health benefits (in terms of avoided premature mortality) have resulted from these pollution alerts? Through this comprehensive evaluation, we aim to provide a clearer picture of the alert system's efficacy and its contribution to public health, thereby informing ongoing air quality management and policy optimization in China and beyond. We also discuss practical challenges in implementing pollution alerts and future research directions. Equally important, our evaluation framework provides a replicable and transferable tool for quantifying the impact of pollution response strategies in other areas that still face severe haze events.

## Results

### General air pollution trends and alerts implementation

**Fig. 1.**
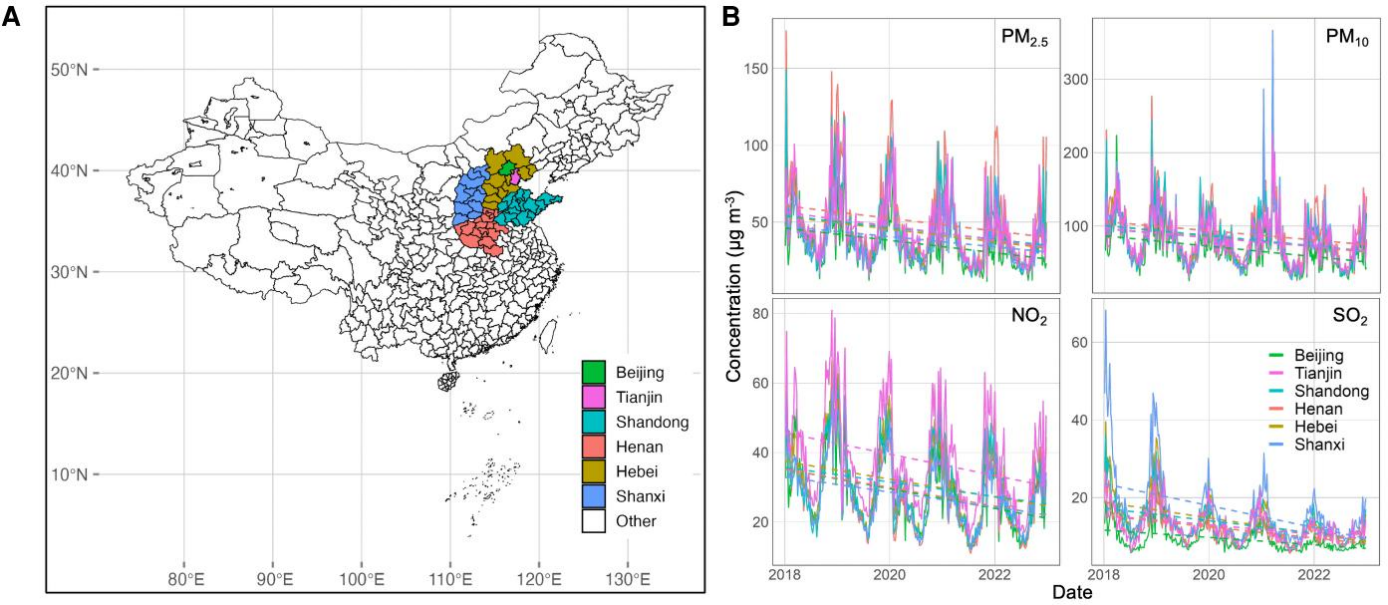
Overview of air pollution trends across northern cities in China. A) Map of China showing the locations of cities that implemented air pollution alerts, with colors classifying key cities by province. B) Weekly trends of air pollutant concentrations by province/municipalities from 2018 to 2023. Dashed lines represent the overall trends of air quality in each province/municipality.

Figure 1A shows the study region on a map of China, highlighting Beijing, Tianjin, and the surrounding provinces of Hebei, Shandong, Henan, and Shanxi. Figure 1B gives weekly average concentrations of PM_2.5_, PM_10_, NO_2_, and SO_2_ (colored by province) over time. Notably, following the promulgation of the Three-Year Action Plan for Winning the Blue-Sky Defence Battle (18), annual average concentrations of PM_2.5_, PM_10_, NO_2_, and SO_2_ across 57 Chinese cities (including six municipalities or provinces) declined substantially between 2018 and 2022. PM_2.5_ decreased by 12.3 μg m^−3^ (a 23.9% reduction, with an interquartile range [IQR] of 5.1%), PM_10_ by 25.5 μg m^−3^ (25.9%, IQR: 4.8%), NO_2_ by 10.8 μg m^−3^ (29.7%, IQR: 6.3%), and SO_2_ by 7.5 μg m^−3^ (38.0%, IQR: 16.9%). These improvements reflect multi-faceted, sustained long-term emission control strategies, including enhanced industrial emission standards, industrial boiler upgrades, the phasing-out of obsolete industrial capacities, the transition to cleaner residential fuels, the closure of small polluting factories, and the enforcement of stricter vehicle emission regulations (19). All four pollutants exhibit strong seasonal variation with persistent wintertime peaks, which are primarily due to suppressed dilution into lower atmospheric boundary layers, unfavorable meteorological conditions for dispersion, and additional pollutant emissions from winter heating (20, 21).

**Fig. 2.**
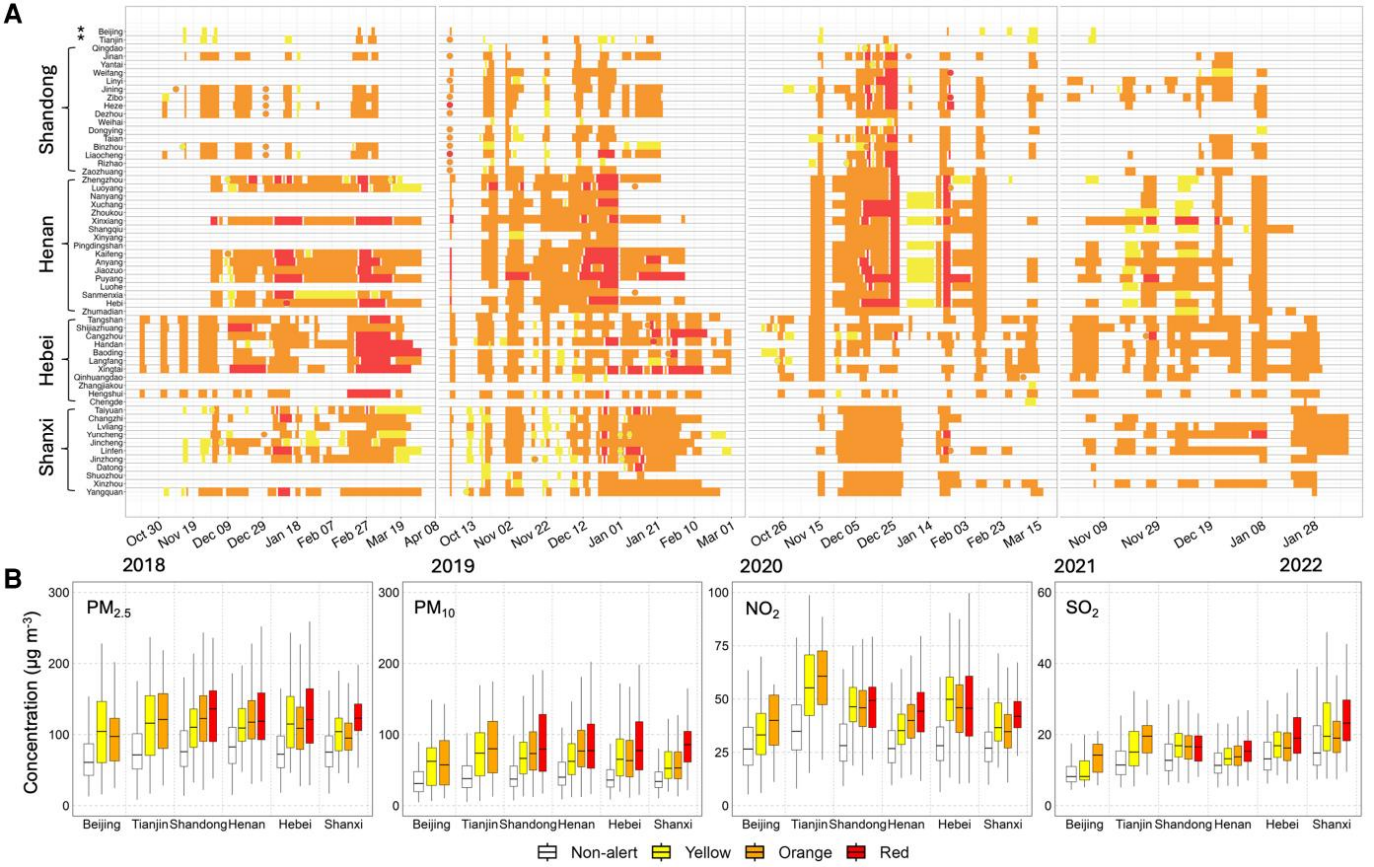
Alert implementation across cities in China and air quality during the alerts. A) Timeline of air pollution alert periods across different cities in China, using color codes to indicate different alert levels. Bar lengths represent the duration of each alert period, while single-day alerts are represented by dots. Absence of a color means no such alert was announced. Cities are ordered by GDP rank as of the year 2021 within each province, with an asterisk (*) marking municipalities under direct central government administration (i.e. Beijing and Tianjin). B) The box plots present observed air pollutant concentrations during yellow, orange, and red alert periods among 2018–2022, with white boxes indicating baseline concentrations on nonalert days for comparison.

Figure 2A visualizes the timing of government-issued yellow, orange, and red air pollution alerts in each city (each row) across different provinces. Alerts were predominantly clustered in late autumn and winter months (heating season), when severe haze events occur most frequently. The frequency and duration of alerts varied by year and location. For instance, early in the study period (winter 2018–2019), numerous cities experienced repeated orange and even red alert days, whereas by winter 2021–2022 far fewer red alerts were needed, corresponding to improved baseline air quality likely due to the COVID-19 pandemic's reduction of road traffic and industrial activity (22). Spatially, there was no clear correlation between a city's gross domestic product (GDP) ranking and the duration of its air pollution alerts. Instead, longer alert periods tended to occur in regions with greater population density (Fig. S1). Cities in industrial provinces like Hebei and Henan experienced the most frequent and prolonged high-level alerts (dense blocks of orange and red in Fig. 2A), whereas Beijing and Tianjin had comparatively fewer red alerts, especially in later years. Zhengzhou (Henan) and Jinan (Shandong), both densely populated transport hubs, also experienced high alert frequencies. These alerts were driven not only by emissions from local industrial and transport sources but also by pollution transport from surrounding regions (23, 24).

### Pollution alerts and their associated interventions improved air quality

Figure 2B illustrates observed pollutant concentrations during alert periods (see Fig. S2 for city-specific details). On average across the cities studied, PM_2.5_ mass concentrations exceeded the World Health Organization (WHO) Air Quality Guideline (AQG) 24-h thresholds (15, 45, 25, and 40 μg m^−3^ for PM_2.5_, PM_10_, NO_2_, and SO_2_, respectively) (25) by a median factor of 5.2 (IQR: 3.6), PM_10_ levels were 2.7 times higher (IQR: 1.3), and NO_2_ exceeded 1.7-fold (IQR: 0.8). In contrast, SO_2_ generally met the limit during pollution alert periods.

**Fig. 3.**
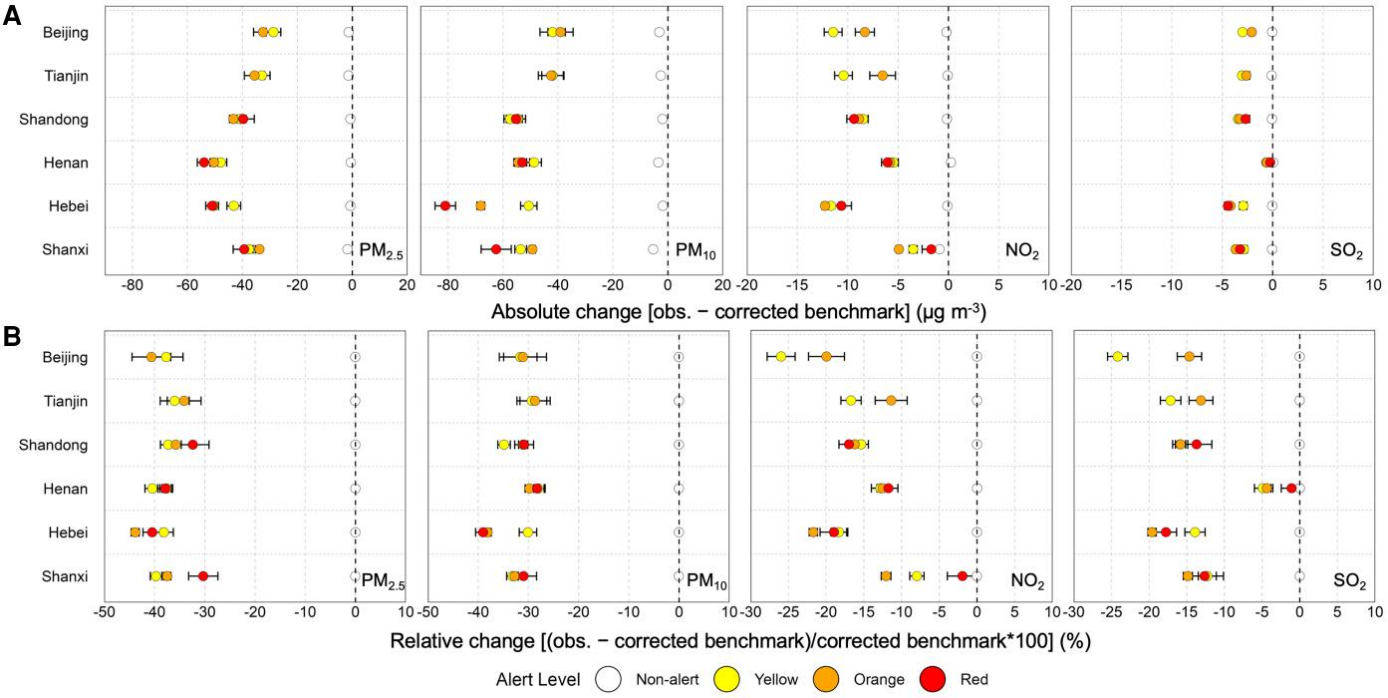
Province-specific pollution anomalies in ground-level air pollution alerts. A) Concentration differences between observed ambient pollutant concentrations (obs.) and corrected predictions from a base model. B) Percentage differences between observed ambient pollutant concentrations (obs.) and corrected predictions from a base model. Points show cross-city mean values for each alert tier (color coded), and error bars represent SEs calculated from variability among city-level estimates. White symbols denote corresponding anomalies on nonalert days, as a control to illustrate baseline model–observation agreement in the absence of alerts.

Figure 3A shows the estimated reduction in concentrations (difference between observed and model-predicted baseline, in μg m^−3^) for four pollutants during alert days in each province, and Fig. 3B provides the percentages (%) or the reductions. During alert days, observed pollutant concentrations of PM_2.5_, PM_10_, NO_2_, and SO_2_ were consistently lower than model-predicted levels absent alerts, indicating that the alert-related emergency interventions have translated into measurable air quality improvements (city-specific details in Fig. S4). On average, daily PM_2.5_ and PM_10_ levels dropped by ∼44.3 ± 1.5 μg m^−3^ (hereafter reported as mean ± SE for pollutant variations) and 53.5 ± 1.6 μg m^−3^ during alerts, corresponding to ∼38 and 33% reductions from contrafactual business as usual (BAU) scenarios, respectively. Most reductions in PM were observed in cities (provinces) with heavy industries. For instance, Hebei experienced one of the largest PM_2.5_ drops (on the order of 49.4 ± 0.5 μg m^−3^, or ∼43%), with similar strong reductions in neighboring Henan (50.1 ± 0.5 μg m^−3^, ∼38%) and Shandong (41.9 ± 0.6 μg m^−3^, ∼36%). In contrast, Beijing and Tianjin showed relatively moderate but still meaningful PM concentration declines (29.8 ± 2.1 and 34.0 ± 2.3 μg m^−3^, respectively). NO_2_ concentrations also decreased substantially on alert days. The average NO_2_ decrease was about 7.7 ± 0.1 μg m^−3^ (roughly 15% relative reduction), with inter-city decreases typically ranging from ∼3 μg m^−3^ in lower-impact regions up to ∼15 μg m^−3^ in the most affected areas. Our results show that SO_2_ concentrations exhibited only limited and inconsistent changes due to pollution alerts. On average, SO_2_ decreased by ∼2.6 ± 0.1 μg m^−3^ (a 13% drop due to a low baseline). Some cities observed SO_2_ decreases of >5 μg m^−3^ on alert days (e.g. Handan and Datong), but most cities showed little change or a slight increase (Fig. S4). The average impacts of pollution alerts on each city, along with associated uncertainties, are provided in Table S6.

### Reductions in PM_2.5_ due to pollution alerts led to decreased mortality

**Fig. 4.**
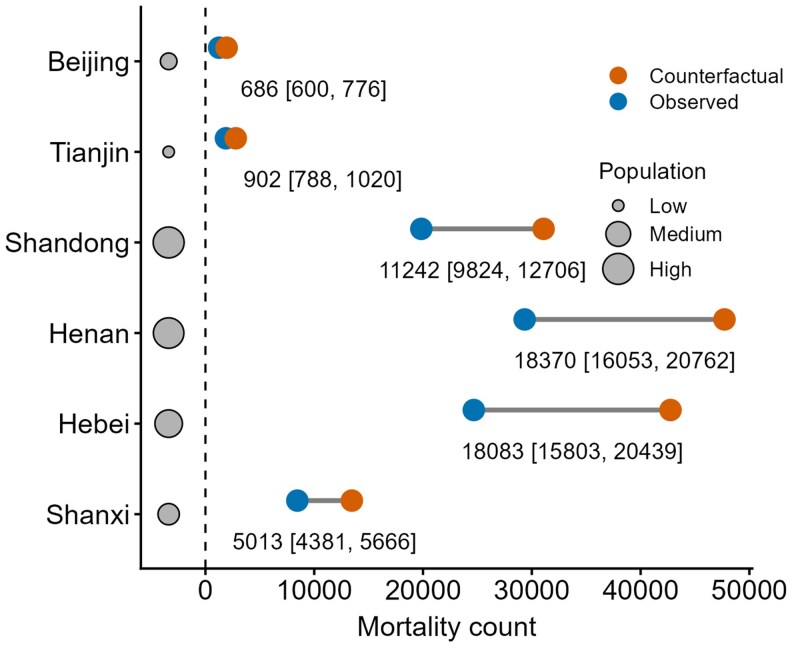
Province-specific short-term PM_2.5_-attributable mortality under observed versus counterfactual pollution alert scenarios. The observed scenario with implemented pollution alerts and a counterfactual scenario without alerts were compared. The differences between these scenarios, indicated by text below each segment with bracketed ranges “[.]” (which reflect different assumptions about baseline mortality rates), represent our estimate of the number of premature deaths avoided between 2018 and 2022 due to the pollution alerts.

Figure 4 shows that the implementation of air pollution alerts in China has yielded substantial public health benefits by preventing an estimated ∼54,000 ± 6,000 (∼11%) premature deaths attributable to short-term PM_2.5_ exposure over a 5-year period between 2018 and 2022. During periods when alerts were in effect, the acute mortality risk due to PM_2.5_ was reduced by an estimated 30–40% compared with counterfactual scenarios without alerts. Such benefit was especially significant in the most populous or heavily polluted regions. In Hebei Province, the rate of PM_2.5_-attributable mortality during alert days was about 42% lower (∼24,600 observed deaths) than the estimated ∼42,700 deaths expected in the absence of alerts. In Henan and Shandong, with their large population, we estimate roughly 18,400 and 11,200 premature deaths were avoided due to the pollution alerts, respectively. In contrast, two municipalities Beijing (∼690 avoided deaths) and Tianjin (∼900) saw relatively smaller health gains (∼3% of the total death avoided), consistent with their comparatively modest reductions in PM_2.5_ concentrations. City-level estimates of short-term PM_2.5_-attributable mortality for both observed and counterfactual alert scenarios are shown in Fig. S5.

## Discussion

### Methodological challenges in constructing no-alarm counterfactual scenarios

Evaluating the efficacy of pollution alerts has often been limited in scope and method. Many prior policy evaluation studies applied quasi-experimental designs as regression discontinuity or difference-in-differences to estimate impacts (26). These analyses commonly require a valid, unaffected “control” region, which is untenable when alerts recur regionally and violate model assumptions. Chemical transport models (CTMs) can in principle simulate air quality improvements under hypothetical emergency emission curtailments (7). However, such model-based analyses would need strong assumptions about the magnitude of emissions cuts and full public compliance during alert days (27), introducing great uncertainty and potential bias in linking observed air quality changes directly to the alerts.

This work handles these limitations through a data-driven framework using automated ML (AutoML). We develop ML models trained on local meteorology and temporal trends in the absence of any alert impacts. To account for bias caused by excluding pollution episodes in the training data, we introduce correction factors to recalibrate the predictions. Comparing observed concentrations on alert days with this dynamic, city-specific corrected baseline could isolate the alert's effect. Implementing the framework across multiple cities for consecutive years produces the first large-scale, multi-year estimates of alert effectiveness, increasing both attribution confidence and the generalizability of policy insights.

### Spatial variability in pollution alert effectiveness

Our results show that the effectiveness of pollution alerts in improving air quality and health benefits varied markedly across regions and pollutants (Figs. 3, 4, S3, and S4). This spatial heterogeneity indicates that alert effectiveness is context-dependent and driven by the local mix of emission sources and intervention strategies. Heavy industries such as steel mills, coal-fired boilers, and cement factories are well-documented contributors to dust and fine particles in cities with dense industrial activity (28, 29). In industrial hubs with poorer air quality (e.g. cities in Hebei), authorities mandate those polluting industries to shut down or cut output during alert days, following “differentiated” emission reduction strategies across grades by their performance (30). Such immediate end-of-pipe control measures directly target the dominate PM_2.5_ and PM_10_ sources, yielding marked drops in PM levels (Fig. 3). In contrast, more service-oriented cities with fewer large emitters experienced smaller, still measurable air quality improvements under alerts (e.g. Chengde and Weihai). This is also attributed to the relatively cleaner air quality baseline in these cities, with fewer alerts, shorter duration, and more limited scope of control measures implemented (Figs. 1B and 2A).

Traffic-related emissions, particularly nitrogen oxides (NO_X_ = nitrogen oxide NO + NO_2_), from on-road vehicles are highly responsive to short-term interventions such as vehicle restrictions or enhanced inspections (31). In megacities with dense vehicle fleets such as Beijing, pollution alerts trigger traffic emergency restrictions. For example, all light-duty vehicles certified to China stage III or newer must follow an odd–even license plate schedule, while high-emitting older cars (China stage I/II) are forbidden from driving (32). This may explain larger reductions in ambient NO_2_ concentrations during alerts by roughly 25%, whereas most other cities saw declines of <18%.

SO_2_ emissions, primarily from fossil fuel combustion, are more effectively controlled by long-term measures such as the use of low-sulfur coal and flue gas desulfurization systems (33). Current sulfur content in bituminous coal is <0.5%, even lower according to the local standard (20). As a result, SO_2_ emissions in most cities have been reduced and reached the compliance level, with little change during the alert days. Moreover, previous studies also reported that jurisdictions with strong enforcement mechanisms, including real-time monitoring and penalties for violations, can make faster progress in cutting emissions (34). In contrast, regions with limited financial resources or political barriers to strict enforcement faced challenges such as lower compliance rates and weaker pollution reductions (35).

### Nonlinear relationship between alert severity and air quality outcomes

Here, we did not find a clear linear relationship between color-coded alert severity and air quality outcomes (Fig. 3). Increasing alerts from moderate to severe did not lead to significant additional reductions in pollutant levels, as the average effects fell within the range of uncertainty (Fig. S6). Theoretically, a higher-tier alert (e.g. upgrading from orange to red) entails more stringent emission curbs (7, 36) and thus should lead to greater pollution reductions. Several factors likely explain this nonlinearity. An important reason is the timing and sequence of alerts. Red alerts are issued only after pollution has already escalated for several days (frequently an orange alert precedes a red, Fig. 2A). By that stage, a large build-up of PM is in the air, and the additional measures under a red alert could prevent further worsening rather than instantly clean the air. Additionally, the benefits of an intervention take time to materialize, and Xue et al. (37) noted that the maximum effect of emergency measures occurs 48–72 h after implementation. Although red alerts mandate stricter emission cuts, the incremental stringency may not translate immediately and linearly into concentration reductions due to lag effects, especially when alerts are rapidly escalated and de-escalated within consecutive time periods.

Second, many control measures overlap across alert levels, and major pollution sources (industrial activities, vehicle use, etc.) might already be partially restricted from the preceding orange alerts (38), leaving less room for additional improvement. The remaining pollution may come from sources that are harder to eliminate completely (such as dispersed residential heating or cross-boundary haze) (7). Third, there might be practical enforcement challenges that level off the effectiveness of higher alerts. Stricter measures (e.g. red) are harder to enforce universally if the requirements become too disruptive (39), or local authorities might enforce orange and red measures with similar stringency in practice due to resource constraints, leading to smaller than expected differences or even reverse patterns across alert levels.

### Implications

With air pollution responsible for over 1 million premature deaths annually in China (40), strategies that deliver rapid exposure reduction are critical. This study offers the first multi-year, multi-city evidence that tiered pollution alerts with actionable interventions can significantly lower PM_2.5_ levels and thus prevent premature deaths. Unlike isolated “natural experiments” of short-term blue-sky campaigns during special events such as Asia-Pacific Economic Cooperation (APEC), our results show that the alert system provides a repeatable mechanism to mitigate pollution spikes. These findings underscore the value of embedding short-term responses into routine air quality management, particularly during winter haze seasons when health risks are highest. Moreover, the effectiveness varies by region and pollutant, indicating the need for locally adaptive alert strategies that target dominated emission source characteristics and possibly coordinated regional action (e.g. joint measures across neighboring cities during widespread smog) to maximize the alert system's benefits (36). While pollution alerts are no substitute for long-term structural reforms, they provide an essential second line of defense on days pollutant levels would otherwise spike. Our findings offer solid support for bolstering such emergency protocols not only within China but also in rapidly urbanizing regions or countries that face similar air quality crises, such as parts of Africa and Central and Southern Asia.

Our study shows that pollution alerts have significant environmental and health benefits; however, they also present some challenges. Firstly, Fig. 2A illustrates that the timing and severity of alerts are highly variable and often discontinuous across different cities. There is a reason for the skepticism that this irregularity could complicate production planning for industries such as power plants, steel mills, chemical factories, and small- and medium-sized enterprises (SMEs). Frequent and fast-changing severity alerts require these businesses and other major emitters to curtail production on short notice, leading to output losses and costly shutdown–restart cycles. Secondly, these disruptions may also translate into lost revenue streams, workforce idling, and broader supply chain interruptions for affected sectors (41). Previous studies found that it is particularly challenging for SMEs to handle the economic impact of these sudden regulatory shocks due to their limited financial buffers and narrower operational flexibility (41, 42). Furthermore, studies have also reported challenges that may erode alert effectiveness over time, such as rebound effects in emissions and public desensitization to repeated pollution warnings, often referred to as “alert fatigue” (11, 12, 43). Thirdly, despite the temporary emission cuts and measurable pollutant reductions achieved during alerts, for most cities, air pollution concentrations remain extremely high during alert days, which always exceeded WHO AQG (Fig. 2B). This persistent noncompliance indicates that although alerts provide short-term mitigation, they are insufficient as a substitute for long-term, sustained structural emission control measures to achieve lasting improvements in air quality.

### Limitations and future research

While our evaluation reveals clear benefits of China's pollution alert system, several limitations of the analysis must be acknowledged. This study evaluated and employed correction factors derived from historical data, but it still may not fully reflect structural changes in emission sources or control measures over time. In addition, the baseline model excluded data during transition periods around pollution alert implementation (to isolate policy effects); while necessary, this omission may cause rapid pollution fluctuations during those periods to be underrepresented in the analysis. Moreover, while the health benefit estimates from PM_2.5_ reductions due to pollution alerts are useful for policy, they rely on a few key assumptions. We assumed a log-linear concentration-response function as the same as previous epidemiological studies (44, 45) (see Methods), whereas real-world response may be nonlinear at high-PM_2.5_ levels (46). Also, health outcome calculations relied on national-average mortality rates across the population, which ignores regional or demographic differences in underlying health risk (47). Such uncertainties do not undermine the value of our results; rather, they highlight the need to interpret the estimates with approximate caution and to refine future assessments as better data become available.

Our health-impact estimates use city-average PM_2.5_ combined with total city population and therefore do not capture intra-urban spatial gradients or population-weighted exposure. Prior work shows that coarser or unweighted exposure metrics can shift health-burden estimates relative to population-weighted, higher-resolution models, with sign and magnitude depending on how pollution covaries with population density (48, 49). We expect any bias here to be small relative to other uncertainties (e.g. concentration-response function and baseline mortality) (50), and our qualitative findings should hold because we estimate differences between observed and counterfactual concentrations. Nevertheless, integrating gridded population data with high-resolution air quality data and, where feasible, microenvironmental adjustments (e.g. indoor infiltration and time-activity) would provide a more precise estimate of avoided deaths and is a priority for future work.

The results in this study open up several avenues for future research, including efforts to enhance our understanding of how pollution alerts can improve health outcomes, such as reducing hospital visits, morbidity, and mortality, through direct epidemiological studies. Another important direction is to understand the effect of pollution alerts on public behavior. Our research shows that these alerts correspond to fluctuations in air pollution levels, indicating changes in emission activities. However, it remains crucial to know whether individuals actually take notice of the alerts and deliberately adjust their behavior. Previous studies indicate that only ∼57% of adults who recognized that local air quality was poor made any changes in response (51), and responses can vary across demographic groups in age (52). Finally, our study focuses on the impact of pollution alerts in China, a developing country with relatively strong regulatory and enforcement capacity. It is unclear, however, whether similar effects would be observed in other developing countries that are poorer and/or have higher baseline air pollution, especially without complementary measures to support and engage the affected communities (12, 53).

## Methods

### Surface air quality and meteorological data

We obtained daily surface data for PM_2.5_, PM_10_, NO_2_, and SO_2_ from the High-resolution and High-quality Ambient Air Pollutants Dataset for China (CHAP) (14–17). CHAP integrates ground monitors, satellite retrievals, reanalysis data, and CTM outputs and employs an AI framework to capture fine-scale air pollution spatiotemporal variability. O_3_ was excluded because its levels were generally low in winter due to reduced photochemical reactions. To calculate citywide pollutant concentrations, we overlaid the 1-km CHAP data on municipal boundaries (National Basic Geographic Information Centre, https://www.webmap.cn/commres.do?method=result100W) and took area-weighted means. This approach preserves spatial heterogeneity and reduces reliance on sparse fixed monitors.

Air pollution alert dates and severity levels were collected from official municipal or provincial web portals for 57 northern Chinese cities (two municipalities and 55 prefectures). The sample includes Beijing, Tianjin, and four heavily industrialized provinces (Henan, Hebei, Shandong, and Shanxi; Table S1). For meteorological variables, we used hourly fields from the ERA5 (with resolution ∼31 km) and ERA5-Land (∼9 km) reanalysis datasets produced by the European Centre for Medium-Range Weather Forecasts (ECMWF) (54). The factors from ERA5 include surface pressure and boundary layer height, and the factors from ERA5-Land include wind speed, wind direction, surface temperature, and total precipitation. These factors strongly influence pollutant dispersion and accumulation. We aggregated ERA5 and ERA5-Land data to daily means and spatially averaged them over each city's polygon.

### Quantifying air pollution changes during alert periods

Estimating the impact of alerts on air pollution requires constructing a BAU pollution level for days when alerts were enacted. Simply omitting alert days and predicting those days with an unconstrained model is inadequate because (i) pollution alerts occur on the worst days, when baseline emissions (e.g. winter heating and industrial activity) and meteorological stagnation can already elevate pollution; and (ii) other winter measures (e.g. routine industrial curtailments) may occur concurrently and independently reduce emissions. Models trained on all data will incorporate the impacts of alerts, while models trained only on nonalert “clean” days without alerts may introduce sample-selection bias and underestimate the occurrence of severe events. To address these issues, we designed a two-step ML framework that (i) estimates BAU concentrations from contemporaneous, nonalert data and (ii) applies a correction for extreme winter events.

#### Base model development

We trained city-specific ML models to estimate daily BAU pollutant concentrations (i.e. in the absence of pollution alerts) using temporal and meteorological variables. Our input features included trend and year to capture long-term changes, month to represent seasonal variation, day of the week to account for weekday–weekend activity patterns, and above-mentioned meteorological factors.

Using the H2O.ai's AutoML framework (55), we tested an ensemble of 30 candidate algorithms (e.g. gradient boosting, random forest, and deep learning) and selected the model minimizing the loss function on held-out data. Importantly, the training and testing set (2018–2022) excluded all days influenced by alerts and buffer windows (1 day before and 3 days after each alert). This ensures the base model learns BAU conditions without being contaminated by interventions. The best-performing function, $\hat{\;f}(.)$, that minimized prediction errors can be expressed as:


(1) $$\hat{\;f}(.)=\mathrm{argmi}n_{\;f(.)\in F}E_{x∼D_{\mathrm{train}\setminus D_{\mathrm{excluded}}}}[\ell (\;p,f(x))].$$

Here, F represents the set of candidate models generated by AutoML; $D_{\mathrm{train}\setminus D_{\mathrm{excluded}}}$ is the training dataset excluding all alert-influenced periods $D_{\mathrm{excluded}}$; ℓ(p,f(x)) is a loss function measuring the difference between observed pollutant *p* and model predictions f(x); and *x* represents the set of input features. The excluded dataset $D_{\mathrm{excluded}}$ is defined as:


(2) $$D_{\mathrm{excluded}}=U_{\mathrm{alert}\in A}(t_{\mathrm{start}}^{\mathrm{alert}}-1,t_{\mathrm{end}}^{\mathrm{alert}}+3)$$

where $t_{\mathrm{start}}^{\mathrm{alert}}$ and $t_{\mathrm{end}}^{\mathrm{alert}}$ denote the start and end times of each alert, respectively.

We used an 80/20 data split for model training and testing, and we evaluated model performance using the index of agreement (IOA), correlation coefficient (*r*), and rms error. Most city models achieved IOA > 0.7 (Table S2).

#### Base model correction

Excluding alert days and buffers in 2018–2022 can leave the base model trained on a “too-clean” sample in cities where alerts cluster in winter, risking underestimate BAU during the very conditions when alerts occur. Here we derived city-specific correction factors using 2013–2016 data (no alerts or alerts were rare) as a historical reference.

Firstly, we linked the correction to each city's alert intensity. For every city, we worked out how much of the winter heating period (November–March) was removed from the 2018–2022 training data because of alerts and their buffers. This gives a city-specific share of winter days that were excluded in the 2013–2016 period (more pollution alerts meant more data exclusions). The number of excluded days and the corresponding quantitative thresholds for each city are reported in Table S3. Subsequently, we trained models on the remaining 2013–2016 data with the same predictors as in the base model. Thirdly, we predicted the excluded extreme pollution days and computed the ratio of observed-to-predicted concentrations, which served as the correction factor (Fig. S7). We then applied these factors to the base model's predictions during alert periods (Fig. S9). The difference between observed concentrations and corrected BAU predictions was taken as the net effect of pollution alerts (Fig. S3). To ensure robustness, we dropped outlier values of these differences when calculating the average policy effect.

### Robustness analysis

To evaluate the stability of our findings, we tested two sources of uncertainty, including the transferability of the correction factors and the sensitivity to the pollution data source used as the dependent variable in our counterfactual models. We isolate the component under evaluation and leave all other elements of the pipeline unchanged for both scenarios.

#### Correction factor uncertainty analysis

We evaluated whether correction factors derived from historically higher-pollution winters remain applicable when applied to later years with lower absolute concentrations (Fig. S10). For each city, we trained identical ML counterfactual models twice, once using 2013–2014 and once using 2015–2016 after excluding extreme high-PM_2.5_ days during heating seasons from the training set. The same modeling processes and input features were employed as in previous steps. We then used the correction factors derived from 2013 to 2014 to correct predictions for 2015–2016 high-PM_2.5_ windows and compared these corrected values to observations. As shown in Figs. S11–S13, the correction factors systematically improved accuracy under high-pollution conditions relative to the uncorrected base models. For most cities, the residual uncertainties of corrected predictions are <10%, and the small remaining bias is conservative (predictions slightly below observations), which implies our estimated effects are, if anything, understated rather than exaggerated.

#### Pollution data uncertainty

In this study, we used the CHAP dataset because it provides spatially complete, daily coverage for all pollutants across all 57 cities over the required multi-year window. This comprehensive record enables the derivation of city-specific winter correction factors from a long and internally consistent historical period, ensuring comparability in evaluating alert effectiveness. The national air quality monitoring network became sufficiently dense and quality-controlled after 2015 (56); relying on station data alone would, therefore, leave too short and incomplete a preperiod for stable factor estimation. To test whether our results depend on the use of CHAP rather than direct ground observations, we repeated the full analysis using ground-station daily averages for 2018–2022 as the dependent variable while applying the CHAP-derived correction factors from 2013–2016.

The CHAP-based and the station-based effects are in close agreement across cities, pollutants, and alert levels. At the provincial scale, the estimated pollution reductions essentially remained (Figs. 3, 4, S14, and S15). For example, both datasets indicated PM_2.5_ declines of about 30–35 μg m^−3^ in Tianjin and 40–60 μg m^−3^ in Henan, while Beijing exhibited comparable decreases across alert levels. Larger deviations occurred mainly in cities with few alerts or complex monitoring conditions, yet these did not change the overall conclusions. For estimated mortality benefits due to PM_2.5_ declines, both methods yielded comparable results in Henan, Hebei, and Shandong, on the order of 11–18 thousand deaths with uncertainties of about ±10–12%. For Tianjin and Beijing, estimates were roughly 900 and 700 deaths, respectively, and only minor discrepancies appeared in Shanxi, but all differences remained well within the estimated error margins. Overall, these tests indicate that pollution alerts reduce air pollution, delivering measurable health benefits independent of the correction method or data source.

### Estimating the public health impact of PM_2.5_

PM_2.5_ levels are the main trigger for air pollution alerts, and short-term exposure to high-PM_2.5_ concentrations has been robustly associated with an increased risk of mortality. To quantify the public health benefits of pollution alerts that reduce PM_2.5_ levels, we estimated the relative risk (RR) of mortality using a log-linear concentration-response function (45):


(3) $$\mathrm{RR}=e^{\beta \Delta PM_{2.5}}$$

where *β* represents the concentration-response factor, which is the estimated slope from the log-linear relation between PM_2.5_ and mortality. The term $\Delta PM_{2.5}$ is the change in PM_2.5_ concentration (in μg m^−3^). The premature mortalities attributed to the short-term PM_2.5_ during the alert periods can be calculated as:


(4) $$\mathrm{Mor}t_{\mathrm{base}}=\gamma_{0}\underset{i}{\sum }\;\underset{j}{\sum }\;\mathrm{po}p_{i}(1-{\mathrm{RR}}_{\mathrm{obs},i,j}^{-1})$$

and the reduction in premature mortalities attributed to the implementation of pollution alerts is calculated as:


(5) $$\Delta \mathrm{Mort}=\gamma_{0}\underset{i}{\sum }\;\underset{j}{\sum }\;\mathrm{po}p_{i}({\mathrm{RR}}_{\mathrm{obs},i,j}^{-1}-{\mathrm{RR}}_{\mathrm{cf},i,j}^{-1}),$$

_where_  $\gamma_{0}$ is the national-average mortality rate in China in 2018 (https://vizhub.healthdata.org/gbd-results); $\mathrm{po}p_{i}$ is the population of city *i*, as obtained from the local branch of the National Bureau of Statistics; ${\mathrm{RR}}_{\mathrm{obs},i,j}^{-1}$ and ${\mathrm{RR}}_{\mathrm{cf},i,j}^{-1}$ are the RR on day *j* for city *i* under the observed (with alerts) and counterfactual (without alerts) scenarios, respectively. We employed an RR value of 1.0065 (95% CI: 1.0044 − 1.0086) for all-cause mortality per 10 μg m^−3^ increase in PM_2.5_, based on short-term exposure studies (44, 57). In addition, we assume that PM_2.5_, concentrations below 2.4 μg m^−3^ have no measurable adverse health effects (58). Daily estimates of premature mortality are aggregated into annual sums for further analysis. Uncertainties in these health burden estimates are quantified by considering the variability in the baseline mortality rates.

## Supplementary Material

pgag054_Supplementary_Data

## Data Availability

Air pollution dataset: High-resolution and High-quality Ambient Air Pollutants Dataset for China (CHAP) https://weijing-rs.github.io/product.html. Meteorological data are available at https://www.ecmwf.int/en/forecasts/dataset/ecmwf-reanalysis-v5 and https://www.ecmwf.int/en/era5-land. Data and scripts used to produce this analysis are available at this GitHub repository: https://github.com/clnair-ascm/PolluitonAlert.
