# Supplementary material for: Significant benefits of pollution alerts for cleaner air and better health
Source: PNAS Nexus. 2026 Mar 3;5(3):pgag054. doi: 10.1093/pnasnexus/pgag054 (PMC12988777; doi:10.1093/pnasnexus/pgag054)
Supplement: pgag054_Supplementary_Data [file pgag054_supplementary_data.zip › PNASNEXUS-PNASNEXUS-2025-00851RR-s02.docx]

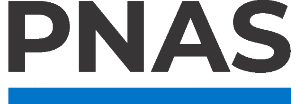


**Supplementary Information for**

Significant benefits of pollution alerts for cleaner air and better health

Yuqing Dai ^a*^, Juncheng Qian ^a^, Yue Yang ^b^, Bowen Liu ^c^, Shuyu Li ^d^, Kun Zhang^e^, Qiaorong Xie ^f^, Chengxu Tong ^a^, Ying Chen ^a^, A. Rob MacKenzie ^a^, Zongbo Shi ^a^

^a^ School of Geography, Earth and Environmental Science, University of Birmingham, Birmingham, B15 2TT, U.K.

^b^ China Metallurgical Industry Planning and Research Institute, Beijing 10013, China

^c^ Department of Management, Business School, University of Birmingham, Birmingham, B15 2TT, U.K.

^d^ Department of Economics, Business School, University of Birmingham, Birmingham, B15 2TT, U.K.

^e^ School of Environmental Science and Engineering, Southern University of Science and Technology, Shenzhen 518055, China

^f^ Department of Chemistry, Purdue University, West Lafayette, Indiana 47907, U.S.

*** Corresponding authors:**

Yuqing Dai

**Email:** [y.dai.2@bham.ac.uk](mailto:y.dai.2@bham.ac.uk)

**This PDF file includes:**

Supplementary Information

Figures S1 to S16

Tables S1 to S9

**Supplementary Information**


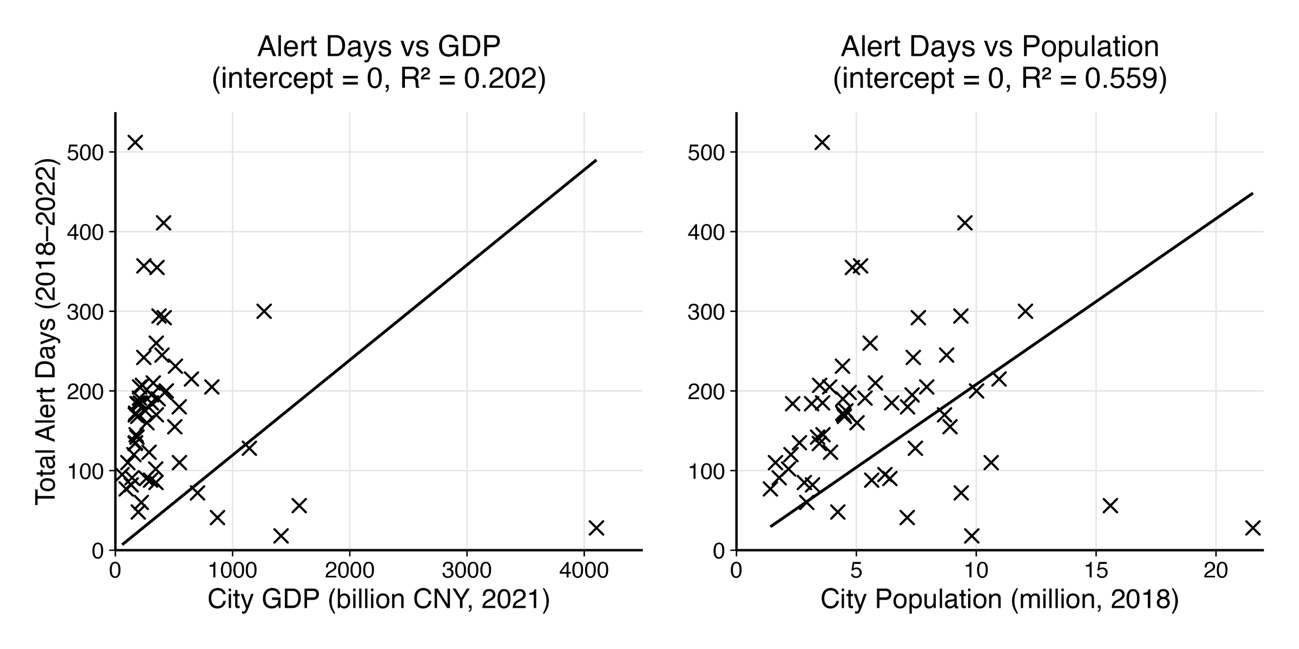


**Fig. S1.** **Relationships between total pollution alert days and socioeconomic indicators across Chinese cities.** Scatter plots show the relationship between the total number of pollution alert days (2018–2022) and city gross domestic product (GDP, 2021) as well as population size (2018). Each cross represents one city.


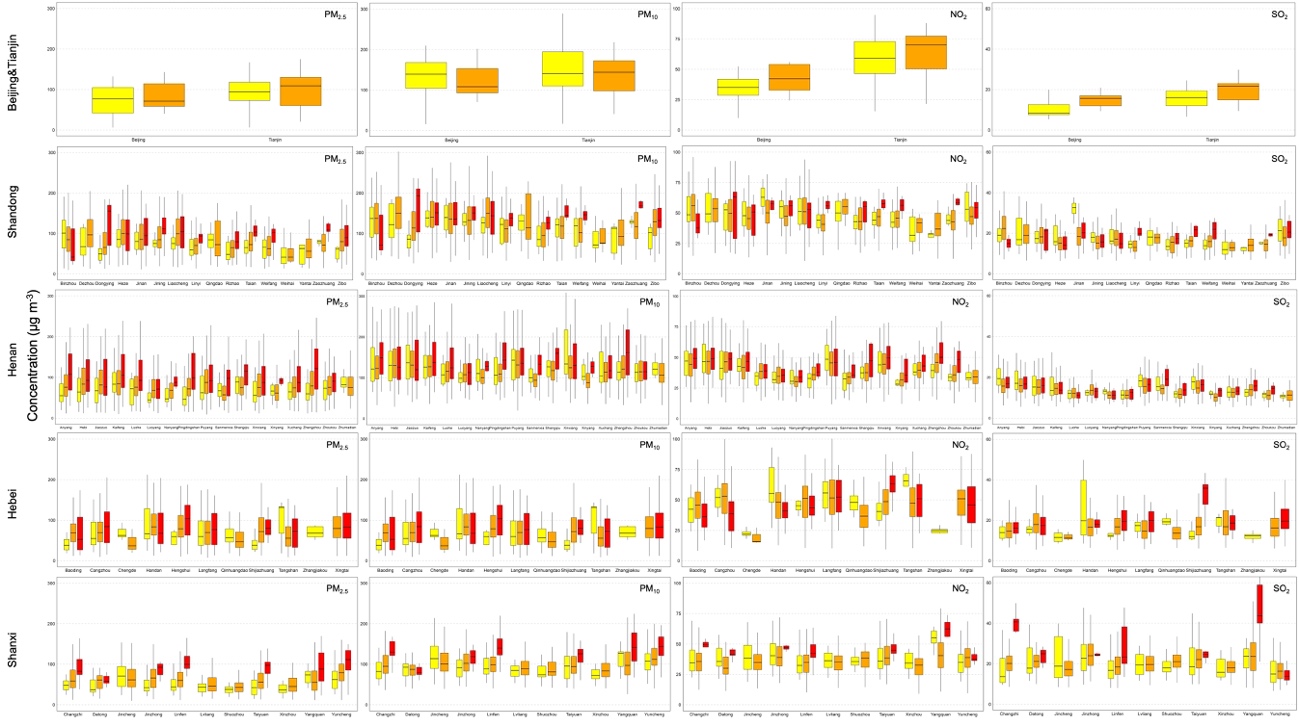


Fig. S2. City-specific observations of ground-level air pollution during pollution alert periods. Box plots show observed ambient pollutant concentrations in Chinese cities during pollution alert periods from 2018 to 2022. Cities are grouped by province, with municipalities (Beijing and Tianjin) noted separately. Colors represent different pollution alert levels: yellow, orange, and red, indicating varying alert severity.


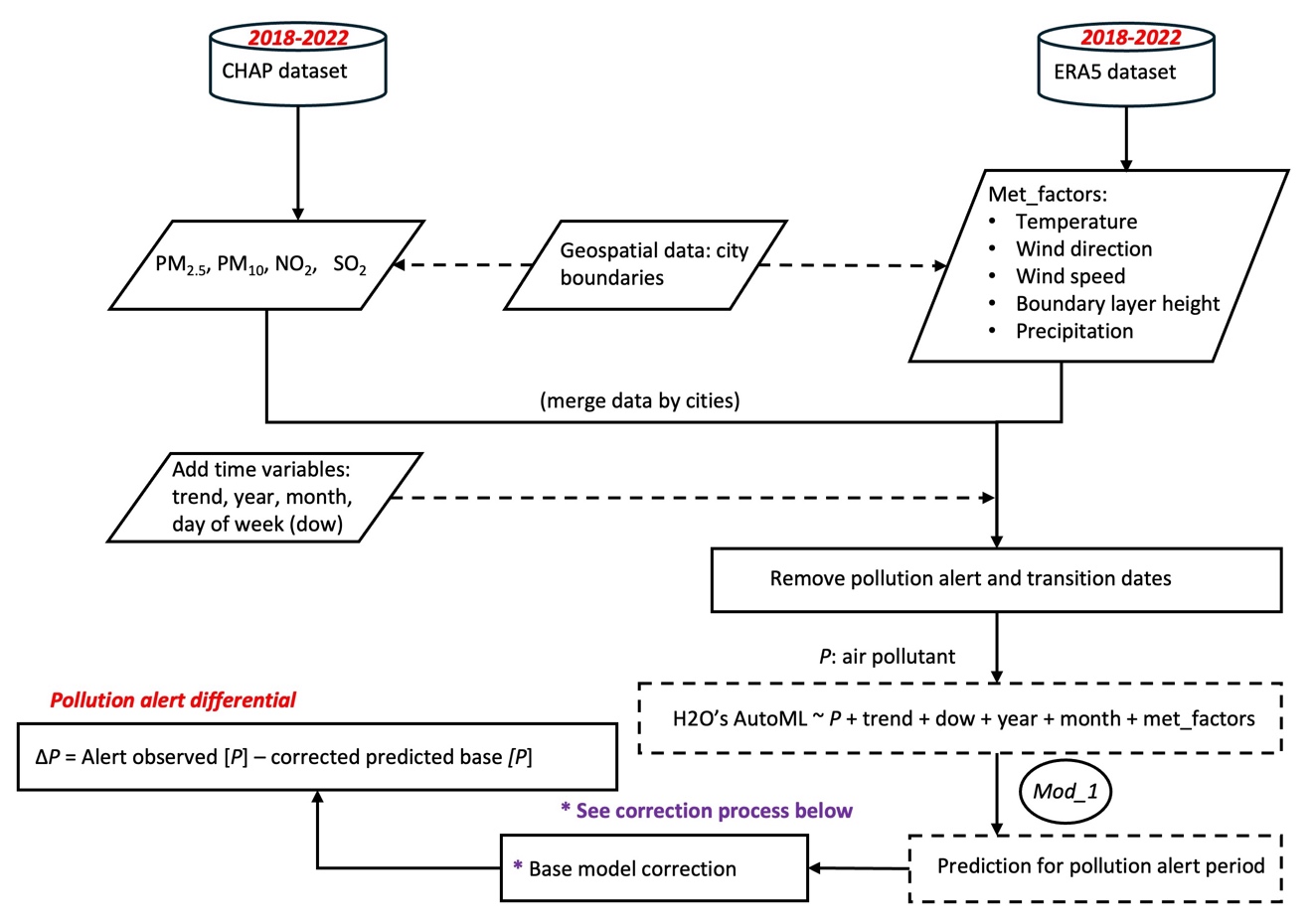


**Fig. S3. Methodological workflow for pollution alert analysis.** Air pollutants were sourced from the High-resolution and High-quality Ambient Air Pollutants Dataset for China (CHAP), and meteorological data were obtained from the ERA5 reanalysis by the European Centre for Medium-Range Weather Forecasts (ECMWF) for 2018-2022. Geospatial information was used to align data with target cities. The pollution alert differential was calculated as the difference between observed pollution levels on alert dates and predicted pollution levels after correction. To account for weather variability, pollution predictions were generated using a gradient boosting model from H2O’s AutoML platform.


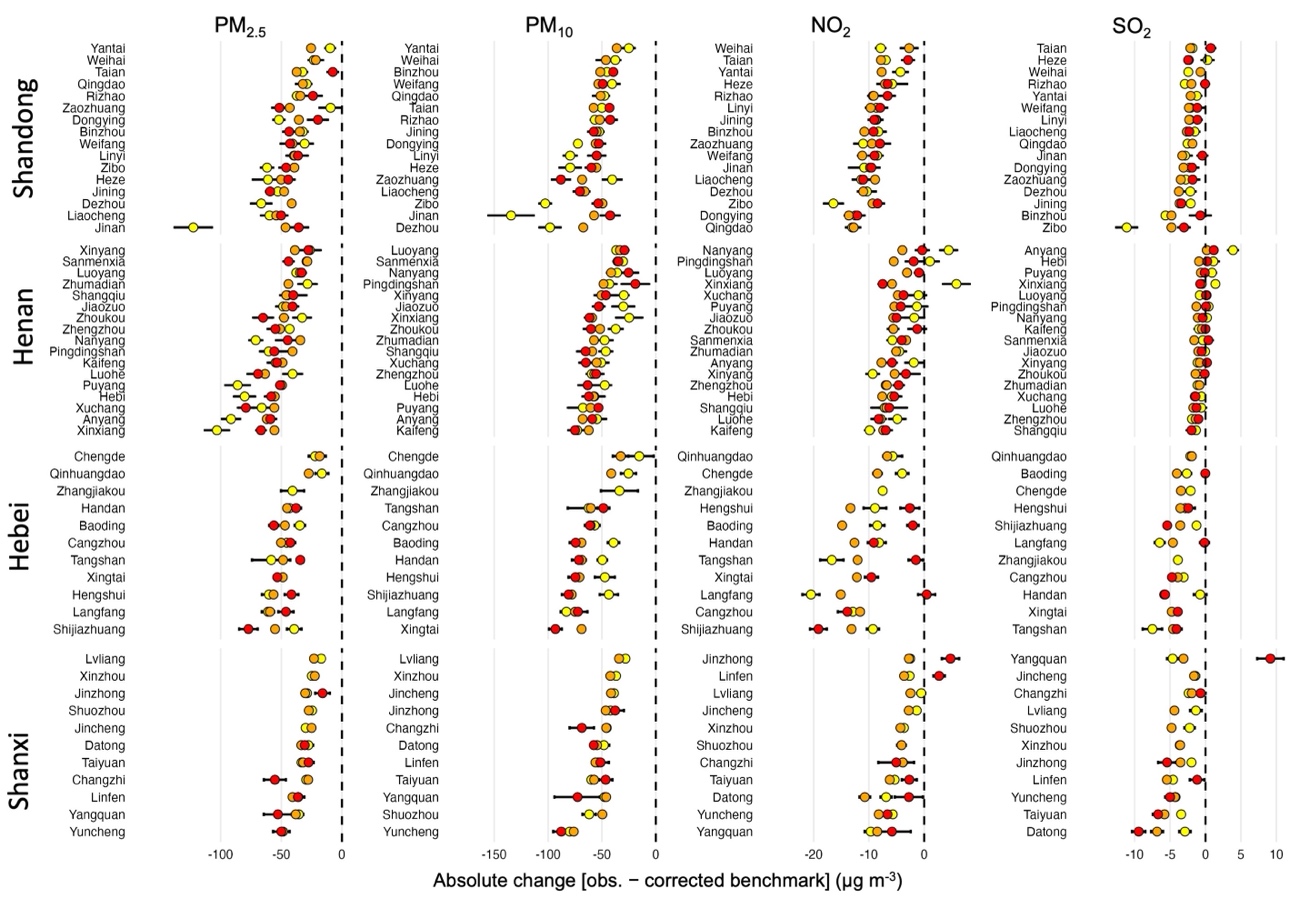


Fig. S4. City-specific anomalies in ground-level air pollution during pollution alert periods. Differences between observed ambient pollutant concentrations (obs.) and predictions from a base model (trained on 2018-2022 data, excluding alert and transition dates, after correction) during alert periods are shown for 57 Chinese cities with standard errors (SE, error bars). Both absolute (A) and relative (B) changes are presented. Cities are grouped by province, and an asterisk (*) denotes municipalities directly under the central government (Beijing and Tianjin). Points show cross-city mean values for each alert tier (color-coded), and error bars represent standard errors (SE) calculated from variability among city-level estimates.


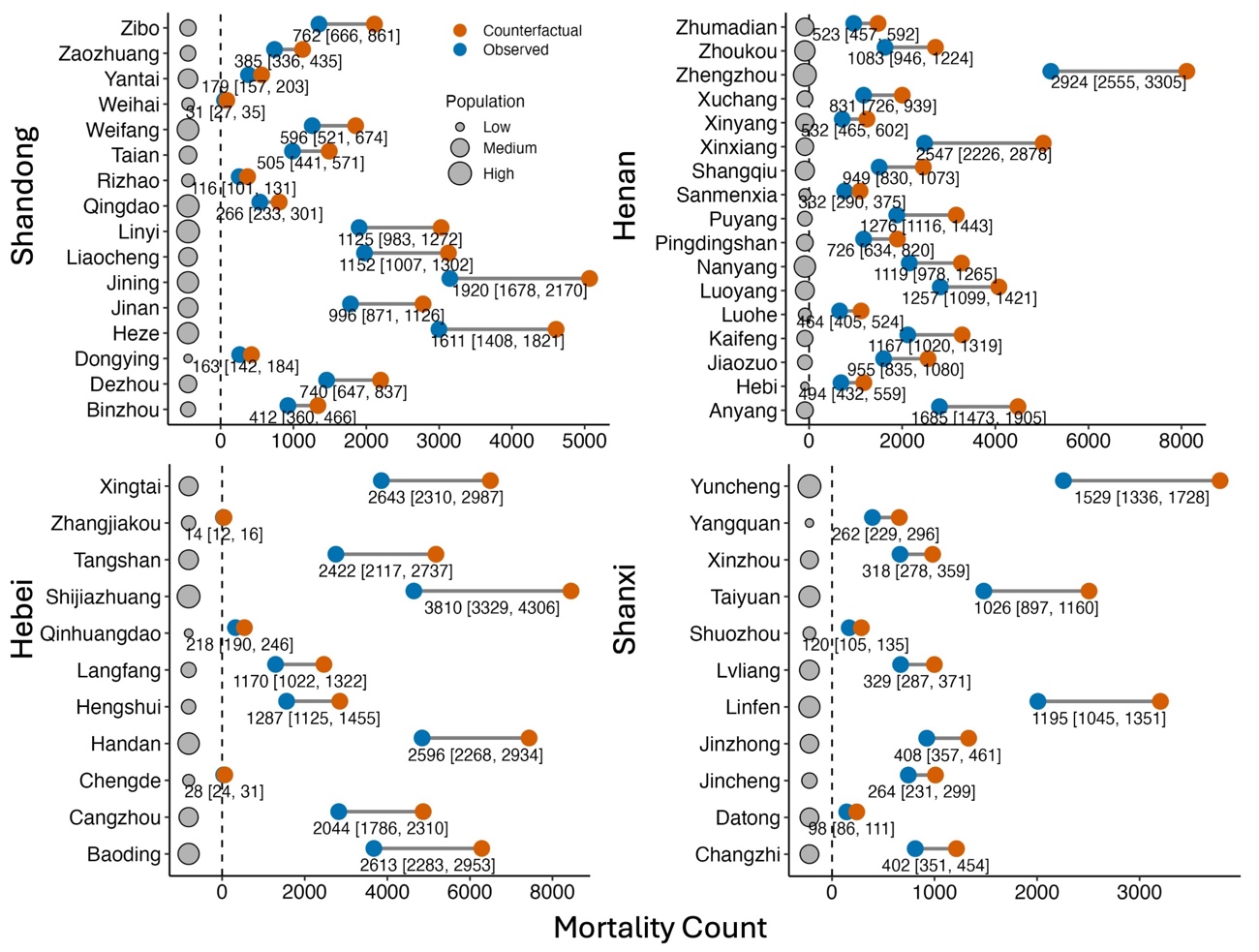


**Fig. S5. City-Specific Short-term PM_2.5_-Attributable Mortality Under Observed Versus Counterfactual Pollution Alert Scenarios.** The observed scenario with implemented pollution alerts and a counterfactual scenario without alerts was compared. The differences between these scenarios, indicated by text below each segment with bracketed ranges (which reflect different assumptions about baseline mortality rates), represents our estimate of the number of premature deaths avoided between 2018 and 2022 due to the pollution alerts.


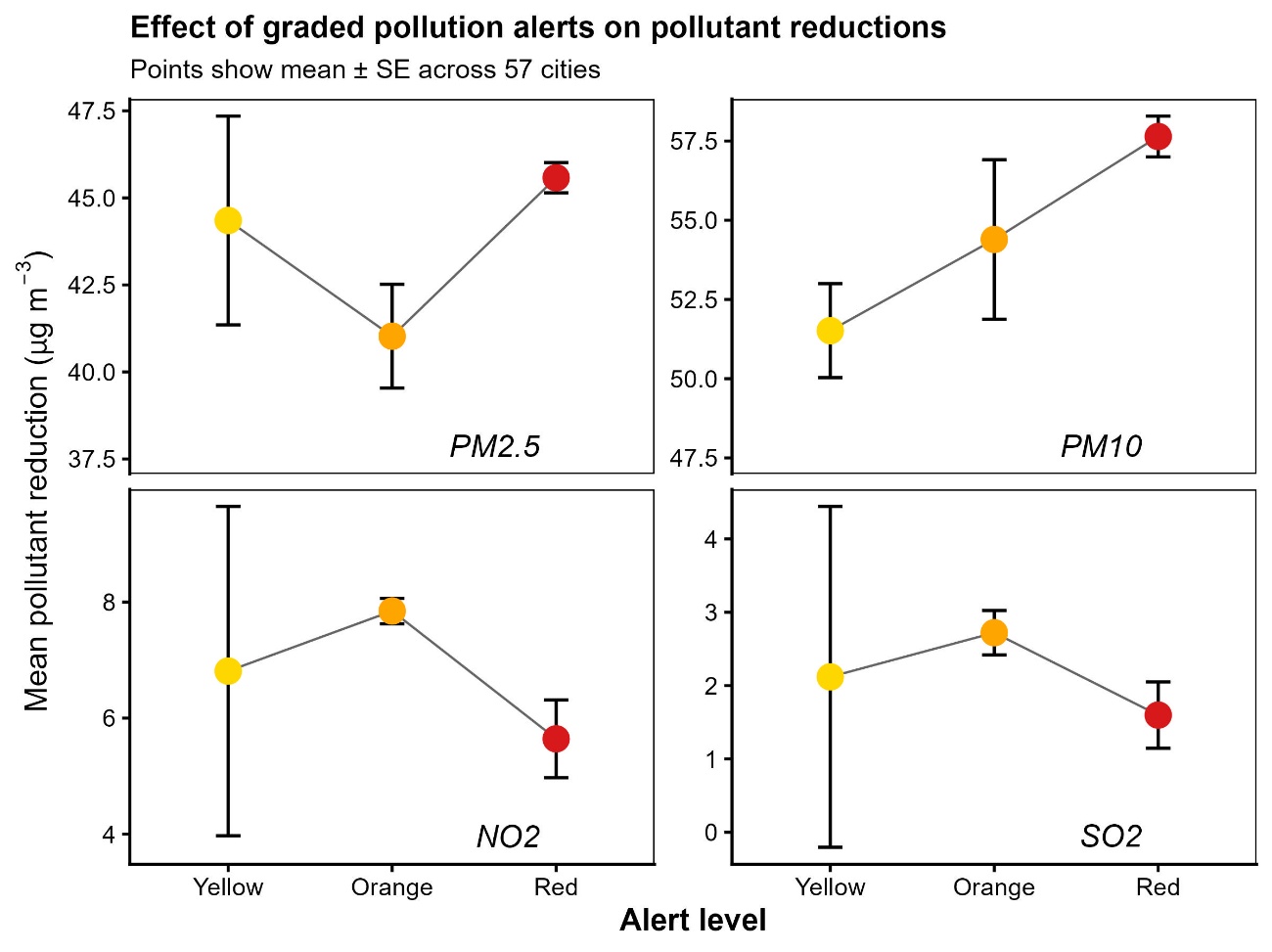


**Fig. S6. Effect of Graded Pollution Alerts on Pollutant Reductions.** Mean pollutant reductions (± SE) across 57 cities are shown for different alert levels (yellow, orange, and red). Each panel represents a pollutant (PM_2.5_, PM_10_, NO_2_, and SO_2_). The points indicate the average reduction in pollutant concentrations during alert days relative to counterfactual non-alert conditions, with error bars representing standard errors of the mean across cities.


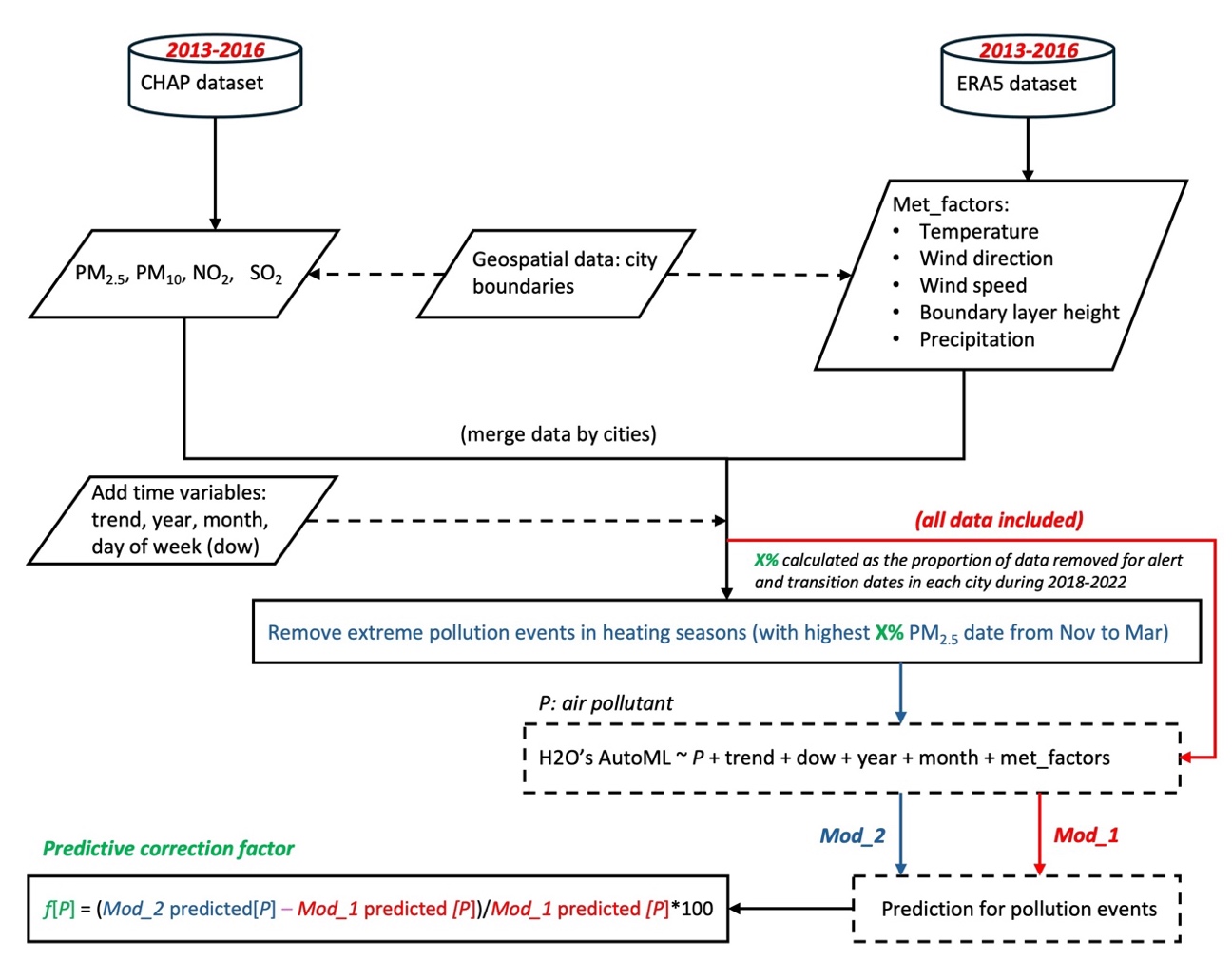


**Fig. S7. Methodological workflow for base model correction.** Air pollutants were sourced from ChinaHighAirPollutants (CHAP), and meteorological data were obtained from the ERA5 reanalysis by ECMWF for 2013-2016. Geospatial information was used to align data with target cities. The concentration differential was calculated as the difference between observed pollution levels on extreme pollution dates and predicted pollution levels after correction. To account for weather variability, pollution predictions were generated using a gradient boosting model from H2O’s AutoML platform.


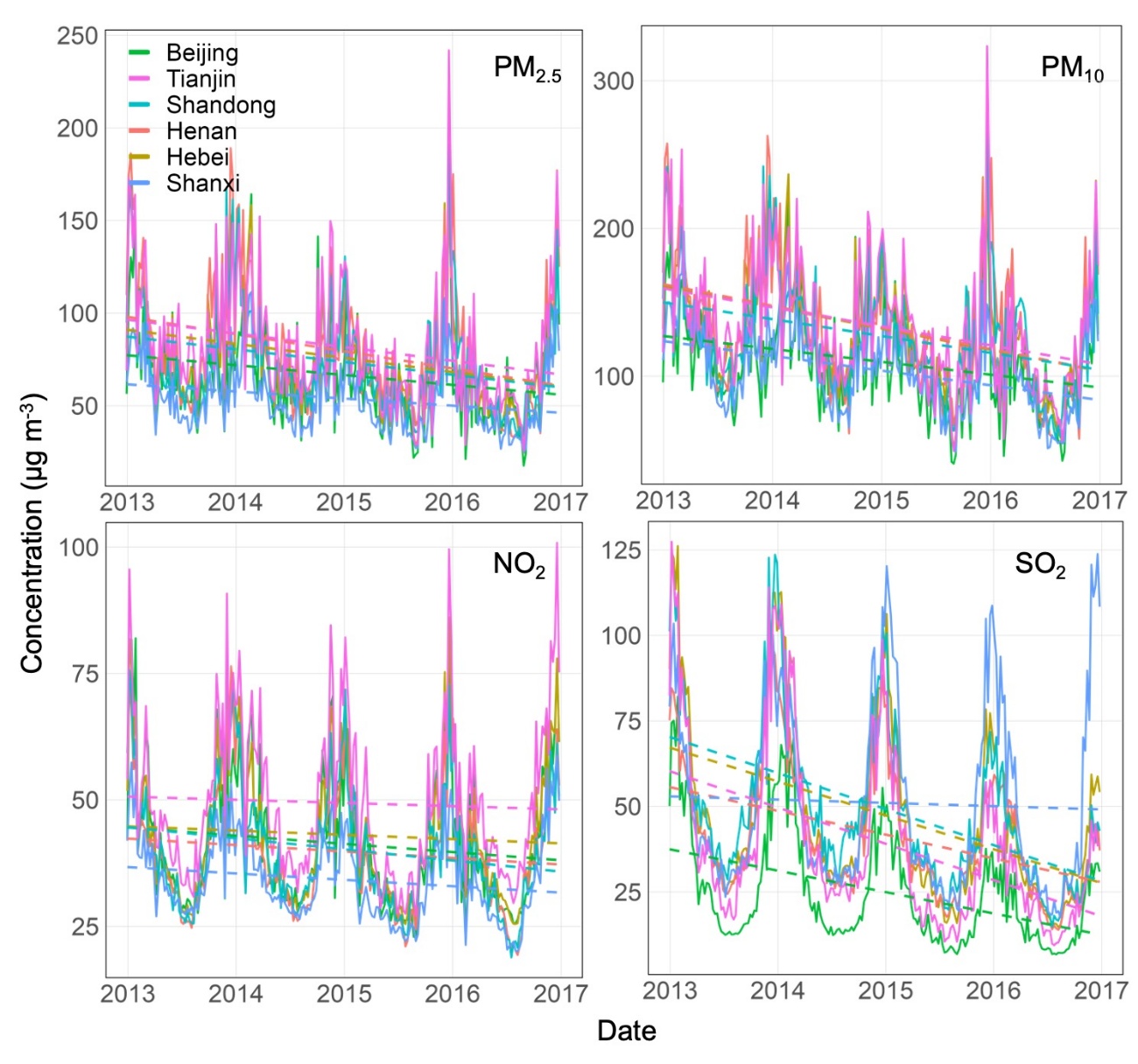


**Fig. S8. Weekly trends of air pollutant concentrations by province between 2013 and 2016.**


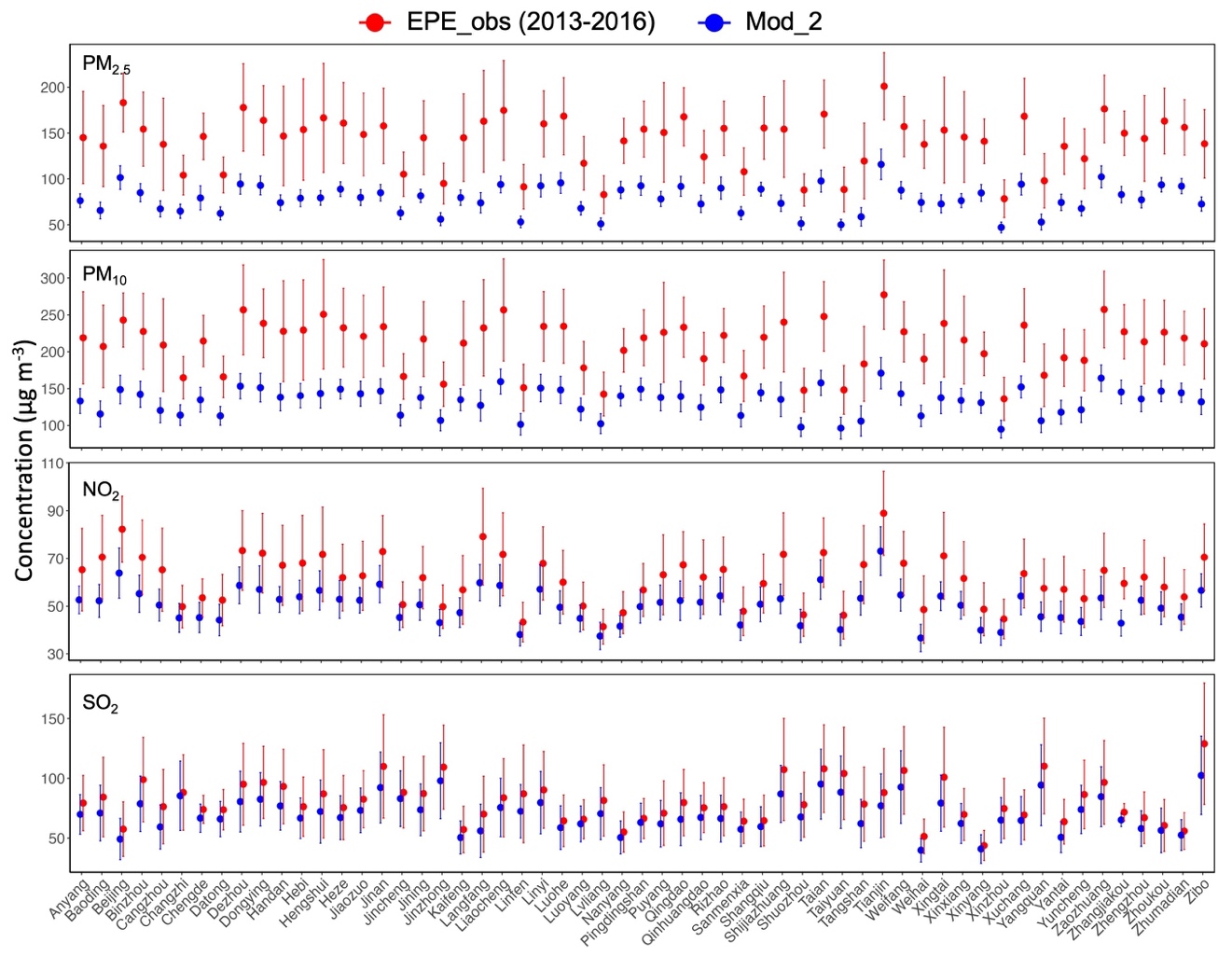


Fig. S9. Concentrations of observed and modeled pollutants across different cities between 2013 and 2016. The base model (mod_2) underestimated pollutant concentrations when extreme pollution episodes (EPE) in winter were excluded. The differences between observed and modeled values were used to derive correction factors for the base model (mod_1, Fig. S2) for each city.

**
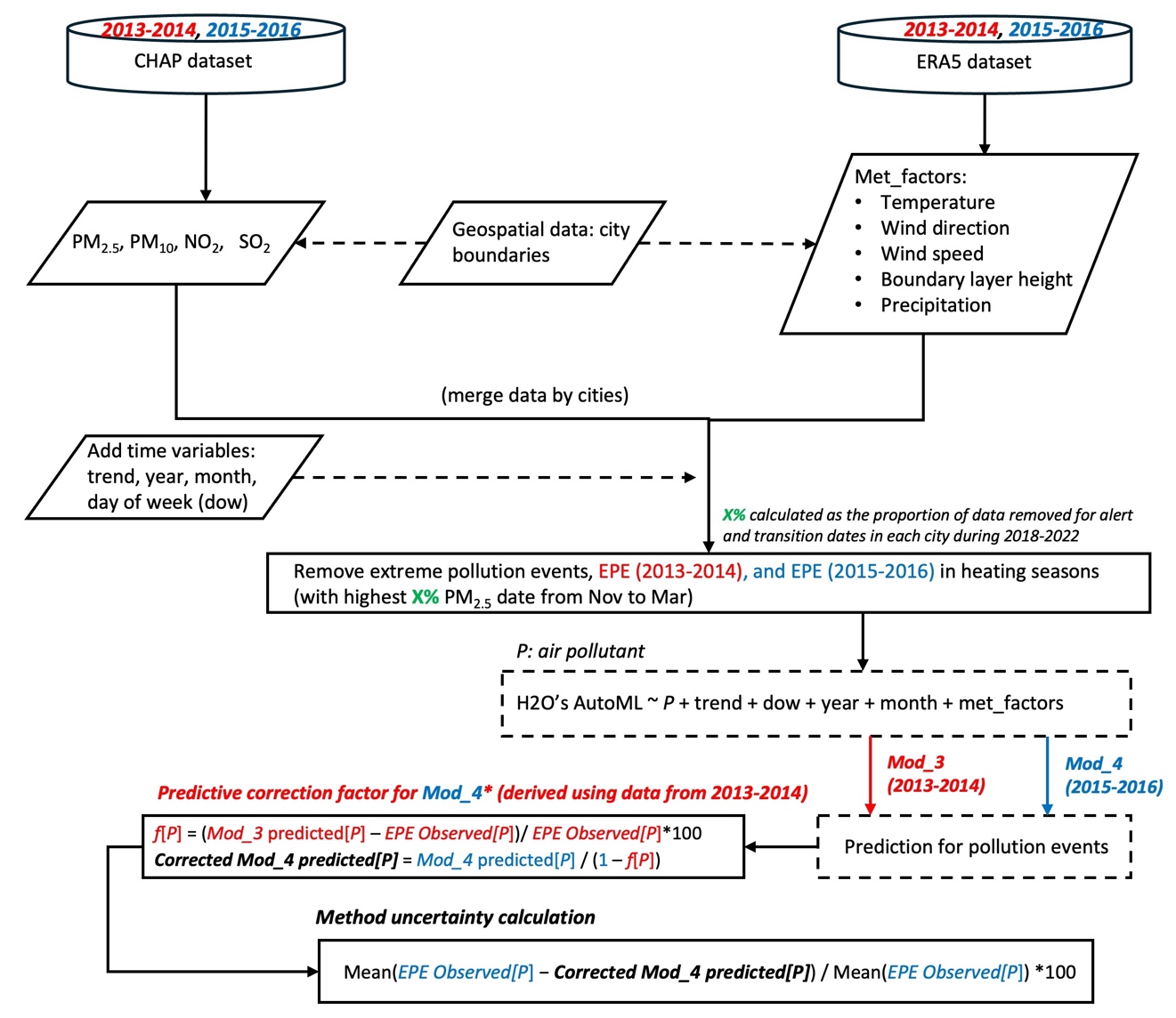
**

**Fig. S10. Methodological workflow for evaluating base model correction approach.** Air pollutants were sourced from ChinaHighAirPollutants (CHAP), and meteorological data were obtained from the ERA5 reanalysis by ECMWF for 2013-2016. Geospatial information was used to align data with target cities. The pollution alert differential was calculated as the difference between observed pollution levels on alert dates and predicted pollution levels after correction. To account for weather variability, pollution predictions were generated using a gradient boosting model from H2O’s AutoML platform.

**
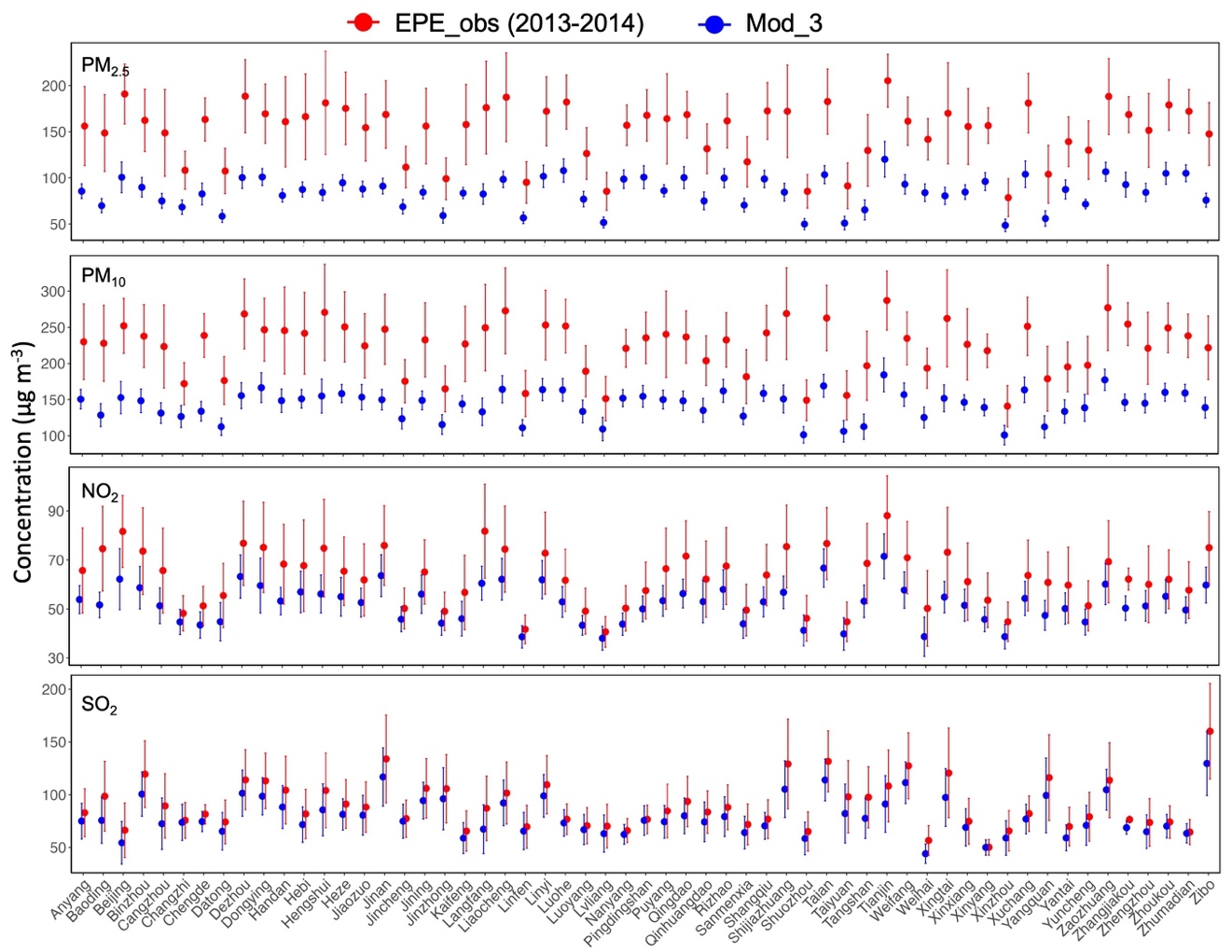
**

Fig. S11. Concentrations of observed and modeled pollutants across different cities between 2013 and 2014. The base model (mod_3) underestimated pollutant concentrations when extreme pollution episodes (EPE) in winter were excluded. The differences between observed and modeled values were used to derive correction factors for the base model (mod_4, Fig. S8) for each city.

**
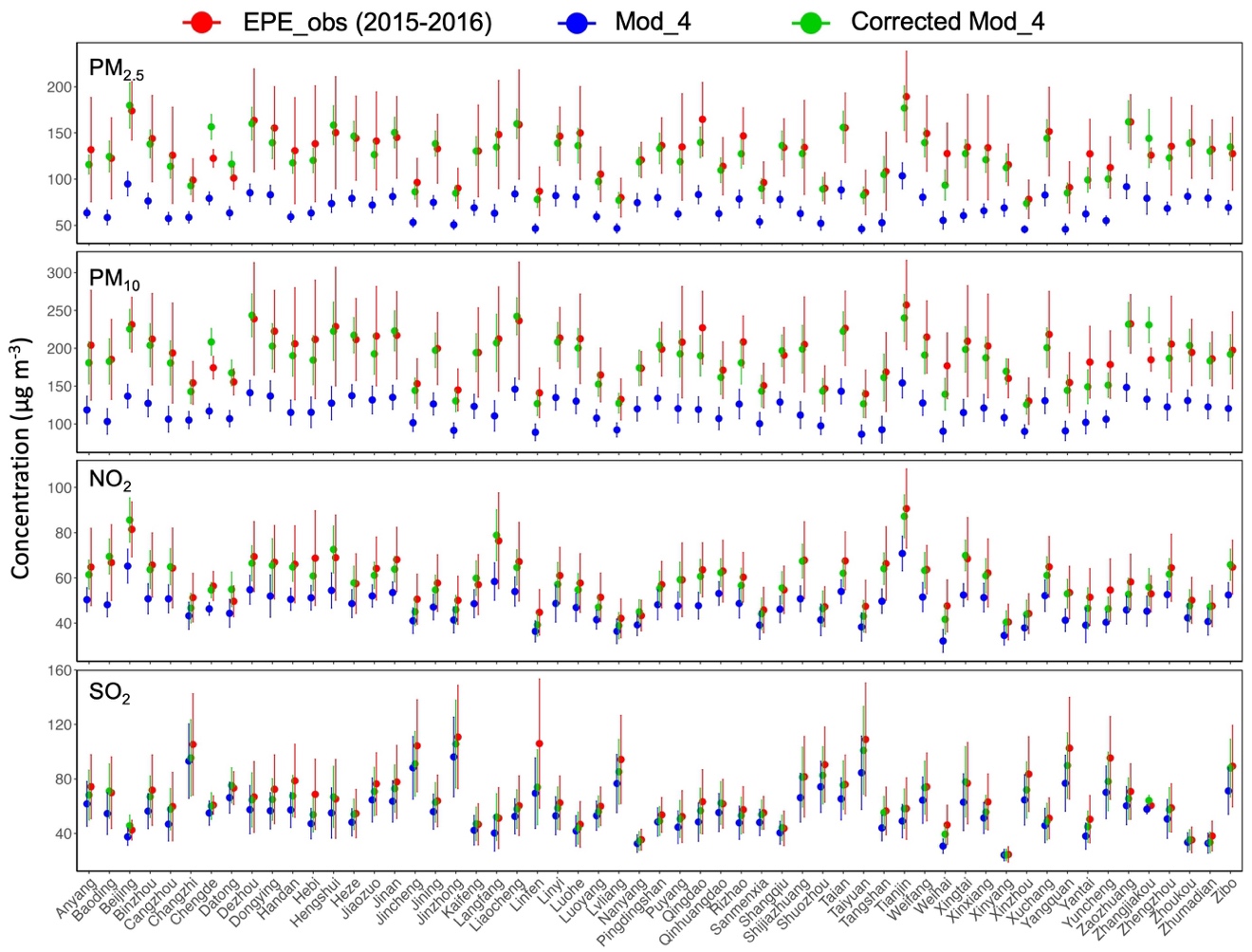
**

Fig. S12. Concentrations of observed and modeled pollutants across different cities between 2015 and 2016. The base model (mod_4) underestimated pollutant concentrations when extreme pollution episodes (EPE) in winter were excluded. The corrected base models significantly improved their performance in predicting pollutant concentrations during EPE.


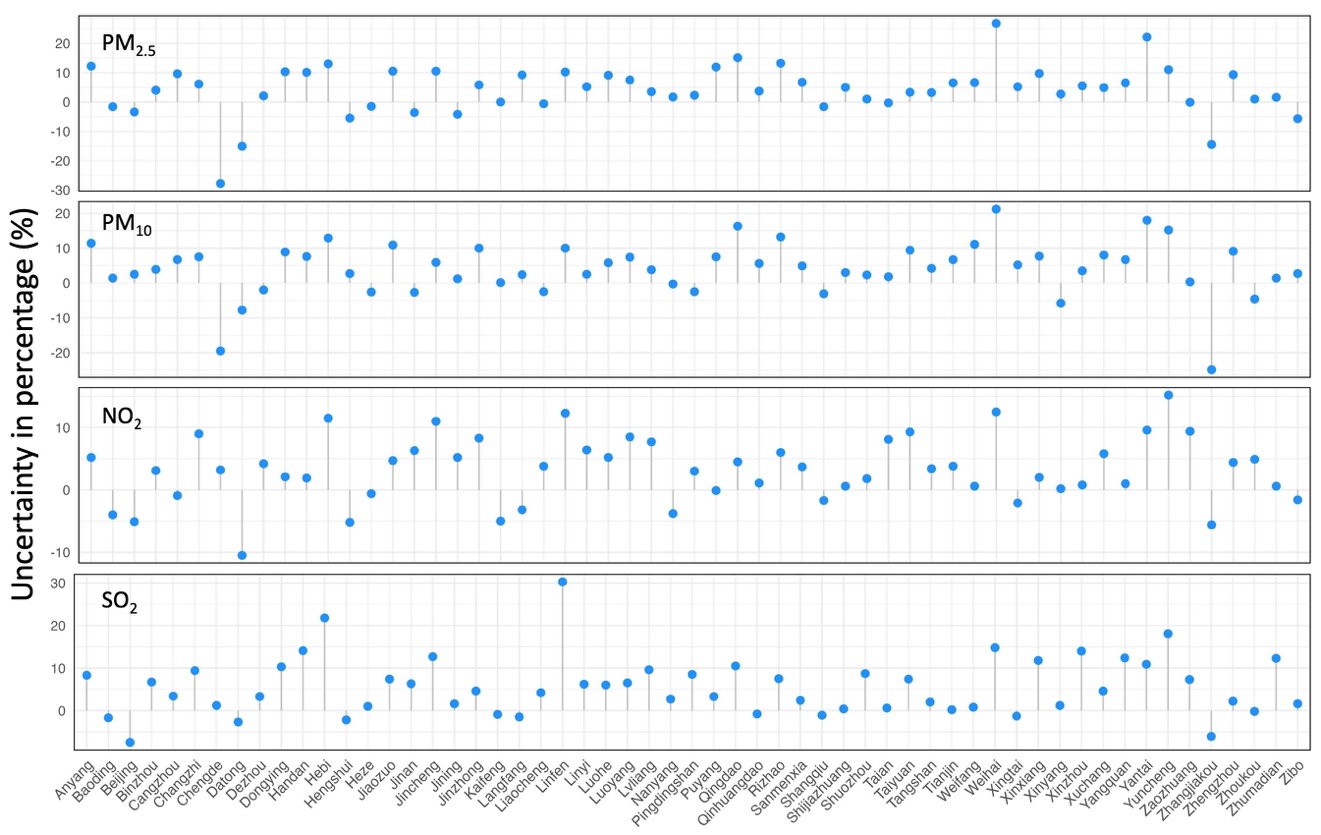


Fig. S13. Uncertainties in percentage between observed and modeled (after correction) concentrations during extreme pollution episodes (EPE).


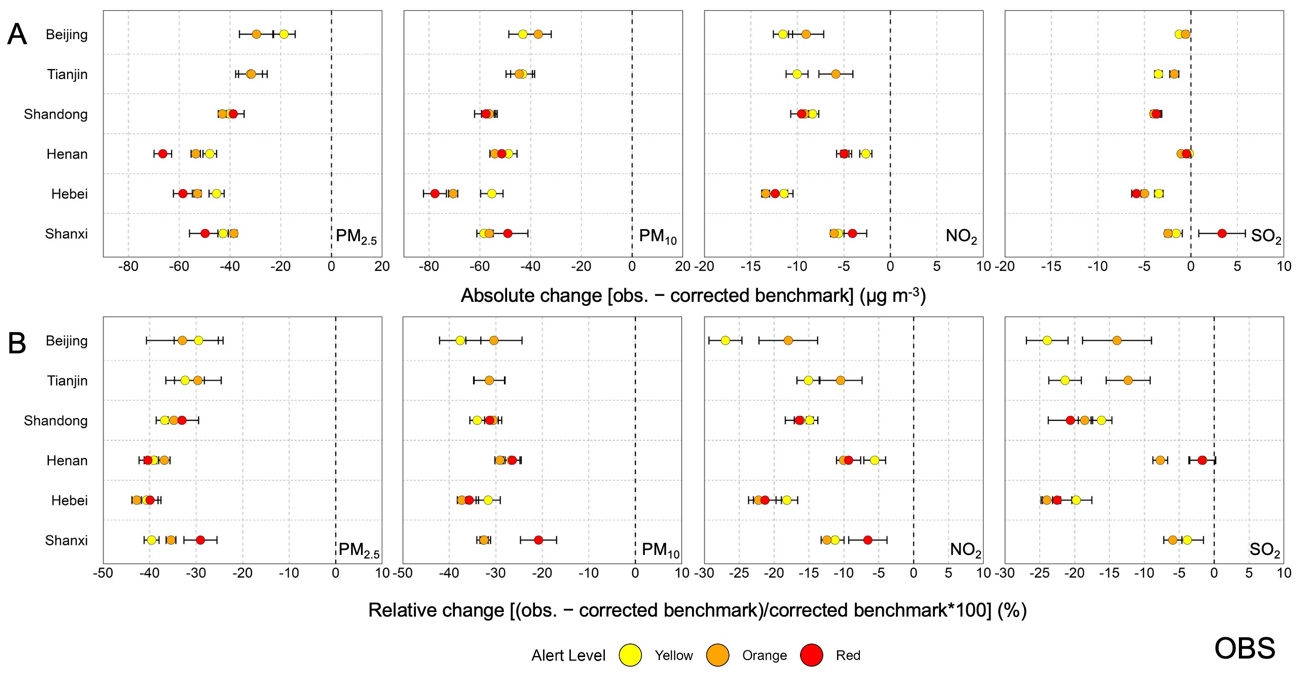


**Fig. S14. Province-Specific Pollution Anomalies in Ground-Level Air Pollution Alerts (model trained using data from air quality monitoring stations). (A)** Concentration differences between observed ambient pollutant concentrations (obs.) and corrected predictions from a base model. **(B)** Percentage differences between observed ambient pollutant concentrations (obs.) and corrected predictions from a base model. Points show cross-city mean values for each alert tier (color-coded), and error bars represent standard errors (SE) calculated from variability among city-level estimates.


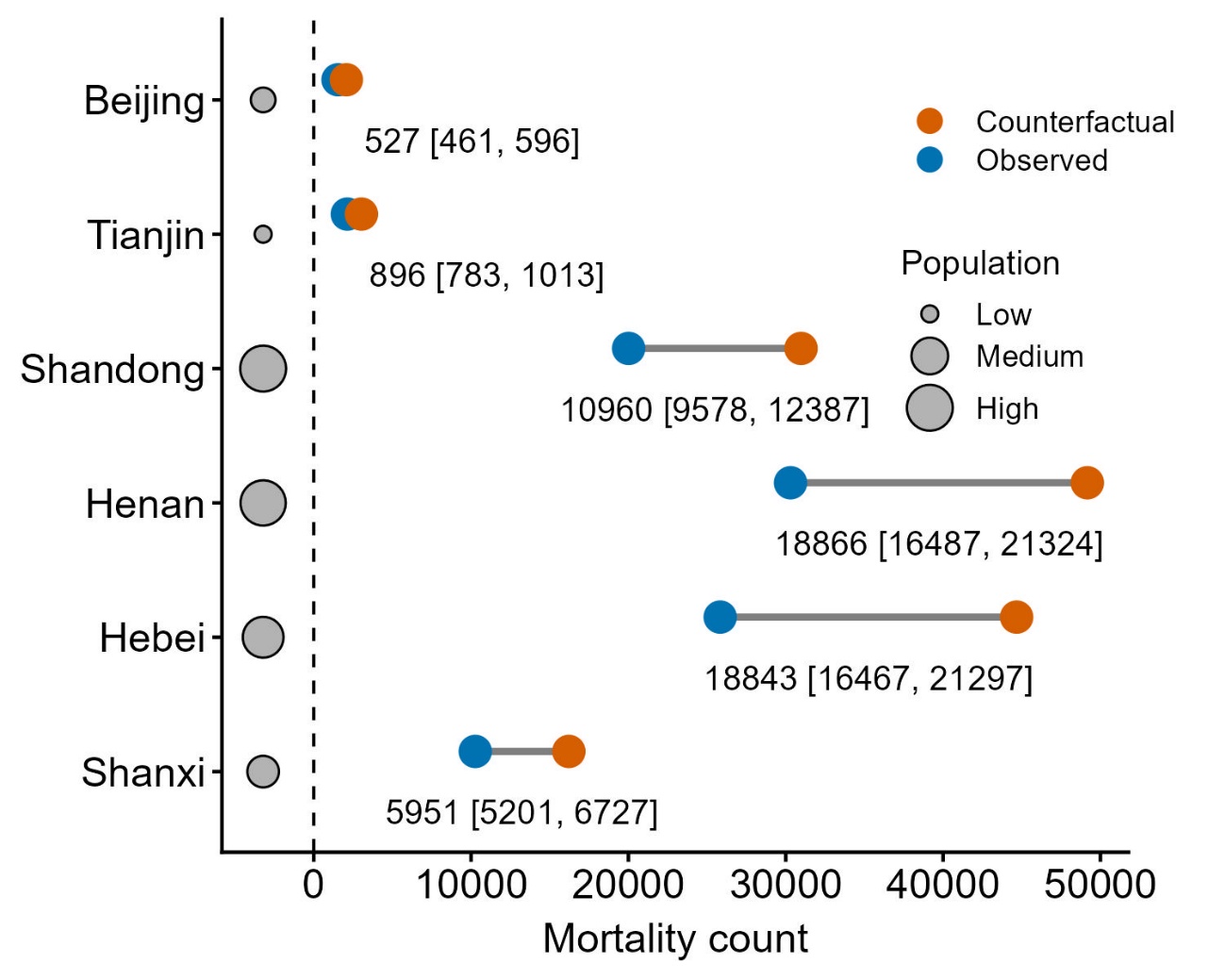


**Fig. S15. Province-Specific Short-term PM_2.5_-Attributable Mortality Under Observed Versus Counterfactual Pollution Alert Scenarios (model trained using data from air quality monitoring stations).** The observed scenario with implemented pollution alerts (blue dots) and a counterfactual scenario without alerts (orange dots) was compared. The differences between these scenarios, indicated by text below each segment with bracketed ranges “[.]” (which reflect different assumptions about baseline mortality rates), represents our estimate of the number of premature deaths avoided between 2018 and 2022 due to the pollution alerts.


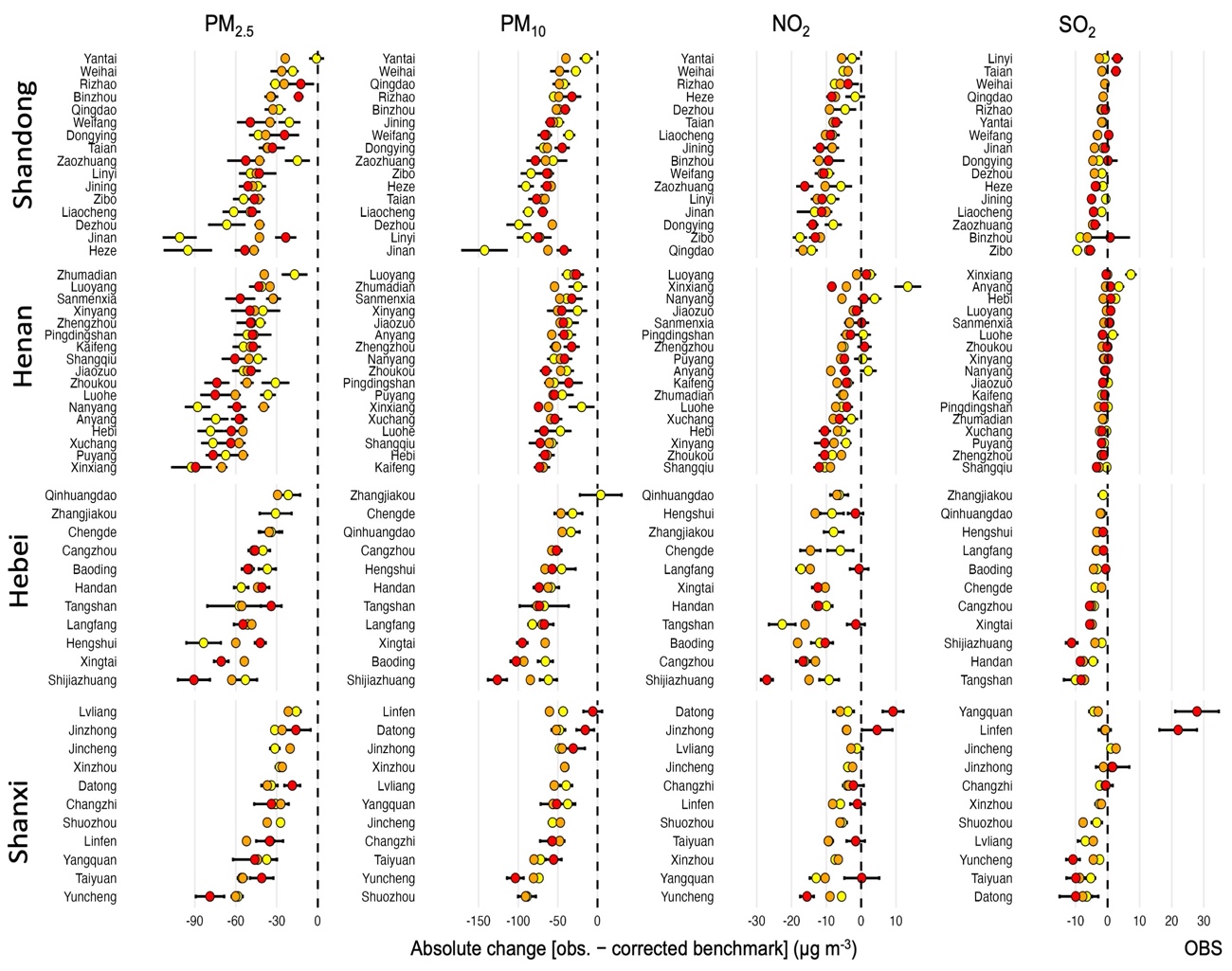


**Fig. S16. City-specific anomalies in ground-level air pollution during pollution alert periods (model trained using data from air quality monitoring stations).** Differences between observed ambient pollutant concentrations (obs.) and predictions from a base model (trained on 2018-2022 data, excluding alert and transition dates, after correction) during alert periods are shown for 57 Chinese cities with standard errors (SE, error bars). Both absolute (A) and relative (B) changes are presented. Cities are grouped by province, and an asterisk (*) denotes municipalities directly under the central government (Beijing and Tianjin). Points show cross-city mean values for each alert tier (color-coded), and error bars represent standard errors (SE) calculated from variability among city-level estimates.

Table S1. Timeline of air pollution alerts by city and alert level. This table lists the start and end dates of pollution alerts for 57 cities across Chinese provinces and municipalities, categorized by alert levels (yellow, orange, and red). For each city, the timeline captures the specific periods when pollution alerts were triggered, indicating the duration and frequency of alerts at different severity levels. The population (2018) and GDP (2021) of each city are also provided.

- *See attached Table (Table_S1)*.

Table S2. Gradient Boosting Model (GBM) performance by city for predicting air pollutant concentrations (Mod_1). GBM models were trained on daily meteorological factors, trend, year, month, and day of week (dow) from 2018 to 2020 for each pollutant, excluding alert (all levels) and transition dates. Model performance was evaluated using the revised index of agreement (IOA), correlation coefficient (*r*), and root mean square error (RMSE). An IOA above 0.5 signifies good model performance, and the average *r* indicates strong predictive accuracy across models.

| **City** | **PM_2.5_** | | | **PM_10_** | | | **NO_2_** | | | **SO_2_** | | |
| --- | --- | --- | --- | --- | --- | --- | --- | --- | --- | --- | --- | --- |
|  | ***r*** | **RMSE** | **IOA** | ***r*** | **RMSE** | **IOA** | ***r*** | **RMSE** | **IOA** | ***r*** | **RMSE** | **IOA** |
| Anyang | 0.82 | 18.43 | 0.73 | 0.79 | 29.74 | 0.70 | 0.92 | 5.39 | 0.82 | 0.97 | 2.83 | 0.87 |
| Baoding | 0.87 | 12.98 | 0.74 | 0.59 | 47.32 | 0.72 | 0.94 | 5.09 | 0.84 | 0.97 | 2.78 | 0.89 |
| Beijing | 0.73 | 16.14 | 0.69 | 0.73 | 23.25 | 0.68 | 0.93 | 4.88 | 0.83 | 0.95 | 1.58 | 0.85 |
| Binzhou | 0.77 | 16.84 | 0.69 | 0.67 | 37.92 | 0.70 | 0.92 | 5.68 | 0.81 | 0.95 | 3.50 | 0.85 |
| Cangzhou | 0.83 | 16.50 | 0.72 | 0.72 | 34.43 | 0.70 | 0.94 | 5.41 | 0.83 | 0.93 | 3.33 | 0.84 |
| Changzhi | 0.82 | 11.23 | 0.73 | 0.83 | 20.42 | 0.75 | 0.95 | 3.28 | 0.84 | 0.98 | 3.35 | 0.90 |
| Chengde | 0.85 | 8.19 | 0.75 | 0.81 | 18.32 | 0.72 | 0.91 | 3.44 | 0.82 | 0.97 | 1.37 | 0.88 |
| Datong | 0.71 | 13.79 | 0.70 | 0.75 | 27.90 | 0.68 | 0.92 | 3.66 | 0.82 | 0.96 | 2.84 | 0.86 |
| Dezhou | 0.84 | 16.62 | 0.72 | 0.75 | 29.50 | 0.69 | 0.93 | 5.42 | 0.83 | 0.94 | 2.99 | 0.82 |
| Dongying | 0.81 | 15.51 | 0.73 | 0.74 | 34.85 | 0.73 | 0.91 | 6.30 | 0.81 | 0.94 | 3.49 | 0.84 |
| Handan | 0.80 | 18.61 | 0.71 | 0.67 | 39.88 | 0.69 | 0.92 | 5.32 | 0.82 | 0.97 | 3.11 | 0.88 |
| Hebi | 0.80 | 23.02 | 0.69 | 0.69 | 42.10 | 0.68 | 0.90 | 6.81 | 0.80 | 0.92 | 3.71 | 0.80 |
| Hengshui | 0.78 | 18.35 | 0.70 | 0.74 | 31.28 | 0.69 | 0.93 | 5.26 | 0.82 | 0.89 | 3.37 | 0.79 |
| Heze | 0.83 | 16.78 | 0.73 | 0.71 | 35.99 | 0.69 | 0.91 | 5.26 | 0.81 | 0.91 | 2.64 | 0.79 |
| Jiaozuo | 0.79 | 19.07 | 0.73 | 0.75 | 30.27 | 0.68 | 0.91 | 5.50 | 0.81 | 0.96 | 2.77 | 0.87 |
| Jinan | 0.79 | 17.42 | 0.69 | 0.74 | 32.11 | 0.69 | 0.92 | 5.00 | 0.82 | 0.96 | 2.60 | 0.87 |
| Jincheng | 0.80 | 12.12 | 0.72 | 0.71 | 27.79 | 0.70 | 0.92 | 3.84 | 0.82 | 0.96 | 4.57 | 0.88 |
| Jining | 0.82 | 14.59 | 0.73 | 0.78 | 25.22 | 0.71 | 0.93 | 4.60 | 0.82 | 0.95 | 2.45 | 0.86 |
| Jinzhong | 0.77 | 12.51 | 0.72 | 0.67 | 30.88 | 0.70 | 0.95 | 3.34 | 0.85 | 0.98 | 4.25 | 0.90 |
| Kaifeng | 0.82 | 20.07 | 0.73 | 0.67 | 39.26 | 0.67 | 0.91 | 5.73 | 0.80 | 0.92 | 2.62 | 0.81 |
| Langfang | 0.82 | 14.80 | 0.71 | 0.72 | 32.90 | 0.71 | 0.93 | 5.68 | 0.83 | 0.92 | 2.72 | 0.81 |
| Liaocheng | 0.75 | 20.35 | 0.70 | 0.59 | 39.79 | 0.65 | 0.90 | 5.97 | 0.80 | 0.91 | 3.12 | 0.80 |
| Linfen | 0.85 | 12.08 | 0.74 | 0.76 | 35.26 | 0.72 | 0.95 | 3.19 | 0.84 | 0.99 | 4.01 | 0.93 |
| Linyi | 0.85 | 16.32 | 0.74 | 0.76 | 33.63 | 0.73 | 0.94 | 4.70 | 0.84 | 0.93 | 2.78 | 0.84 |
| Luohe | 0.83 | 20.89 | 0.76 | 0.65 | 50.22 | 0.71 | 0.91 | 5.61 | 0.80 | 0.88 | 2.29 | 0.78 |
| Luoyang | 0.83 | 12.78 | 0.75 | 0.79 | 25.08 | 0.74 | 0.94 | 3.62 | 0.84 | 0.96 | 2.18 | 0.89 |
| Lvliang | 0.78 | 11.47 | 0.72 | 0.66 | 32.62 | 0.70 | 0.95 | 2.95 | 0.86 | 0.96 | 4.69 | 0.89 |
| Nanyang | 0.86 | 14.94 | 0.77 | 0.78 | 27.52 | 0.75 | 0.96 | 3.16 | 0.87 | 0.95 | 1.28 | 0.85 |
| Pingdingshan | 0.85 | 16.80 | 0.77 | 0.74 | 32.49 | 0.71 | 0.93 | 4.16 | 0.83 | 0.96 | 1.91 | 0.86 |
| Puyang | 0.77 | 23.87 | 0.70 | 0.78 | 30.01 | 0.70 | 0.90 | 6.10 | 0.80 | 0.88 | 3.20 | 0.77 |
| Qingdao | 0.82 | 15.41 | 0.74 | 0.74 | 30.09 | 0.71 | 0.91 | 5.81 | 0.82 | 0.91 | 2.51 | 0.81 |
| Qinhuangdao | 0.81 | 12.58 | 0.72 | 0.78 | 23.97 | 0.74 | 0.94 | 4.44 | 0.84 | 0.98 | 2.23 | 0.90 |
| Rizhao | 0.85 | 13.79 | 0.75 | 0.77 | 28.79 | 0.73 | 0.92 | 5.02 | 0.82 | 0.93 | 2.28 | 0.83 |
| Sanmenxia | 0.81 | 12.59 | 0.73 | 0.73 | 33.28 | 0.74 | 0.94 | 3.20 | 0.85 | 0.97 | 1.80 | 0.89 |
| Shangqiu | 0.84 | 19.42 | 0.76 | 0.81 | 27.29 | 0.73 | 0.93 | 5.03 | 0.83 | 0.92 | 1.93 | 0.79 |
| Shijiazhuang | 0.80 | 18.99 | 0.72 | 0.65 | 46.16 | 0.71 | 0.94 | 4.69 | 0.83 | 0.98 | 3.29 | 0.89 |
| Shuozhou | 0.71 | 13.69 | 0.73 | 0.63 | 38.19 | 0.66 | 0.93 | 3.37 | 0.83 | 0.97 | 3.72 | 0.87 |
| Taian | 0.84 | 16.46 | 0.74 | 0.76 | 35.26 | 0.71 | 0.94 | 4.70 | 0.84 | 0.95 | 2.83 | 0.86 |
| Taiyuan | 0.80 | 13.37 | 0.72 | 0.54 | 49.83 | 0.68 | 0.93 | 3.91 | 0.83 | 0.98 | 4.04 | 0.89 |
| Tangshan | 0.84 | 13.28 | 0.72 | 0.72 | 31.39 | 0.72 | 0.93 | 5.56 | 0.83 | 0.97 | 2.51 | 0.89 |
| Tianjin | 0.83 | 17.32 | 0.72 | 0.66 | 35.96 | 0.67 | 0.92 | 6.80 | 0.82 | 0.91 | 2.65 | 0.79 |
| Weifang | 0.82 | 16.43 | 0.73 | 0.76 | 32.41 | 0.73 | 0.93 | 5.22 | 0.83 | 0.96 | 2.66 | 0.86 |
| Weihai | 0.79 | 11.31 | 0.71 | 0.73 | 22.15 | 0.69 | 0.87 | 5.08 | 0.78 | 0.90 | 1.59 | 0.80 |
| Xingtai | 0.79 | 21.56 | 0.71 | 0.79 | 29.80 | 0.72 | 0.94 | 5.34 | 0.83 | 0.97 | 3.16 | 0.87 |
| Xinxiang | 0.82 | 19.20 | 0.71 | 0.76 | 29.74 | 0.70 | 0.91 | 5.76 | 0.81 | 0.95 | 2.86 | 0.84 |
| Xinyang | 0.83 | 17.34 | 0.76 | 0.78 | 23.20 | 0.72 | 0.93 | 3.68 | 0.84 | 0.92 | 1.25 | 0.81 |
| Xinzhou | 0.80 | 10.15 | 0.72 | 0.71 | 36.94 | 0.71 | 0.96 | 2.78 | 0.87 | 0.98 | 3.09 | 0.90 |
| Xuchang | 0.86 | 18.29 | 0.77 | 0.67 | 43.53 | 0.70 | 0.91 | 5.64 | 0.81 | 0.94 | 2.62 | 0.83 |
| Yangquan | 0.82 | 13.00 | 0.73 | 0.69 | 31.28 | 0.70 | 0.91 | 4.68 | 0.81 | 0.96 | 5.10 | 0.86 |
| Yantai | 0.80 | 12.40 | 0.73 | 0.80 | 23.21 | 0.74 | 0.89 | 5.05 | 0.79 | 0.92 | 1.99 | 0.81 |
| Yuncheng | 0.83 | 15.58 | 0.74 | 0.62 | 50.64 | 0.70 | 0.94 | 3.92 | 0.82 | 0.98 | 3.97 | 0.91 |
| Zaozhuang | 0.84 | 18.30 | 0.74 | 0.72 | 40.31 | 0.72 | 0.91 | 5.39 | 0.81 | 0.94 | 3.26 | 0.84 |
| Zhangjiakou | 0.74 | 11.72 | 0.72 | 0.61 | 29.60 | 0.65 | 0.93 | 3.07 | 0.83 | 0.96 | 1.52 | 0.87 |
| Zhengzhou | 0.78 | 19.69 | 0.72 | 0.72 | 32.38 | 0.67 | 0.92 | 5.21 | 0.82 | 0.95 | 2.55 | 0.85 |
| Zhoukou | 0.83 | 20.35 | 0.75 | 0.73 | 33.89 | 0.70 | 0.89 | 5.43 | 0.80 | 0.92 | 1.92 | 0.81 |
| Zhumadian | 0.84 | 17.38 | 0.77 | 0.78 | 27.83 | 0.72 | 0.94 | 4.31 | 0.85 | 0.93 | 1.58 | 0.82 |
| Zibo | 0.73 | 16.31 | 0.67 | 0.77 | 27.10 | 0.70 | 0.91 | 5.42 | 0.80 | 0.96 | 3.45 | 0.87 |
| **Average** | **0.81** | **15.95** | **0.73** | **0.72** | **32.84** | **0.70** | **0.92** | **4.79** | **0.82** | **0.95** | **2.82** | **0.85** |

Table S3. City-specific number of excluded days, along with average PM_2.5_ levels and corresponding thresholds during the heating seasons (2013–2016), used for deriving correction factors.

| City | 2018-2022 | | 2013-2016 | | |
| --- | --- | --- | --- | --- | --- |
|  | No. of excluded days | average excluded PM_2.5_ level (µg m^-3^) | No. of excluded days | PM_2.5_ threshold (µg m^-3^) | average excluded PM_2.5_ level (µg m^-3^) |
| Beijing | 100 | 80.7 | 80 | 148.4 | 183.3 |
| Tianjin | 164 | 95.0 | 131 | 156.1 | 201.2 |
| Shijiazhuang | 536 | 77.9 | 429 | 89.2 | 154.3 |
| Tangshan | 517 | 63.7 | 414 | 66.4 | 119.5 |
| Qinhuangdao | 220 | 53.4 | 176 | 90.7 | 124.2 |
| Handan | 573 | 89.9 | 458 | 86.9 | 146.8 |
| Baoding | 540 | 69.3 | 432 | 80.0 | 135.9 |
| Chengde | 19 | 62.1 | 15 | 122.9 | 146.4 |
| Cangzhou | 568 | 77.3 | 454 | 79.8 | 137.8 |
| Langfang | 461 | 75.4 | 369 | 93.9 | 163.0 |
| Hengshui | 472 | 87.1 | 378 | 101.8 | 166.6 |
| Zhangjiakou | 10 | 80.5 | 8 | 120.8 | 149.9 |
| Taiyuan | 413 | 59.6 | 330 | 59.5 | 88.5 |
| Datong | 75 | 55.3 | 60 | 85.4 | 104.3 |
| Yangquan | 406 | 63.9 | 325 | 64.0 | 97.9 |
| Changzhi | 365 | 64.7 | 292 | 76.9 | 104.1 |
| Jincheng | 445 | 68.9 | 356 | 74.0 | 105.1 |
| Shuozhou | 174 | 46.1 | 139 | 66.2 | 88.0 |
| Jinzhong | 384 | 64.3 | 307 | 68.6 | 95.0 |
| Yuncheng | 457 | 80.7 | 366 | 83.6 | 122.1 |
| Xinzhou | 328 | 49.0 | 262 | 54.7 | 78.4 |
| Linfen | 557 | 65.6 | 446 | 59.3 | 91.4 |
| Lvliang | 385 | 51.9 | 308 | 59.1 | 82.8 |
| Jinan | 290 | 91.4 | 232 | 112.0 | 157.9 |
| Qingdao | 78 | 84.7 | 62 | 130.2 | 167.8 |
| Zibo | 351 | 84.2 | 281 | 95.8 | 138.4 |
| Zaozhuang | 184 | 79.6 | 147 | 135.3 | 176.5 |
| Dongying | 180 | 76.1 | 144 | 122.2 | 163.9 |
| Yantai | 113 | 63.3 | 90 | 103.0 | 135.7 |
| Weifang | 162 | 72.6 | 130 | 120.0 | 157.2 |
| Jining | 416 | 90.8 | 333 | 100.3 | 145.0 |
| Taian | 216 | 78.7 | 173 | 127.0 | 170.8 |
| Weihai | 49 | 49.8 | 39 | 109.1 | 137.7 |
| Rizhao | 138 | 68.0 | 110 | 121.6 | 155.2 |
| Linyi | 203 | 73.9 | 162 | 120.6 | 160.1 |
| Dezhou | 270 | 99.0 | 216 | 127.0 | 178.0 |
| Liaocheng | 339 | 102.1 | 271 | 121.6 | 174.8 |
| Binzhou | 285 | 91.9 | 228 | 111.0 | 154.4 |
| Heze | 338 | 103.6 | 270 | 113.2 | 160.9 |
| Zhengzhou | 500 | 90.6 | 400 | 93.2 | 144.2 |
| Kaifeng | 489 | 94.9 | 391 | 95.0 | 145.0 |
| Luoyang | 422 | 72.5 | 338 | 81.6 | 117.1 |
| Pingdingshan | 261 | 76.4 | 209 | 117.0 | 154.3 |
| Anyang | 525 | 88.6 | 420 | 93.1 | 145.2 |
| Hebi | 503 | 92.5 | 402 | 98.1 | 153.9 |
| Xinxiang | 527 | 85.9 | 422 | 92.8 | 145.7 |
| Jiaozuo | 477 | 89.1 | 382 | 98.8 | 148.5 |
| Puyang | 521 | 94.6 | 417 | 96.3 | 150.7 |
| Xuchang | 279 | 82.0 | 223 | 123.9 | 168.3 |
| Luohe | 256 | 85.0 | 205 | 124.2 | 168.5 |
| Sanmenxia | 360 | 67.5 | 288 | 78.3 | 108.0 |
| Nanyang | 217 | 71.8 | 174 | 112.5 | 141.5 |
| Shangqiu | 244 | 82.3 | 195 | 117.5 | 155.7 |
| Xinyang | 161 | 66.4 | 129 | 108.6 | 141.1 |
| Zhoukou | 216 | 82.7 | 173 | 124.2 | 163.2 |
| Zhumadian | 203 | 81.1 | 162 | 120.2 | 156.3 |
| Xingtai | 584 | 86.3 | 467 | 86.6 | 153.3 |

Table S4. Model performance statistics by city for air pollutants for model evaluation (Mod_2). GBM models were trained on daily meteorological factors, trend, year, month, and day of week (dow) from 2013 to 2016 for each pollutant, excluding extreme high fine particle (PM_2.5_) concentrations as in Fig. S5 (Mod_2). Model performance was evaluated using the revised index of agreement (IOA), correlation coefficient (*r*), and root mean square error (RMSE). An IOA above 0.5 signifies good model performance, and the average *r* indicates strong predictive accuracy across models.

| **City** | **PM_2.5_** | | | **PM_10_** | | | **NO_2_** | | | **SO_2_** | | |
| --- | --- | --- | --- | --- | --- | --- | --- | --- | --- | --- | --- | --- |
|  | ***r*** | **RMSE** | **IOA** | ***r*** | **RMSE** | **IOA** | ***r*** | **RMSE** | **IOA** | ***r*** | **RMSE** | **IOA** |
| Anyang | 0.67 | 15.18 | 0.64 | 0.78 | 21.66 | 0.70 | 0.84 | 5.09 | 0.75 | 0.94 | 6.19 | 0.82 |
| Baoding | 0.68 | 16.27 | 0.66 | 0.75 | 23.24 | 0.70 | 0.86 | 4.69 | 0.75 | 0.94 | 5.70 | 0.84 |
| Beijing | 0.76 | 20.69 | 0.68 | 0.74 | 29.66 | 0.68 | 0.91 | 6.85 | 0.81 | 0.97 | 4.37 | 0.89 |
| Binzhou | 0.63 | 17.85 | 0.62 | 0.68 | 27.71 | 0.64 | 0.87 | 5.87 | 0.76 | 0.93 | 9.52 | 0.81 |
| Cangzhou | 0.63 | 18.27 | 0.63 | 0.73 | 23.84 | 0.67 | 0.82 | 5.37 | 0.72 | 0.93 | 5.63 | 0.82 |
| Changzhi | 0.69 | 10.82 | 0.65 | 0.77 | 19.42 | 0.68 | 0.90 | 3.45 | 0.78 | 0.96 | 6.71 | 0.86 |
| Chengde | 0.82 | 10.66 | 0.74 | 0.83 | 17.74 | 0.72 | 0.91 | 4.11 | 0.81 | 0.98 | 3.45 | 0.92 |
| Datong | 0.78 | 9.57 | 0.71 | 0.74 | 20.67 | 0.70 | 0.92 | 3.92 | 0.82 | 0.97 | 4.63 | 0.90 |
| Dezhou | 0.63 | 19.34 | 0.63 | 0.74 | 26.94 | 0.67 | 0.89 | 5.49 | 0.79 | 0.93 | 7.78 | 0.82 |
| Dongying | 0.75 | 16.27 | 0.68 | 0.76 | 28.18 | 0.68 | 0.89 | 6.11 | 0.78 | 0.90 | 9.34 | 0.78 |
| Handan | 0.59 | 16.62 | 0.60 | 0.76 | 24.27 | 0.68 | 0.80 | 5.26 | 0.70 | 0.94 | 5.95 | 0.83 |
| Hebi | 0.63 | 17.20 | 0.64 | 0.72 | 26.35 | 0.67 | 0.80 | 6.65 | 0.71 | 0.90 | 7.40 | 0.78 |
| Hengshui | 0.59 | 19.77 | 0.60 | 0.71 | 30.23 | 0.65 | 0.89 | 5.09 | 0.78 | 0.95 | 6.74 | 0.84 |
| Heze | 0.69 | 16.44 | 0.66 | 0.77 | 25.38 | 0.70 | 0.88 | 5.24 | 0.78 | 0.94 | 6.42 | 0.83 |
| Jiaozuo | 0.66 | 16.83 | 0.66 | 0.73 | 23.50 | 0.66 | 0.88 | 4.96 | 0.78 | 0.94 | 8.61 | 0.83 |
| Jinan | 0.63 | 16.67 | 0.61 | 0.66 | 26.51 | 0.64 | 0.86 | 5.85 | 0.76 | 0.93 | 9.14 | 0.83 |
| Jincheng | 0.74 | 9.72 | 0.67 | 0.80 | 16.41 | 0.71 | 0.89 | 3.84 | 0.78 | 0.92 | 9.57 | 0.83 |
| Jining | 0.70 | 13.87 | 0.66 | 0.71 | 25.56 | 0.68 | 0.91 | 4.46 | 0.80 | 0.93 | 6.82 | 0.83 |
| Jinzhong | 0.66 | 10.93 | 0.63 | 0.77 | 17.43 | 0.68 | 0.91 | 3.25 | 0.79 | 0.96 | 8.65 | 0.86 |
| Kaifeng | 0.64 | 15.93 | 0.64 | 0.76 | 24.07 | 0.69 | 0.86 | 5.22 | 0.76 | 0.93 | 5.61 | 0.82 |
| Langfang | 0.68 | 19.40 | 0.64 | 0.71 | 28.09 | 0.67 | 0.85 | 5.95 | 0.75 | 0.95 | 5.37 | 0.84 |
| Liaocheng | 0.63 | 18.97 | 0.63 | 0.68 | 29.45 | 0.64 | 0.86 | 6.21 | 0.76 | 0.94 | 8.37 | 0.83 |
| Linfen | 0.70 | 9.63 | 0.64 | 0.77 | 16.00 | 0.71 | 0.87 | 2.89 | 0.77 | 0.93 | 6.73 | 0.80 |
| Linyi | 0.73 | 16.86 | 0.66 | 0.78 | 26.16 | 0.70 | 0.89 | 5.35 | 0.79 | 0.94 | 7.86 | 0.84 |
| Luohe | 0.74 | 17.25 | 0.68 | 0.75 | 25.97 | 0.70 | 0.87 | 5.09 | 0.77 | 0.91 | 6.01 | 0.81 |
| Luoyang | 0.70 | 10.82 | 0.65 | 0.75 | 19.15 | 0.69 | 0.91 | 3.53 | 0.81 | 0.95 | 4.90 | 0.85 |
| Lvliang | 0.65 | 9.23 | 0.62 | 0.75 | 17.57 | 0.68 | 0.93 | 2.40 | 0.81 | 0.96 | 5.56 | 0.87 |
| Nanyang | 0.75 | 14.47 | 0.69 | 0.83 | 18.67 | 0.75 | 0.92 | 2.83 | 0.82 | 0.96 | 3.56 | 0.86 |
| Pingdingshan | 0.77 | 15.79 | 0.70 | 0.75 | 23.13 | 0.69 | 0.86 | 4.49 | 0.77 | 0.94 | 5.14 | 0.83 |
| Puyang | 0.63 | 18.21 | 0.62 | 0.75 | 23.69 | 0.69 | 0.87 | 4.96 | 0.76 | 0.92 | 6.83 | 0.80 |
| Qingdao | 0.76 | 16.31 | 0.69 | 0.72 | 26.55 | 0.68 | 0.85 | 6.26 | 0.76 | 0.91 | 7.09 | 0.82 |
| Qinhuangdao | 0.77 | 12.00 | 0.70 | 0.76 | 20.67 | 0.69 | 0.87 | 5.65 | 0.76 | 0.95 | 5.79 | 0.85 |
| Rizhao | 0.75 | 16.21 | 0.68 | 0.76 | 26.18 | 0.69 | 0.88 | 5.46 | 0.78 | 0.94 | 6.51 | 0.84 |
| Sanmenxia | 0.75 | 10.17 | 0.69 | 0.84 | 15.70 | 0.74 | 0.91 | 3.34 | 0.81 | 0.95 | 4.51 | 0.85 |
| Shangqiu | 0.73 | 16.22 | 0.67 | 0.76 | 23.70 | 0.69 | 0.89 | 4.95 | 0.78 | 0.94 | 4.71 | 0.84 |
| Shijiazhuang | 0.67 | 20.19 | 0.65 | 0.78 | 27.60 | 0.69 | 0.86 | 4.99 | 0.75 | 0.92 | 8.54 | 0.81 |
| Shuozhou | 0.72 | 8.68 | 0.69 | 0.74 | 18.42 | 0.69 | 0.93 | 3.17 | 0.82 | 0.98 | 4.43 | 0.89 |
| Taian | 0.65 | 18.48 | 0.63 | 0.78 | 25.28 | 0.69 | 0.88 | 5.72 | 0.78 | 0.94 | 7.92 | 0.85 |
| Taiyuan | 0.69 | 10.15 | 0.64 | 0.72 | 19.14 | 0.67 | 0.86 | 3.43 | 0.74 | 0.94 | 9.37 | 0.83 |
| Tangshan | 0.73 | 14.28 | 0.67 | 0.78 | 20.54 | 0.71 | 0.78 | 5.21 | 0.70 | 0.91 | 6.66 | 0.81 |
| Tianjin | 0.75 | 19.95 | 0.69 | 0.78 | 28.93 | 0.70 | 0.89 | 6.53 | 0.78 | 0.95 | 7.42 | 0.85 |
| Weifang | 0.65 | 16.95 | 0.62 | 0.69 | 28.95 | 0.65 | 0.87 | 5.53 | 0.76 | 0.94 | 8.35 | 0.84 |
| Weihai | 0.75 | 13.86 | 0.68 | 0.71 | 21.29 | 0.67 | 0.82 | 4.97 | 0.76 | 0.90 | 3.90 | 0.79 |
| Xingtai | 0.61 | 20.47 | 0.60 | 0.70 | 30.19 | 0.66 | 0.86 | 4.96 | 0.75 | 0.93 | 7.82 | 0.82 |
| Xinxiang | 0.70 | 15.44 | 0.66 | 0.75 | 23.31 | 0.67 | 0.86 | 4.92 | 0.74 | 0.94 | 5.76 | 0.83 |
| Xinyang | 0.78 | 13.88 | 0.71 | 0.75 | 22.66 | 0.72 | 0.92 | 3.30 | 0.83 | 0.96 | 2.99 | 0.87 |
| Xinzhou | 0.75 | 7.01 | 0.69 | 0.78 | 15.06 | 0.69 | 0.92 | 2.54 | 0.81 | 0.97 | 3.92 | 0.88 |
| Xuchang | 0.76 | 16.28 | 0.68 | 0.73 | 29.40 | 0.69 | 0.87 | 5.44 | 0.76 | 0.94 | 5.75 | 0.83 |
| Yangquan | 0.71 | 10.57 | 0.66 | 0.68 | 23.29 | 0.66 | 0.85 | 4.58 | 0.74 | 0.92 | 10.28 | 0.80 |
| Yantai | 0.73 | 13.76 | 0.68 | 0.67 | 24.84 | 0.66 | 0.84 | 5.28 | 0.75 | 0.91 | 5.18 | 0.80 |
| Yuncheng | 0.74 | 10.68 | 0.67 | 0.73 | 23.37 | 0.70 | 0.89 | 4.36 | 0.79 | 0.94 | 7.78 | 0.84 |
| Zaozhuang | 0.74 | 17.89 | 0.67 | 0.77 | 27.55 | 0.70 | 0.89 | 5.36 | 0.78 | 0.92 | 9.57 | 0.81 |
| Zhangjiakou | 0.83 | 10.49 | 0.74 | 0.82 | 18.35 | 0.73 | 0.93 | 3.53 | 0.84 | 0.98 | 3.55 | 0.91 |
| Zhengzhou | 0.68 | 15.31 | 0.64 | 0.72 | 25.80 | 0.67 | 0.88 | 4.84 | 0.77 | 0.95 | 5.79 | 0.85 |
| Zhoukou | 0.73 | 17.07 | 0.70 | 0.76 | 24.54 | 0.70 | 0.87 | 4.68 | 0.77 | 0.94 | 4.72 | 0.85 |
| Zhumadian | 0.79 | 14.77 | 0.72 | 0.76 | 25.95 | 0.70 | 0.91 | 3.66 | 0.80 | 0.92 | 5.11 | 0.83 |
| Zibo | 0.62 | 14.81 | 0.63 | 0.66 | 25.74 | 0.63 | 0.83 | 5.66 | 0.74 | 0.91 | 12.41 | 0.80 |
| **Average** | **0.70** | **14.94** | **0.66** | **0.75** | **23.68** | **0.69** | **0.88** | **4.79** | **0.77** | **0.94** | **6.57** | **0.84** |

Table S5. Median impact of pollution alerts on urban air quality. The values are the median differences between the pollutant concentrations observed and the counterfactual concentrations estimated for the same period had no alerts been issued. Negative values therefore signify that air quality improved because of the alerts, whereas positive values indicate a deterioration or no net benefit at the median point.

| **City** | **PM_2.5_ (μg m^-3^)** | | | **PM_10_ (μg m^-3^)** | | | **NO_2_ (μg m^-3^)** | | | **SO_2_ (μg m^-3^)** | | |
| --- | --- | --- | --- | --- | --- | --- | --- | --- | --- | --- | --- | --- |
|  | **Yellow** | **Orange** | **Red** | **Yellow** | **Orange** | **Red** | **Yellow** | **Orange** | **Red** | **Yellow** | **Orange** | **Red** |
| Anyang | -92.8 | -58.7 | -53.4 | -57.9 | -61.8 | -54.9 | -2.4 | -8.0 | -6.6 | 4.0 | -0.6 | 0.2 |
| Baoding | -34.6 | -46.4 | -53.8 | -41.8 | -67.7 | -68.2 | -9.3 | -15.2 | -3.2 | -1.4 | -4.1 | -0.2 |
| Beijing | -31.3 | -35.4 | NA | -42.6 | -39.5 | NA | -10.2 | -9.0 | NA | -3.0 | -1.8 | NA |
| Binzhou | -31.6 | -36.6 | -41.4 | -44.9 | -51.8 | -40.0 | -8.1 | -11.0 | -11.7 | -5.2 | -5.0 | 1.0 |
| Cangzhou | -42.0 | -47.9 | -41.8 | -61.4 | -60.3 | -67.1 | -13.0 | -11.2 | -13.1 | -3.0 | -4.0 | -4.6 |
| Changzhi | -30.2 | -27.4 | -54.2 | -47.3 | -45.8 | -65.0 | -4.8 | -4.0 | -6.1 | -2.7 | -1.9 | -1.6 |
| Chengde | -27.6 | -21.4 | NA | -11.2 | -37.3 | NA | -4.7 | -8.3 | NA | -1.8 | -3.4 | NA |
| Datong | -24.7 | -29.2 | -29.9 | -46.1 | -54.6 | -54.9 | -6.6 | -9.7 | -4.4 | -2.1 | -5.8 | -8.7 |
| Dezhou | -62.4 | -41.2 | NA | -98.1 | -69.5 | NA | -10.1 | -11.7 | NA | -2.4 | -3.6 | NA |
| Dongying | -55.5 | -34.5 | -23.7 | -71.4 | -56.1 | -49.2 | -12.5 | -14.5 | -11.9 | -2.4 | -3.1 | -2.0 |
| Handan | -40.1 | -46.2 | -37.6 | -49.3 | -65.6 | -69.2 | -9.0 | -12.3 | -8.2 | -2.3 | -5.3 | -5.7 |
| Hebi | -72.1 | -54.5 | -57.1 | -50.2 | -56.4 | -64.3 | -4.6 | -7.9 | -7.1 | 0.0 | -1.3 | -0.3 |
| Hengshui | -64.2 | -55.3 | -46.8 | -58.2 | -73.3 | -84.6 | -11.2 | -13.0 | -2.4 | -3.5 | -3.4 | -2.2 |
| Heze | -58.7 | -51.7 | -44.4 | -77.3 | -59.0 | -58.3 | -6.8 | -6.8 | -5.6 | -0.6 | -2.5 | -2.0 |
| Jiaozuo | -45.3 | -44.7 | -41.4 | -27.7 | -57.0 | -61.9 | -4.6 | -5.8 | -5.6 | 0.5 | -1.6 | -0.8 |
| Jinan | -126.7 | -43.7 | -32.0 | -131.1 | -57.6 | -41.5 | -9.0 | -10.7 | -10.3 | -2.7 | -3.1 | -0.5 |
| Jincheng | -27.9 | -25.3 | NA | -39.6 | -41.5 | NA | -1.7 | -2.8 | NA | -2.3 | -1.9 | NA |
| Jining | -51.2 | -46.8 | -58.1 | -50.2 | -57.3 | -50.9 | -9.3 | -9.3 | -8.5 | -2.0 | -3.9 | -3.2 |
| Jinzhong | -26.1 | -29.0 | -21.4 | -41.9 | -44.8 | -38.5 | -2.3 | -2.9 | 4.5 | -2.0 | -3.1 | -5.4 |
| Kaifeng | -51.9 | -47.1 | -47.0 | -68.8 | -59.0 | -70.2 | -10.4 | -7.7 | -7.2 | -2.2 | -0.9 | -1.0 |
| Langfang | -58.8 | -57.7 | -48.3 | -80.1 | -77.2 | -85.7 | -19.5 | -15.4 | -2.5 | -6.3 | -4.4 | -0.4 |
| Liaocheng | -54.2 | -53.4 | -49.8 | -71.6 | -70.3 | -61.6 | -11.1 | -9.4 | -11.0 | -2.3 | -2.8 | -2.3 |
| Linfen | -35.1 | -40.2 | -32.0 | -51.9 | -52.8 | -50.2 | -2.4 | -3.8 | 2.7 | -4.1 | -5.3 | -0.9 |
| Linyi | -38.9 | -36.3 | -26.2 | -82.3 | -55.0 | -57.4 | -10.3 | -9.6 | -9.0 | -2.2 | -2.3 | -1.4 |
| Luohe | -34.8 | -60.3 | -67.7 | -48.1 | -65.5 | -71.2 | -4.9 | -8.7 | -8.0 | 0.5 | -1.7 | -1.2 |
| Luoyang | -37.2 | -34.6 | -34.0 | -33.8 | -34.9 | -32.3 | -3.2 | -2.9 | -1.7 | -0.9 | -0.1 | -0.1 |
| Lvliang | -17.4 | -23.8 | NA | -26.7 | -35.8 | NA | -0.4 | -2.7 | NA | -2.2 | -4.2 | NA |
| Nanyang | -75.2 | -31.5 | -47.2 | -38.6 | -41.6 | -23.7 | 4.8 | -4.5 | -1.1 | 0.5 | -1.2 | -0.4 |
| Pingdingshan | -60.3 | -36.0 | -67.0 | -45.4 | -51.7 | -31.8 | -0.3 | -5.9 | -2.4 | 0.6 | -1.4 | -0.5 |
| Puyang | -75.8 | -48.7 | -43.7 | -63.7 | -62.4 | -54.7 | 0.1 | -6.7 | -5.1 | 1.8 | -1.2 | -0.1 |
| Qingdao | -26.1 | -32.7 | NA | -43.2 | -55.5 | NA | -13.5 | -13.2 | NA | -2.5 | -2.2 | NA |
| Qinhuangdao | -15.0 | -26.4 | NA | -31.3 | -42.8 | NA | -6.3 | -6.3 | NA | -2.1 | -2.1 | NA |
| Rizhao | -37.0 | -30.8 | -18.6 | -51.4 | -51.3 | -42.1 | -9.1 | -9.0 | -6.8 | -3.0 | -2.1 | -0.9 |
| Sanmenxia | -31.4 | -28.9 | -45.1 | -27.6 | -39.5 | -31.5 | -4.7 | -3.5 | -5.1 | -0.9 | -1.6 | 0.0 |
| Shangqiu | -40.2 | -44.8 | -54.9 | -49.8 | -60.7 | -65.2 | -8.7 | -7.0 | -9.7 | -1.3 | -1.7 | -1.3 |
| Shijiazhuang | -43.4 | -50.5 | -77.4 | -44.8 | -77.0 | -72.0 | -9.3 | -13.0 | -21.8 | -1.4 | -3.4 | -5.4 |
| Shuozhou | -24.5 | -25.2 | NA | -59.3 | -50.1 | NA | -4.0 | -4.0 | NA | -1.6 | -4.9 | NA |
| Taian | -32.5 | -37.7 | -7.8 | -45.7 | -59.8 | -43.2 | -6.5 | -7.4 | -2.1 | -2.1 | -2.1 | 0.2 |
| Taiyuan | -32.2 | -32.2 | -32.4 | -54.8 | -56.7 | -50.9 | -4.7 | -6.4 | -3.1 | -3.2 | -5.1 | -7.1 |
| Tangshan | -79.9 | -44.6 | -32.9 | -78.1 | -58.5 | -49.1 | -16.6 | -11.8 | -2.0 | -5.4 | -4.7 | -4.3 |
| Tianjin | -32.8 | -36.5 | NA | -49.6 | -43.5 | NA | -9.8 | -7.2 | NA | -3.1 | -2.6 | NA |
| Weifang | -25.9 | -41.0 | -41.2 | -41.7 | -55.5 | -54.6 | -9.3 | -10.3 | -8.5 | -1.9 | -2.4 | -2.4 |
| Weihai | -20.9 | -21.0 | NA | -35.8 | -40.7 | NA | -7.0 | -5.8 | NA | -2.9 | -0.8 | NA |
| Xinxiang | -100.5 | -54.4 | -64.1 | -36.7 | -59.0 | -70.6 | 2.4 | -5.7 | -8.4 | 1.7 | -0.9 | -1.2 |
| Xinyang | -27.7 | -37.5 | -19.1 | -31.2 | -50.7 | -36.7 | -9.3 | -5.4 | -4.0 | -1.2 | -1.0 | 0.2 |
| Xinzhou | -25.1 | -22.7 | NA | -39.2 | -41.0 | NA | -3.7 | -4.6 | NA | -3.6 | -3.5 | NA |
| Xuchang | -58.7 | -51.1 | -84.4 | -55.9 | -53.9 | -71.5 | -2.2 | -4.3 | -2.6 | -1.0 | -1.5 | -1.6 |
| Yangquan | -31.4 | -35.0 | -47.8 | -45.3 | -44.6 | -71.2 | -9.1 | -8.2 | 0.2 | -4.6 | -3.5 | 11.0 |
| Yantai | -10.4 | -27.8 | NA | -31.2 | -38.9 | NA | -4.6 | -7.7 | NA | -1.4 | -2.1 | NA |
| Yuncheng | -45.9 | -48.3 | -46.5 | -76.2 | -71.9 | -90.7 | -5.8 | -8.5 | -8.1 | -4.0 | -4.3 | -5.2 |
| Zaozhuang | -9.3 | -43.3 | -58.7 | -43.5 | -70.9 | -99.4 | -11.6 | -9.2 | -8.2 | -2.1 | -3.4 | -1.4 |
| Zhangjiakou | -37.5 | NA | NA | -31.8 | NA | NA | -7.6 | NA | NA | -4.3 | NA | NA |
| Zhengzhou | -41.4 | -48.7 | -53.7 | -61.4 | -56.7 | -51.2 | -7.2 | -7.1 | -4.4 | -1.8 | -1.5 | -1.4 |
| Zhoukou | -26.8 | -46.9 | -61.9 | -36.0 | -55.9 | -61.2 | -7.5 | -5.7 | 0.1 | -1.0 | -1.3 | -0.3 |
| Zhumadian | -33.9 | -43.0 | NA | -42.6 | -59.5 | NA | -6.0 | -5.4 | NA | -1.7 | -1.2 | NA |
| Zibo | -60.6 | -41.7 | -50.6 | -103.2 | -52.5 | -50.3 | -17.3 | -10.0 | -7.1 | -10.2 | -4.4 | -2.7 |
| Xingtai | NA | -45.4 | -52.6 | NA | -69.4 | -89.0 | NA | -12.2 | -9.3 | NA | -4.6 | -3.6 |

Table S6. Mean impact of pollution alerts on urban air quality. Values represent the mean (± standard error) differences between observed pollutant concentrations and the model-predicted counterfactual concentrations for the same period assuming no alerts. Negative values therefore signify that air quality improved because of the alerts, whereas positive values indicate a deterioration or no net benefit at the median point.

| **City** | **PM_2.5_ (μg m^-3^)** | | | **PM_10_ (μg m^-3^)** | | | **NO_2_ (μg m^-3^)** | | | **SO_2_ (μg m^-3^)** | | |
| --- | --- | --- | --- | --- | --- | --- | --- | --- | --- | --- | --- | --- |
|  | **Yellow** | **Orange** | **Red** | **Yellow** | **Orange** | **Red** | **Yellow** | **Orange** | **Red** | **Yellow** | **Orange** | **Red** |
| Anyang | -91.5±7.5 | -61.9±2.1 | -59.0±4.7 | -54.6±8.5 | -67.9±2.6 | -58.9±5.5 | -1.9±1.5 | -7.7±0.4 | -5.8±0.9 | 3.9±0.7 | 0.2±0.2 | 1.2±0.5 |
| Baoding | -34.7±4.2 | -47.0±1.4 | -56.1±3.8 | -39.3±5.2 | -69.0±1.7 | -74.4±5.5 | -8.5±1.3 | -14.8±0.4 | -2.0±1.0 | -2.6±0.6 | -4.0±0.2 | -0.0±0.4 |
| Beijing | -28.7±2.7 | -32.5±3.4 | NA | -41.9±4.6 | -39.1±4.6 | NA | -11.4±0.9 | -8.3±0.9 | NA | -3.0±0.2 | -2.1±0.2 | NA |
| Binzhou | -32.2±3.9 | -34.7±2.2 | -43.5±4.9 | -45.3±4.0 | -51.4±2.5 | -39.4±1.2 | -8.3±1.2 | -10.8±0.6 | -9.1±2.2 | -5.6±0.4 | -4.8±0.3 | -0.7±1.5 |
| Cangzhou | -45.5±4.3 | -50.2±1.6 | -42.5±4.0 | -56.7±4.6 | -60.4±1.8 | -61.0±4.8 | -12.9±0.9 | -11.6±0.4 | -13.9±1.7 | -3.1±0.5 | -3.9±0.2 | -4.7±0.5 |
| Changzhi | -29.4±2.8 | -27.8±1.0 | -55.3±9.1 | -45.5±2.7 | -46.0±1.4 | -68.7±11.3 | -3.8±0.7 | -3.9±0.4 | -5.1±3.2 | -2.4±0.3 | -1.9±0.2 | -0.7±0.7 |
| Chengde | -22.1±5.2 | -18.4±5.1 | NA | -15.4±13.4 | -32.6±7.2 | NA | -4.0±1.1 | -8.4±0.8 | NA | -2.1±0.4 | -3.5±0.1 | NA |
| Datong | -27.7±3.8 | -33.4±2.1 | -30.6±3.2 | -48.2±4.9 | -54.5±2.8 | -57.6±2.9 | -6.9±1.0 | -10.7±1.0 | -2.8±2.5 | -2.9±0.8 | -6.8±0.8 | -9.4±0.9 |
| Dezhou | -66.5±8.3 | -41.3±2.2 | NA | -98.2±9.8 | -67.4±2.6 | NA | -10.4±1.6 | -11.0±0.5 | NA | -2.2±0.8 | -3.8±0.3 | NA |
| Dongying | -52.0±4.6 | -35.5±2.8 | -19.8±8.1 | -72.3±2.8 | -55.7±2.9 | -52.9±5.9 | -12.5±1.6 | -13.6±0.7 | -12.1±1.3 | -2.2±0.5 | -3.1±0.3 | -1.9±0.9 |
| Handan | -43.3±3.8 | -45.3±1.6 | -37.8±4.1 | -49.6±4.3 | -68.8±2.3 | -71.4±6.4 | -8.2±1.2 | -12.6±0.4 | -9.1±1.1 | -0.8±0.9 | -5.8±0.2 | -5.7±0.5 |
| Hebi | -80.2±8.7 | -55.4±2.2 | -58.2±5.2 | -57.0±9.5 | -57.7±2.6 | -62.2±5.8 | -5.9±1.4 | -7.6±0.5 | -5.4±1.2 | 1.1±0.8 | -0.9±0.2 | 0.2±0.4 |
| Hengshui | -60.2±5.7 | -56.4±1.9 | -41.5±5.7 | -47.2±9.3 | -71.0±2.3 | -74.6±6.4 | -8.9±2.1 | -13.3±0.5 | -2.6±1.7 | -2.8±0.9 | -3.5±0.2 | -2.4±0.9 |
| Heze | -61.0±12.3 | -50.1±2.3 | -44.3±5.3 | -79.5±9.8 | -55.1±2.3 | -59.7±5.4 | -5.8±2.7 | -7.1±0.5 | -6.6±1.2 | 0.3±0.8 | -2.4±0.2 | -2.5±0.5 |
| Jiaozuo | -48.5±5.7 | -46.1±2.0 | -40.6±4.5 | -30.1±10.2 | -52.6±2.4 | -52.9±5.3 | -1.8±1.8 | -5.6±0.5 | -5.0±1.0 | -0.1±0.4 | -1.0±0.2 | -0.5±0.4 |
| Jinan | -122.5±15.4 | -46.5±2.0 | -35.6±7.7 | -134.4±20.9 | -57.3±2.6 | -42.3±8.7 | -10.8±2.8 | -9.8±0.5 | -9.5±1.0 | -2.9±0.9 | -3.3±0.3 | -0.4±0.7 |
| Jincheng | -29.9±1.9 | -25.0±1.1 | NA | -38.8±3.0 | -41.5±1.6 | NA | -1.4±0.7 | -2.8±0.3 | NA | -1.4±0.5 | -1.6±0.2 | NA |
| Jining | -52.9±4.5 | -47.6±1.8 | -59.2±3.4 | -52.6±4.0 | -54.9±1.9 | -57.5±5.2 | -8.6±1.1 | -8.8±0.5 | -9.0±1.0 | -2.1±0.3 | -3.7±0.2 | -3.4±0.6 |
| Jinzhong | -28.8±1.7 | -30.3±1.5 | -16.0±6.0 | -42.0±2.5 | -46.4±2.1 | -37.7±8.2 | -2.5±0.6 | -2.8±0.5 | 4.8±1.6 | -2.0±0.5 | -3.5±0.3 | -5.4±1.3 |
| Kaifeng | -54.6±6.5 | -49.0±2.1 | -53.6±4.7 | -73.0±7.9 | -62.2±2.6 | -74.9±6.2 | -9.9±0.8 | -7.4±0.5 | -7.0±1.1 | -0.9±0.6 | -0.5±0.2 | 0.0±0.4 |
| Langfang | -61.0±5.1 | -59.3±1.9 | -46.1±6.3 | -83.0±5.2 | -75.0±2.2 | -72.3±8.8 | -20.5±1.5 | -15.1±0.5 | 0.5±1.6 | -6.5±0.7 | -4.6±0.2 | -0.1±0.6 |
| Liaocheng | -59.7±6.9 | -54.2±2.3 | -50.2±5.2 | -68.7±7.1 | -66.0±2.6 | -70.5±5.7 | -11.4±1.6 | -8.9±0.6 | -11.0±0.9 | -1.5±0.7 | -2.6±0.2 | -2.3±0.5 |
| Linfen | -36.0±2.0 | -40.7±1.1 | -36.2±5.3 | -53.4±3.3 | -56.0±1.6 | -51.3±7.7 | -2.6±0.6 | -3.6±0.3 | 2.7±1.0 | -4.6±0.4 | -5.4±0.2 | -1.2±1.0 |
| Linyi | -39.6±6.1 | -38.8±2.4 | -36.3±8.0 | -79.5±6.0 | -54.9±2.8 | -54.8±8.0 | -8.6±1.9 | -9.7±0.5 | -8.0±1.1 | -2.2±0.3 | -2.3±0.2 | -1.2±0.6 |
| Luohe | -40.6±7.7 | -63.4±2.9 | -69.2±8.6 | -47.3±5.8 | -63.3±2.5 | -63.2±8.5 | -4.8±1.5 | -7.9±0.5 | -8.2±1.3 | -0.6±0.7 | -1.7±0.2 | -1.3±0.4 |
| Luoyang | -37.6±2.7 | -34.5±1.4 | -33.1±3.3 | -36.8±2.9 | -33.3±1.8 | -29.1±3.7 | -3.1±0.6 | -3.1±0.4 | -0.9±0.7 | -0.8±0.2 | 0.2±0.1 | 0.1±0.2 |
| Lvliang | -17.1±1.9 | -23.0±0.9 | NA | -28.2±3.1 | -34.1±1.3 | NA | -0.5±0.7 | -2.5±0.3 | NA | -1.4±0.8 | -4.4±0.2 | NA |
| Nanyang | -71.0±5.3 | -34.3±1.9 | -44.7±9.6 | -36.0±9.2 | -41.4±1.9 | -25.3±8.8 | 4.4±1.5 | -3.9±0.4 | -0.3±1.2 | 0.2±0.3 | -1.1±0.1 | -0.4±0.3 |
| Pingdingshan | -60.3±7.1 | -40.7±2.4 | -55.7±11.5 | -43.2±6.7 | -48.4±2.3 | -18.9±12.7 | 1.0±1.6 | -5.5±0.5 | -1.9±1.5 | 0.4±0.4 | -1.3±0.1 | 0.1±0.5 |
| Puyang | -86.0±10.1 | -49.4±2.4 | -50.9±3.2 | -67.6±13.8 | -60.5±3.0 | -53.2±3.2 | -1.3±1.9 | -5.4±0.6 | -4.2±0.9 | 0.9±0.6 | -0.6±0.2 | -0.1±0.3 |
| Qingdao | -29.0±3.7 | -32.1±5.7 | NA | -47.7±4.2 | -51.2±7.0 | NA | -13.0±1.1 | -12.7±1.2 | NA | -2.5±0.4 | -1.8±0.5 | NA |
| Qinhuangdao | -16.5±5.4 | -27.4±1.3 | NA | -25.3±7.0 | -41.3±1.6 | NA | -5.7±1.7 | -6.7±0.4 | NA | -2.1±0.4 | -1.9±0.2 | NA |
| Rizhao | -37.1±3.2 | -34.3±3.0 | -23.9±7.2 | -56.5±3.9 | -52.0±3.5 | -42.3±6.1 | -9.3±0.8 | -9.1±0.8 | -6.6±1.3 | -2.9±0.3 | -2.0±0.4 | -0.0±0.5 |
| Sanmenxia | -29.1±2.3 | -28.5±1.3 | -44.0±4.2 | -30.4±2.5 | -35.5±1.6 | -34.7±4.6 | -5.7±0.9 | -3.2±0.3 | -4.0±1.2 | -0.4±0.4 | -1.6±0.1 | 0.4±0.6 |
| Shangqiu | -44.7±5.6 | -45.9±2.3 | -40.0±10.8 | -46.3±5.9 | -58.9±2.5 | -65.2±7.8 | -7.1±1.1 | -6.8±0.5 | -6.3±3.2 | -1.3±0.3 | -2.0±0.2 | -2.0±0.7 |
| Shijiazhuang | -39.3±6.0 | -55.1±1.7 | -77.1±7.7 | -43.5±8.5 | -78.0±2.0 | -81.0±6.2 | -9.3±1.1 | -13.1±0.4 | -19.1±1.5 | -1.3±0.5 | -3.6±0.2 | -5.4±0.4 |
| Shuozhou | -24.5±2.4 | -27.3±1.2 | NA | -61.8±6.2 | -49.5±1.8 | NA | -4.1±0.8 | -4.1±0.4 | NA | -2.3±0.7 | -4.8±0.3 | NA |
| Taian | -32.5±3.8 | -37.3±2.6 | -7.5±4.3 | -50.0±4.6 | -57.7±3.0 | -43.0±2.8 | -6.9±0.7 | -7.8±0.5 | -2.9±1.1 | -1.8±0.4 | -2.1±0.2 | 0.7±0.6 |
| Taiyuan | -33.2±1.4 | -32.0±1.4 | -27.5±4.2 | -59.8±3.0 | -57.2±1.9 | -46.5±6.2 | -5.3±0.6 | -6.2±0.5 | -2.7±1.3 | -3.4±0.4 | -5.8±0.3 | -6.7±0.7 |
| Tangshan | -58.3±15.8 | -48.5±1.3 | -34.3±2.2 | -62.4±19.3 | -60.3±1.6 | -48.6±5.5 | -16.7±2.1 | -12.0±0.4 | -1.5±1.3 | -7.5±1.4 | -4.6±0.2 | -4.1±0.7 |
| Tianjin | -32.9±2.9 | -35.6±3.7 | NA | -42.0±3.9 | -42.5±4.7 | NA | -10.4±0.9 | -6.5±1.3 | NA | -3.0±0.2 | -2.6±0.3 | NA |
| Weifang | -30.7±6.7 | -40.7±2.9 | -42.7±7.8 | -40.6±6.9 | -53.3±3.2 | -49.2±5.9 | -8.5±0.9 | -11.2±0.7 | -9.0±1.0 | -2.1±0.4 | -2.4±0.3 | -1.2±0.9 |
| Weihai | -23.1±2.8 | -21.5±5.8 | NA | -37.6±4.7 | -46.3±8.5 | NA | -7.9±0.9 | -2.7±1.5 | NA | -2.4±0.4 | -0.7±0.4 | NA |
| Xinxiang | -103.1±10.0 | -55.6±2.4 | -66.7±3.8 | -24.7±12.1 | -59.0±2.5 | -61.5±4.3 | 5.8±2.4 | -5.8±0.5 | -7.5±0.7 | 1.4±0.5 | -0.5±0.2 | -0.7±0.3 |
| Xinyang | -26.5±4.4 | -38.7±2.1 | -27.6±10.0 | -29.7±4.4 | -50.4±2.6 | -46.3±10.4 | -9.3±1.1 | -5.3±0.5 | -3.3±2.4 | -1.1±0.4 | -0.8±0.1 | 0.2±0.5 |
| Xinzhou | -24.8±1.2 | -22.4±0.8 | NA | -36.9±2.6 | -42.1±1.2 | NA | -3.6±0.5 | -4.3±0.3 | NA | -3.6±0.2 | -3.6±0.2 | NA |
| Xuchang | -66.1±7.0 | -55.7±2.9 | -79.2±6.5 | -50.7±6.8 | -54.9±2.6 | -64.9±6.0 | -1.0±1.4 | -4.8±0.6 | -3.7±1.4 | -0.6±0.4 | -1.5±0.2 | -1.4±0.4 |
| Yangquan | -35.5±3.6 | -38.0±1.3 | -52.8±11.6 | -47.5±4.2 | -46.0±1.6 | -72.7±21.4 | -9.7±1.1 | -8.5±0.4 | -5.8±3.4 | -4.6±0.8 | -3.1±0.3 | 9.1±1.9 |
| Yantai | -9.7±4.0 | -25.4±2.3 | NA | -25.0±5.5 | -36.3±3.1 | NA | -4.3±1.3 | -7.7±0.6 | NA | -1.2±0.6 | -2.1±0.2 | NA |
| Yuncheng | -49.7±2.6 | -48.6±1.8 | -49.9±6.8 | -79.9±3.6 | -76.2±2.3 | -87.7±7.6 | -5.7±0.6 | -8.2±0.4 | -6.6±1.4 | -4.2±0.3 | -4.3±0.2 | -5.0±0.7 |
| Zaozhuang | -9.4±9.1 | -43.0±2.4 | -51.6±5.9 | -40.4±8.6 | -68.4±2.8 | -88.0±8.4 | -11.0±1.6 | -9.5±0.6 | -8.0±1.8 | -2.8±0.6 | -3.5±0.2 | -1.8±1.0 |
| Zhangjiakou | -40.7±9.5 | NA | NA | -33.7±17.2 | NA | NA | -7.5±0.6 | NA | NA | -3.9±0.4 | NA | NA |
| Zhengzhou | -43.1±2.9 | -51.1±2.3 | -54.9±6.4 | -59.5±4.5 | -57.1±2.5 | -55.3±6.6 | -7.0±0.6 | -6.8±0.5 | -4.6±0.9 | -1.9±0.2 | -1.4±0.2 | -1.0±0.3 |
| Zhoukou | -32.8±7.2 | -47.8±3.3 | -65.0±8.1 | -37.2±6.4 | -51.7±2.6 | -60.2±6.7 | -5.6±1.0 | -5.6±0.5 | -1.3±1.6 | -0.8±0.3 | -1.4±0.2 | -0.1±0.3 |
| Zhumadian | -28.3±7.5 | -44.1±2.1 | NA | -47.5±7.3 | -57.3±2.2 | NA | -4.5±1.2 | -5.1±0.4 | NA | -1.1±0.4 | -0.9±0.1 | NA |
| Zibo | -61.8±4.9 | -39.2±1.8 | -46.0±5.6 | -102.7±5.4 | -49.4±2.2 | -53.4±5.2 | -16.4±1.7 | -9.3±0.4 | -8.4±1.2 | -11.1±1.5 | -4.8±0.2 | -3.0±0.8 |
| Xingtai | NA | -48.8±1.7 | -53.3±3.0 | NA | -68.9±2.4 | -93.2±6.0 | NA | -12.2±0.4 | -9.5±1.2 | NA | -4.8±0.2 | -3.9±0.5 |

Table S7. Gradient Boosting Model (GBM) performance by city for predicting air pollutant concentrations (model trained using data from air quality monitoring stations). GBM models were trained on daily meteorological factors, trend, year, month, and day of week (dow) from 2018 to 2020 for each pollutant, excluding alert (all levels) and transition dates. Model performance was evaluated using the revised index of agreement (IOA), correlation coefficient (*r*), and root mean square error (RMSE). An IOA above 0.5 signifies good model performance, and the average *r* indicates strong predictive accuracy across models.

| **City** | **PM_2.5_** | | | **PM_10_** | | | **NO_2_** | | | **SO_2_** | | |
| --- | --- | --- | --- | --- | --- | --- | --- | --- | --- | --- | --- | --- |
|  | ***r*** | **RMSE** | **IOA** | ***r*** | **RMSE** | **IOA** | ***r*** | **RMSE** | **IOA** | ***r*** | **RMSE** | **IOA** |
| Anyang | 0.73 | 29.06 | 0.67 | 0.56 | 66.95 | 0.64 | 0.83 | 9.28 | 0.74 | 0.85 | 6.31 | 0.74 |
| Baoding | 0.81 | 29.32 | 0.70 | 0.76 | 40.17 | 0.67 | 0.87 | 10.37 | 0.76 | 0.83 | 8.61 | 0.77 |
| Beijing | 0.70 | 23.91 | 0.65 | 0.48 | 56.53 | 0.64 | 0.90 | 8.09 | 0.79 | 0.75 | 2.44 | 0.71 |
| Binzhou | 0.71 | 22.02 | 0.65 | 0.66 | 43.83 | 0.67 | 0.85 | 9.34 | 0.75 | 0.81 | 7.18 | 0.72 |
| Cangzhou | 0.76 | 20.49 | 0.68 | 0.65 | 42.65 | 0.67 | 0.83 | 8.83 | 0.74 | 0.88 | 6.79 | 0.81 |
| Changzhi | 0.71 | 17.43 | 0.65 | 0.67 | 34.51 | 0.66 | 0.84 | 6.99 | 0.74 | 0.93 | 8.37 | 0.81 |
| Chengde | 0.63 | 14.48 | 0.64 | 0.64 | 34.04 | 0.66 | 0.86 | 6.53 | 0.75 | 0.83 | 4.02 | 0.75 |
| Datong | 0.57 | 19.74 | 0.64 | 0.54 | 53.60 | 0.62 | 0.82 | 6.83 | 0.73 | 0.84 | 10.29 | 0.74 |
| Dezhou | 0.75 | 20.95 | 0.67 | 0.69 | 46.89 | 0.67 | 0.88 | 7.34 | 0.79 | 0.85 | 4.85 | 0.74 |
| Dongying | 0.72 | 24.01 | 0.66 | 0.70 | 40.51 | 0.69 | 0.86 | 9.38 | 0.77 | 0.89 | 6.87 | 0.79 |
| Handan | 0.81 | 27.22 | 0.70 | 0.69 | 57.60 | 0.68 | 0.87 | 9.19 | 0.77 | 0.83 | 10.08 | 0.74 |
| Hebi | 0.74 | 23.14 | 0.66 | 0.68 | 49.31 | 0.65 | 0.83 | 9.18 | 0.73 | 0.86 | 5.39 | 0.76 |
| Hengshui | 0.62 | 25.65 | 0.62 | 0.66 | 45.86 | 0.67 | 0.86 | 7.86 | 0.75 | 0.81 | 4.46 | 0.68 |
| Heze | 0.74 | 23.02 | 0.67 | 0.59 | 58.42 | 0.64 | 0.86 | 7.19 | 0.76 | 0.85 | 4.48 | 0.74 |
| Jiaozuo | 0.74 | 22.97 | 0.68 | 0.69 | 44.78 | 0.67 | 0.86 | 7.60 | 0.75 | 0.87 | 4.55 | 0.76 |
| Jinan | 0.74 | 21.65 | 0.64 | 0.72 | 37.77 | 0.66 | 0.83 | 9.24 | 0.73 | 0.90 | 4.11 | 0.78 |
| Jincheng | 0.66 | 20.62 | 0.64 | 0.53 | 55.39 | 0.64 | 0.81 | 9.01 | 0.72 | 0.87 | 10.05 | 0.80 |
| Jining | 0.73 | 21.14 | 0.66 | 0.55 | 52.19 | 0.66 | 0.87 | 7.60 | 0.76 | 0.87 | 4.44 | 0.77 |
| Jinzhong | 0.70 | 20.09 | 0.65 | 0.58 | 70.32 | 0.65 | 0.83 | 8.35 | 0.74 | 0.94 | 14.51 | 0.83 |
| Kaifeng | 0.77 | 27.19 | 0.70 | 0.64 | 45.58 | 0.65 | 0.84 | 8.56 | 0.74 | 0.85 | 4.39 | 0.76 |
| Langfang | 0.73 | 20.54 | 0.68 | 0.54 | 53.51 | 0.66 | 0.88 | 8.53 | 0.76 | 0.83 | 4.69 | 0.71 |
| Liaocheng | 0.67 | 24.44 | 0.66 | 0.58 | 53.10 | 0.65 | 0.83 | 8.56 | 0.73 | 0.74 | 5.37 | 0.67 |
| Linfen | 0.79 | 24.48 | 0.69 | 0.60 | 68.42 | 0.68 | 0.80 | 8.41 | 0.70 | 0.95 | 19.04 | 0.83 |
| Linyi | 0.79 | 25.31 | 0.72 | 0.73 | 43.78 | 0.69 | 0.89 | 7.89 | 0.78 | 0.83 | 6.61 | 0.74 |
| Luohe | 0.79 | 28.99 | 0.71 | 0.73 | 48.58 | 0.69 | 0.88 | 7.25 | 0.77 | 0.74 | 3.43 | 0.70 |
| Luoyang | 0.76 | 22.78 | 0.71 | 0.62 | 52.60 | 0.63 | 0.83 | 8.64 | 0.73 | 0.88 | 4.24 | 0.79 |
| Lvliang | 0.78 | 16.87 | 0.72 | 0.63 | 45.18 | 0.62 | 0.73 | 7.58 | 0.66 | 0.93 | 13.40 | 0.86 |
| Nanyang | 0.74 | 24.42 | 0.70 | 0.69 | 42.73 | 0.68 | 0.87 | 7.22 | 0.77 | 0.86 | 2.54 | 0.76 |
| Pingdingshan | 0.80 | 23.69 | 0.71 | 0.57 | 57.18 | 0.64 | 0.82 | 8.52 | 0.72 | 0.87 | 5.02 | 0.77 |
| Puyang | 0.73 | 25.47 | 0.68 | 0.62 | 61.92 | 0.66 | 0.85 | 7.84 | 0.75 | 0.85 | 3.99 | 0.74 |
| Qingdao | 0.76 | 19.19 | 0.68 | 0.70 | 32.59 | 0.66 | 0.86 | 8.93 | 0.76 | 0.87 | 2.93 | 0.78 |
| Qinhuangdao | 0.72 | 17.01 | 0.66 | 0.71 | 31.70 | 0.67 | 0.85 | 8.98 | 0.74 | 0.83 | 6.26 | 0.75 |
| Rizhao | 0.75 | 21.34 | 0.68 | 0.66 | 44.81 | 0.68 | 0.81 | 9.94 | 0.73 | 0.83 | 3.31 | 0.73 |
| Sanmenxia | 0.65 | 26.68 | 0.67 | 0.51 | 60.30 | 0.63 | 0.83 | 8.06 | 0.73 | 0.90 | 3.74 | 0.79 |
| Shangqiu | 0.79 | 23.17 | 0.72 | 0.76 | 35.77 | 0.69 | 0.82 | 8.24 | 0.73 | 0.80 | 2.81 | 0.70 |
| Shijiazhuang | 0.74 | 29.64 | 0.66 | 0.77 | 42.08 | 0.67 | 0.84 | 10.56 | 0.73 | 0.94 | 6.15 | 0.82 |
| Shuozhou | 0.75 | 19.07 | 0.69 | 0.40 | 105.96 | 0.64 | 0.77 | 8.39 | 0.68 | 0.89 | 12.16 | 0.79 |
| Taian | 0.75 | 22.10 | 0.68 | 0.70 | 42.55 | 0.65 | 0.86 | 7.61 | 0.76 | 0.84 | 5.02 | 0.73 |
| Taiyuan | 0.71 | 23.69 | 0.67 | 0.70 | 46.25 | 0.67 | 0.82 | 9.59 | 0.73 | 0.89 | 13.46 | 0.78 |
| Tangshan | 0.72 | 22.30 | 0.67 | 0.69 | 47.45 | 0.69 | 0.85 | 9.81 | 0.74 | 0.85 | 10.11 | 0.78 |
| Tianjin | 0.69 | 24.69 | 0.65 | 0.62 | 41.16 | 0.66 | 0.88 | 8.85 | 0.78 | 0.82 | 3.69 | 0.72 |
| Weifang | 0.71 | 25.74 | 0.66 | 0.74 | 38.90 | 0.68 | 0.86 | 8.14 | 0.76 | 0.87 | 5.03 | 0.77 |
| Weihai | 0.65 | 14.32 | 0.63 | 0.60 | 27.32 | 0.64 | 0.84 | 5.05 | 0.73 | 0.80 | 2.46 | 0.74 |
| Xingtai | 0.74 | 33.92 | 0.70 | 0.67 | 58.28 | 0.68 | 0.86 | 9.97 | 0.74 | 0.90 | 8.35 | 0.80 |
| Xinxiang | 0.71 | 23.17 | 0.67 | 0.67 | 43.03 | 0.67 | 0.84 | 9.20 | 0.74 | 0.88 | 5.10 | 0.78 |
| Xinyang | 0.73 | 25.44 | 0.70 | 0.72 | 35.15 | 0.68 | 0.88 | 5.86 | 0.79 | 0.84 | 2.08 | 0.75 |
| Xinzhou | 0.75 | 22.26 | 0.68 | 0.67 | 40.27 | 0.64 | 0.83 | 8.78 | 0.74 | 0.90 | 11.30 | 0.79 |
| Xuchang | 0.77 | 25.17 | 0.69 | 0.72 | 41.55 | 0.69 | 0.84 | 8.57 | 0.74 | 0.85 | 4.65 | 0.76 |
| Yangquan | 0.64 | 19.72 | 0.64 | 0.68 | 41.38 | 0.67 | 0.82 | 8.41 | 0.71 | 0.86 | 12.08 | 0.76 |
| Yantai | 0.73 | 15.92 | 0.68 | 0.64 | 34.42 | 0.65 | 0.89 | 6.37 | 0.78 | 0.86 | 3.22 | 0.75 |
| Yuncheng | 0.79 | 22.38 | 0.70 | 0.63 | 62.16 | 0.69 | 0.87 | 8.08 | 0.76 | 0.92 | 9.97 | 0.83 |
| Zaozhuang | 0.78 | 22.97 | 0.70 | 0.77 | 36.45 | 0.69 | 0.88 | 6.54 | 0.77 | 0.83 | 5.23 | 0.73 |
| Zhangjiakou | 0.53 | 19.15 | 0.64 | 0.37 | 97.44 | 0.64 | 0.85 | 5.42 | 0.75 | 0.89 | 3.15 | 0.80 |
| Zhengzhou | 0.72 | 23.66 | 0.67 | 0.69 | 42.23 | 0.66 | 0.86 | 10.00 | 0.75 | 0.91 | 3.30 | 0.80 |
| Zhoukou | 0.76 | 26.76 | 0.71 | 0.70 | 47.01 | 0.69 | 0.87 | 6.35 | 0.76 | 0.85 | 3.02 | 0.76 |
| Zhumadian | 0.80 | 20.52 | 0.72 | 0.70 | 35.46 | 0.66 | 0.84 | 7.39 | 0.74 | 0.87 | 2.90 | 0.76 |
| Zibo | 0.64 | 22.27 | 0.62 | 0.64 | 36.67 | 0.65 | 0.82 | 9.07 | 0.72 | 0.89 | 6.71 | 0.80 |
| **Average** | **0.73** | **22.87** | **0.68** | **0.65** | **48.28** | **0.66** | **0.85** | **8.23** | **0.75** | **0.86** | **6.29** | **0.76** |

Table S8. Median impact of pollution alerts on urban air quality (model trained using data from air quality monitoring stations). The values are the median differences between the pollutant concentrations observed and the counterfactual concentrations estimated for the same period had no alerts been issued. Negative values therefore signify that air quality improved because of the alerts, whereas positive values indicate a deterioration or no net benefit at the median point.

| **City** | **PM_2.5_ (μg m^-3^)** | | | **PM_10_ (μg m^-3^)** | | | **NO_2_ (μg m^-3^)** | | | **SO_2_ (μg m^-3^)** | | |
| --- | --- | --- | --- | --- | --- | --- | --- | --- | --- | --- | --- | --- |
|  | **Yellow** | **Orange** | **Red** | **Yellow** | **Orange** | **Red** | **Yellow** | **Orange** | **Red** | **Yellow** | **Orange** | **Red** |
| Anyang | -74.3 | -34.9 | 2.3 | 0.1 | -53.1 | -61.3 | -1.0 | -9.4 | -56.2 | -44.3 | 0.9 | -5.4 |
| Baoding | -35.3 | -59.8 | -4.0 | -14.3 | -46.9 | -93.3 | -4.2 | -19.4 | -50.1 | -95.8 | -1.7 | -10.1 |
| Beijing | -22.7 | -49.5 | -1.2 | -10.7 | -38.0 | -39.3 | -0.6 | -8.9 | NA | NA | NA | NA |
| Binzhou | -37.0 | -47.2 | -8.2 | -9.0 | -35.6 | -50.7 | -6.1 | -11.7 | -11.9 | -44.6 | 1.1 | -8.3 |
| Cangzhou | -41.3 | -57.7 | -5.3 | -16.3 | -43.1 | -58.7 | -5.3 | -13.4 | -37.2 | -59.9 | -5.6 | -17.4 |
| Changzhi | -33.8 | -60.4 | -1.8 | -2.8 | -30.0 | -47.4 | -1.1 | -4.2 | -34.6 | -68.3 | 1.6 | -6.6 |
| Chengde | -43.8 | -18.0 | -3.8 | -6.3 | -42.3 | -51.3 | -1.9 | -15.5 | NA | NA | NA | NA |
| Datong | -28.3 | -39.0 | -8.1 | -3.9 | -29.8 | -56.4 | -7.7 | -6.2 | -20.4 | -17.1 | -16.5 | 8.3 |
| Dezhou | -61.8 | -79.9 | -2.3 | -1.3 | -42.7 | -60.9 | -4.4 | -9.7 | NA | NA | NA | NA |
| Dongying | -48.4 | -56.3 | -2.2 | -9.5 | -34.9 | -56.9 | -4.1 | -12.9 | -26.9 | -37.2 | -2.2 | -15.5 |
| Handan | -57.1 | -73.2 | -5.4 | -11.9 | -43.1 | -64.1 | -7.0 | -12.5 | -37.7 | -78.3 | -8.2 | -11.9 |
| Hebi | -62.7 | -61.3 | 0.5 | -4.5 | -55.3 | -63.8 | -2.0 | -7.5 | -62.1 | -72.4 | 0.2 | -12.3 |
| Hengshui | -79.0 | -56.8 | -3.7 | -11.8 | -54.4 | -68.2 | -3.5 | -13.9 | -42.3 | -57.2 | -0.3 | -6.1 |
| Heze | -75.7 | -91.2 | -2.1 | 1.0 | -47.5 | -64.4 | -4.1 | -8.3 | -50.9 | -63.3 | -4.3 | -7.3 |
| Jiaozuo | -54.1 | -35.6 | -0.6 | 0.8 | -48.3 | -52.8 | -1.9 | -3.4 | -43.4 | -51.5 | -2.2 | -2.4 |
| Jinan | -102.4 | -124.7 | -2.6 | -16.2 | -41.7 | -61.4 | -4.2 | -10.8 | -25.3 | -37.5 | -0.4 | -12.3 |
| Jincheng | -29.6 | -60.2 | -0.1 | -4.3 | -21.9 | -50.3 | 2.0 | -3.4 | NA | NA | NA | NA |
| Jining | -41.2 | -48.1 | -1.0 | -8.1 | -47.2 | -57.2 | -4.9 | -8.3 | -50.4 | -61.9 | -5.2 | -10.3 |
| Jinzhong | -33.7 | -46.0 | -1.4 | -5.9 | -28.1 | -47.9 | -2.5 | -4.7 | -28.6 | -43.9 | 3.0 | 0.0 |
| Kaifeng | -49.3 | -68.5 | -2.1 | -3.3 | -47.5 | -68.9 | -1.2 | -7.2 | -55.7 | -71.8 | -1.3 | -4.3 |
| Langfang | -47.7 | -84.5 | -3.2 | -16.3 | -45.5 | -68.4 | -3.6 | -14.9 | -53.4 | -52.4 | -2.0 | -1.7 |
| Liaocheng | -58.7 | -82.6 | -2.5 | -9.2 | -50.9 | -71.7 | -4.6 | -11.1 | -46.2 | -77.4 | -4.5 | -8.8 |
| Linfen | -37.4 | -46.2 | -5.6 | -5.6 | -49.5 | -60.2 | -3.6 | -8.9 | -31.6 | -19.7 | 14.1 | -3.6 |
| Linyi | -51.7 | -90.1 | -2.4 | -5.1 | -45.7 | -76.5 | -2.5 | -11.6 | -36.6 | -80.1 | 3.3 | -10.2 |
| Luohe | -36.1 | -43.1 | 1.3 | -5.6 | -59.1 | -69.3 | -1.9 | -7.7 | -75.5 | -76.6 | -1.2 | -4.2 |
| Luoyang | -44.1 | -36.3 | -0.1 | 1.8 | -34.4 | -34.6 | -0.8 | -2.6 | -41.9 | -38.2 | 0.5 | 0.3 |
| Lvliang | -17.7 | -43.1 | -6.4 | -2.4 | -22.3 | -54.5 | -3.8 | -2.6 | NA | NA | NA | NA |
| Nanyang | -83.5 | -56.9 | -1.5 | 4.4 | -36.0 | -50.1 | -1.3 | -6.6 | -55.8 | -40.6 | -0.8 | -0.6 |
| Pingdingshan | -45.1 | -63.4 | -0.3 | -0.1 | -45.0 | -65.3 | -2.7 | -5.0 | -61.7 | -48.2 | -1.9 | -3.4 |
| Puyang | -50.1 | -47.2 | -0.8 | 2.8 | -50.7 | -60.0 | -2.1 | -7.8 | -68.7 | -60.6 | -1.8 | -5.9 |
| Qingdao | -29.4 | -53.1 | -2.1 | -14.8 | -32.0 | -36.4 | -1.4 | -17.4 | NA | NA | NA | NA |
| Qinhuangdao | -25.0 | -46.5 | -1.8 | -5.2 | -26.6 | -42.0 | -2.7 | -7.5 | NA | NA | NA | NA |
| Rizhao | -31.5 | -53.1 | -2.3 | -7.0 | -20.3 | -47.6 | -2.3 | -6.3 | -7.5 | -23.2 | -2.2 | -2.8 |
| Sanmenxia | -32.4 | -38.6 | 0.2 | -5.1 | -35.3 | -49.4 | -1.4 | -3.6 | -58.8 | -32.7 | 0.0 | -2.3 |
| Shangqiu | -42.1 | -56.8 | -0.2 | -11.3 | -47.5 | -59.4 | -2.4 | -9.3 | -58.0 | -75.4 | -3.5 | -13.3 |
| Shijiazhuang | -39.9 | -64.0 | -1.8 | -13.1 | -59.6 | -84.6 | -3.5 | -15.6 | -75.9 | -120.7 | -8.9 | -25.5 |
| Shuozhou | -28.5 | -92.0 | -3.4 | -3.4 | -37.6 | -89.8 | -9.4 | -6.1 | NA | NA | NA | NA |
| Taian | -29.3 | -61.0 | -2.0 | -6.7 | -36.7 | -64.9 | -2.4 | -7.6 | -24.9 | -80.1 | 2.7 | -5.7 |
| Taiyuan | -54.8 | -76.7 | -4.9 | -8.8 | -54.4 | -78.5 | -7.7 | -9.8 | -43.4 | -51.0 | -8.9 | 0.2 |
| Tangshan | -79.2 | -75.7 | -15.8 | -18.9 | -50.5 | -78.8 | -6.1 | -16.0 | -35.5 | -73.3 | -4.8 | 0.5 |
| Tianjin | -25.1 | -42.6 | -2.7 | -10.4 | -32.2 | -44.2 | -1.8 | -8.1 | NA | NA | NA | NA |
| Weifang | -23.9 | -41.0 | -3.6 | -11.8 | -37.0 | -64.7 | -2.8 | -10.8 | -47.6 | -64.4 | 0.3 | -10.3 |
| Weihai | -17.3 | -26.8 | -1.3 | -3.7 | -21.5 | -41.7 | -1.3 | -3.7 | NA | NA | NA | NA |
| Xinxiang | -83.2 | -23.7 | 6.9 | 10.2 | -58.8 | -68.8 | -0.3 | -4.9 | -70.7 | -74.5 | -0.3 | -9.3 |
| Xinyang | -46.3 | -33.0 | -1.6 | -5.6 | -43.6 | -53.5 | -1.2 | -6.8 | -51.7 | -53.7 | 0.1 | -9.7 |
| Xinzhou | -26.0 | -42.7 | -3.3 | -8.2 | -26.9 | -42.2 | -2.2 | -6.9 | NA | NA | NA | NA |
| Xuchang | -75.0 | -51.0 | -0.2 | -5.0 | -49.7 | -58.9 | -2.4 | -8.0 | -71.5 | -58.5 | -1.9 | -6.5 |
| Yangquan | -46.5 | -48.7 | -5.4 | -15.0 | -41.7 | -54.6 | -4.3 | -11.0 | -45.8 | -73.3 | 21.9 | 3.5 |
| Yantai | -5.9 | -22.3 | -1.3 | -1.2 | -20.7 | -43.6 | -1.8 | -5.2 | NA | NA | NA | NA |
| Yuncheng | -52.8 | -70.5 | -3.0 | -6.8 | -56.6 | -78.5 | -4.1 | -8.7 | -70.8 | -96.8 | -7.8 | -14.5 |
| Zaozhuang | -9.0 | -42.8 | -5.3 | -5.3 | -41.3 | -74.4 | -4.7 | -11.4 | -42.9 | -91.1 | -3.9 | -16.4 |
| Zhangjiakou | -36.5 | -4.1 | -0.9 | -10.8 | NA | NA | NA | NA | NA | NA | NA | NA |
| Zhengzhou | -43.3 | -50.1 | -1.9 | -5.9 | -45.5 | -54.7 | -1.9 | -6.1 | -52.1 | -40.3 | -1.4 | -0.1 |
| Zhoukou | -34.9 | -32.1 | -0.8 | -8.9 | -44.9 | -48.8 | -1.7 | -6.3 | -70.2 | -63.5 | -0.7 | -9.6 |
| Zhumadian | -28.9 | -37.0 | -0.3 | -6.9 | -37.4 | -58.5 | -1.5 | -4.9 | NA | NA | NA | NA |
| Zibo | -56.6 | -90.4 | -9.2 | -19.1 | -43.5 | -62.4 | -6.2 | -11.7 | -39.0 | -66.5 | -4.8 | -12.3 |
| Xingtai | NA | NA | NA | NA | -54.9 | -74.6 | -5.1 | -10.7 | -68.0 | -99.1 | -6.0 | -13.0 |

Table S9. Mean impact of pollution alerts on urban air quality (model trained using data from air quality monitoring stations). Values represent the mean (± standard error) differences between observed pollutant concentrations and the model-predicted counterfactual concentrations for the same period assuming no alerts. Negative values therefore signify that air quality improved because of the alerts, whereas positive values indicate a deterioration or no net benefit at the median point.

| **City** | **PM_2.5_ (μg m^-3^)** | | | **PM_10_ (μg m^-3^)** | | | **NO_2_ (μg m^-3^)** | | | **SO_2_ (μg m^-3^)** | | |
| --- | --- | --- | --- | --- | --- | --- | --- | --- | --- | --- | --- | --- |
|  | **Yellow** | **Orange** | **Red** | **Yellow** | **Orange** | **Red** | **Yellow** | **Orange** | **Red** | **Yellow** | **Orange** | **Red** |
| Anyang | -74.6±8.6 | -56.7±2.7 | -57.5±5.3 | -36.7±8.4 | -57.7±3.2 | -42.1±6.2 | 2.0±2.1 | -8.7±0.6 | -4.6±1.2 | 3.5±1.4 | -0.7±0.3 | 0.9±0.6 |
| Baoding | -36.9±6.3 | -50.3±2.3 | -51.2±4.3 | -65.7±9.4 | -92.9±3.0 | -102.5±7.2 | -11.9±2.5 | -18.3±0.7 | -10.3±2.2 | -3.3±0.9 | -4.4±0.2 | -0.6±0.8 |
| Beijing | -18.7±4.4 | -29.5±6.7 | *NA* | -43.0±5.5 | -37.0±5.1 | *NA* | -11.5±1.0 | -9.1±1.9 | *NA* | -1.3±0.2 | -0.6±0.3 | *NA* |
| Binzhou | -34.0±4.3 | -34.5±2.4 | -14.1±2.8 | -48.9±5.2 | -51.8±3.3 | -40.7±4.9 | -9.4±1.7 | -12.1±0.8 | -9.3±4.2 | -8.6±0.9 | -6.3±0.5 | 0.8±5.8 |
| Cangzhou | -40.2±5.1 | -46.8±1.9 | -45.9±4.7 | -52.1±6.3 | -57.6±2.4 | -51.7±6.3 | -16.2±1.4 | -13.1±0.5 | -16.7±2.0 | -4.2±1.1 | -5.0±0.2 | -5.6±0.6 |
| Changzhi | -31.1±3.0 | -27.1±1.5 | -33.9±12.6 | -57.4±4.7 | -47.7±1.9 | -57.0±15.3 | -4.0±0.9 | -3.6±0.6 | -2.2±3.0 | -2.5±0.9 | -0.8±0.4 | -0.5±2.1 |
| Chengde | -34.1±8.0 | -35.6±7.5 | *NA* | -31.4±12.2 | -46.1±8.1 | *NA* | -6.0±3.7 | -14.6±2.9 | *NA* | -3.9±1.1 | -1.9±0.5 | *NA* |
| Datong | -34.0±4.6 | -37.0±4.3 | -18.7±5.8 | -47.9±7.2 | -52.0±5.9 | -15.4±11.0 | -3.8±1.7 | -6.1±2.0 | 9.1±3.0 | -6.5±3.6 | -7.8±1.6 | -10.0±5.0 |
| Dezhou | -66.6±12.9 | -42.6±2.9 | *NA* | -99.2±14.4 | -57.0±3.5 | *NA* | -4.6±2.9 | -9.1±0.7 | *NA* | -1.7±1.5 | -4.1±0.4 | *NA* |
| Dongying | -43.5±6.0 | -38.1±3.5 | -24.3±9.8 | -68.0±8.5 | -63.4±3.8 | -44.5±8.3 | -8.0±2.1 | -13.8±0.9 | -14.0±1.4 | -2.7±0.7 | -4.6±0.5 | 0.1±2.7 |
| Handan | -56.0±5.2 | -44.0±2.3 | -40.9±5.3 | -58.6±10.0 | -62.3±2.9 | -73.6±6.8 | -10.0±1.7 | -12.7±0.6 | -12.2±1.7 | -4.6±1.3 | -7.5±0.3 | -8.5±1.0 |
| Hebi | -78.5±8.8 | -54.8±2.3 | -63.3±4.7 | -64.0±8.7 | -63.6±2.8 | -66.3±4.8 | -5.4±1.9 | -6.8±0.7 | -10.4±1.5 | 2.5±1.1 | -1.4±0.3 | 1.0±0.6 |
| Hengshui | -83.5±12.6 | -60.0±2.4 | -42.1±3.9 | -45.2±17.5 | -66.2±2.8 | -57.2±5.8 | -8.4±3.3 | -13.2±0.6 | -1.6±2.2 | -3.1±1.3 | -3.4±0.3 | -1.4±1.2 |
| Heze | -95.0±16.9 | -46.7±2.8 | -53.3±6.7 | -90.3±8.7 | -58.4±3.2 | -63.7±6.4 | -1.7±2.5 | -7.4±0.7 | -8.5±1.3 | -1.5±1.2 | -3.6±0.3 | -3.8±0.7 |
| Jiaozuo | -54.4±7.5 | -51.6±2.7 | -48.8±6.3 | -37.1±12.3 | -47.1±3.0 | -43.0±6.3 | -1.7±1.5 | -2.3±0.7 | -1.3±1.6 | 0.2±0.7 | -1.4±0.3 | -1.5±0.5 |
| Jinan | -101.0±11.6 | -42.7±2.3 | -23.5±6.9 | -142.4±27.9 | -62.5±3.1 | -42.1±8.1 | -13.4±4.8 | -10.0±0.7 | -11.4±1.8 | -1.3±1.7 | -4.1±0.3 | -0.8±1.0 |
| Jincheng | -31.4±3.4 | -20.2±1.8 | *NA* | -56.7±4.7 | -46.8±2.6 | *NA* | -3.8±1.0 | -2.5±0.6 | *NA* | 1.0±0.8 | 2.6±0.4 | *NA* |
| Jining | -43.8±5.2 | -47.6±2.1 | -51.1±5.8 | -49.2±5.9 | -55.5±2.3 | -59.3±5.2 | -8.4±1.7 | -8.3±0.7 | -11.8±1.7 | -0.6±0.8 | -5.0±0.3 | -5.0±1.0 |
| Jinzhong | -31.5±2.1 | -26.1±1.9 | -16.0±10.8 | -48.0±3.7 | -44.8±2.8 | -30.5±14.6 | -4.2±1.0 | -4.1±0.7 | 4.6±4.4 | 1.3±1.5 | -1.3±1.0 | 1.5±5.2 |
| Kaifeng | -54.4±7.3 | -49.3±2.5 | -47.3±4.9 | -68.5±7.5 | -68.4±2.7 | -73.1±5.9 | -3.8±1.4 | -6.9±0.6 | -4.3±1.9 | -1.8±0.5 | -1.0±0.2 | -0.9±0.4 |
| Langfang | -51.2±4.3 | -48.3±2.1 | -54.6±6.8 | -82.1±5.0 | -69.7±2.8 | -67.0±11.4 | -17.3±1.3 | -14.7±0.7 | -0.6±2.7 | -3.5±0.5 | -3.4±0.2 | -1.3±0.7 |
| Liaocheng | -61.5±7.3 | -49.4±2.5 | -48.0±5.2 | -87.3±5.6 | -69.0±3.4 | -68.9±5.1 | -8.3±1.7 | -10.2±0.7 | -8.8±1.4 | -1.8±0.8 | -4.4±0.4 | -4.4±0.5 |
| Linfen | -35.0±3.1 | -52.2±2.1 | -35.2±9.9 | -43.4±4.6 | -60.6±2.4 | -5.9±11.8 | -6.0±1.0 | -8.2±0.6 | -1.1±2.1 | -0.9±1.9 | -0.7±0.8 | 22.0±5.8 |
| Linyi | -49.2±7.6 | -44.9±3.1 | -42.8±11.8 | -88.6±11.8 | -72.9±3.9 | -74.8±15.4 | -8.6±2.0 | -12.7±0.9 | -11.2±3.0 | -0.9±1.0 | -2.6±0.4 | 3.0±1.4 |
| Luohe | -36.5±5.0 | -60.4±3.3 | -75.0±10.2 | -46.7±12.7 | -67.8±3.7 | -67.3±10.7 | -5.5±1.1 | -7.3±0.6 | -4.1±1.5 | 1.6±1.5 | -1.6±0.2 | -1.5±0.5 |
| Luoyang | -41.1±4.8 | -35.0±2.6 | -43.1±6.4 | -37.7±6.2 | -29.5±3.2 | -26.7±8.2 | 2.6±1.4 | -1.3±0.7 | 1.5±1.5 | -0.3±0.4 | -0.4±0.2 | 1.0±0.5 |
| Lvliang | -15.8±3.2 | -21.5±1.0 | *NA* | -39.5±7.6 | -54.6±2.0 | *NA* | -1.2±1.6 | -2.9±0.6 | *NA* | -7.0±2.3 | -4.5±0.6 | *NA* |
| Nanyang | -87.9±8.6 | -39.6±3.3 | -59.2±6.0 | -54.8±7.5 | -46.7±3.8 | -41.4±8.3 | 3.9±1.6 | -5.5±0.7 | 0.8±1.4 | -1.0±0.5 | -0.9±0.1 | -0.6±0.4 |
| Pingdingshan | -51.6±8.1 | -46.8±3.0 | -47.8±13.1 | -54.7±11.4 | -60.6±3.3 | -36.1±16.0 | 0.6±1.9 | -4.2±0.7 | -3.1±2.6 | 0.1±0.6 | -2.9±0.2 | -1.1±0.8 |
| Puyang | -67.4±12.8 | -54.7±3.2 | -76.6±4.6 | -44.4±13.0 | -56.0±3.7 | -54.4±4.0 | 0.5±2.2 | -5.9±0.7 | -4.8±0.9 | -1.1±0.7 | -1.8±0.3 | -1.9±0.4 |
| Qingdao | -28.3±4.1 | -32.8±5.3 | *NA* | -42.4±6.5 | -48.1±7.3 | *NA* | -14.3±1.5 | -16.7±1.7 | *NA* | -1.4±0.5 | -1.4±0.6 | *NA* |
| Qinhuangdao | -21.6±8.7 | -29.4±1.8 | *NA* | -33.3±10.7 | -44.5±2.1 | *NA* | -6.3±2.5 | -7.0±0.7 | *NA* | -1.9±1.5 | -2.3±0.3 | *NA* |
| Rizhao | -31.2±3.0 | -24.6±4.5 | -12.4±8.9 | -55.1±4.7 | -48.7±5.5 | -32.2±10.3 | -7.6±1.1 | -6.0±1.4 | -3.7±2.8 | -2.1±0.4 | -1.7±0.5 | -0.7±0.9 |
| Sanmenxia | -32.4±4.7 | -32.7±2.7 | -56.7±10.2 | -38.7±6.0 | -47.6±2.9 | -32.4±12.2 | -3.4±1.2 | -3.3±0.6 | 0.1±1.9 | 0.7±0.6 | -1.2±0.2 | 0.5±0.9 |
| Shangqiu | -43.7±5.6 | -50.4±3.3 | -60.7±8.9 | -57.6±6.3 | -60.8±2.9 | -72.2±13.2 | -10.4±1.4 | -8.8±0.7 | -12.0±1.5 | -0.3±0.5 | -2.7±0.2 | -3.5±0.9 |
| Shijiazhuang | -52.9±8.4 | -63.0±2.0 | -90.6±11.7 | -61.9±10.8 | -84.7±2.9 | -126.2±11.9 | -9.2±2.8 | -15.0±0.7 | -27.1±1.7 | -1.8±0.8 | -4.0±0.2 | -11.2±1.8 |
| Shuozhou | -27.2±2.2 | -37.0±2.1 | *NA* | -88.9±11.3 | -90.8±3.7 | *NA* | -5.4±1.3 | -6.1±0.7 | *NA* | -3.4±1.6 | -7.7±1.0 | *NA* |
| Taian | -37.2±5.3 | -36.7±3.2 | -33.1±8.3 | -69.9±6.4 | -66.3±3.8 | -76.7±9.2 | -7.9±1.2 | -8.0±0.6 | -7.2±1.5 | -1.7±0.5 | -1.8±0.3 | 2.6±1.2 |
| Taiyuan | -55.7±2.9 | -54.9±3.0 | -41.0±8.5 | -71.9±4.8 | -80.0±3.9 | -55.4±10.1 | -9.3±1.1 | -9.4±0.8 | -1.6±2.6 | -5.3±1.4 | -8.7±0.8 | -9.9±2.9 |
| Tangshan | -57.5±23.3 | -55.6±1.7 | -34.1±7.5 | -67.2±30.9 | -76.2±2.1 | -73.1±9.0 | -22.7±3.8 | -16.1±0.6 | -1.5±2.6 | -10.0±3.6 | -7.2±0.4 | -8.3±2.0 |
| Tianjin | -31.9±4.7 | -31.5±6.2 | *NA* | -43.2±4.7 | -44.5±5.2 | *NA* | -10.0±1.2 | -5.9±1.8 | *NA* | -3.5±0.4 | -1.8±0.5 | *NA* |
| Weifang | -20.7±7.2 | -35.0±3.6 | -49.3±9.0 | -35.6±6.3 | -64.6±5.1 | -66.5±7.7 | -9.4±1.4 | -11.2±1.1 | -10.7±2.3 | -3.2±0.6 | -3.1±0.4 | 0.4±1.1 |
| Weihai | -18.3±3.6 | -26.4±7.5 | *NA* | -27.3±5.1 | -47.8±10.7 | *NA* | -5.2±1.0 | -3.8±1.1 | *NA* | -0.8±0.4 | -0.9±0.5 | *NA* |
| Xinxiang | -92.4±14.1 | -70.3±3.4 | -89.4±5.3 | -20.1±14.9 | -61.8±2.8 | -74.3±3.5 | 13.4±3.5 | -4.3±0.8 | -8.4±1.1 | 7.2±1.5 | -0.0±0.2 | -0.5±0.4 |
| Xinyang | -40.1±11.9 | -46.1±2.8 | -49.6±13.1 | -25.2±10.9 | -50.3±3.2 | -45.0±17.4 | -4.4±1.3 | -7.8±0.5 | -10.5±2.8 | -1.3±0.4 | -1.1±0.1 | 0.2±0.2 |
| Xinzhou | -27.9±3.0 | -26.0±1.9 | *NA* | -41.1±4.4 | -41.4±2.3 | *NA* | -7.5±1.0 | -6.6±0.6 | *NA* | -2.6±1.3 | -1.9±0.5 | *NA* |
| Xuchang | -76.7±7.9 | -57.5±3.9 | -63.6±8.8 | -54.1±6.3 | -59.0±3.2 | -53.7±8.0 | -2.9±1.7 | -8.0±0.7 | -6.2±1.5 | -0.3±0.7 | -2.5±0.3 | -1.8±0.5 |
| Yangquan | -37.4±7.6 | -44.1±2.0 | -46.1±16.0 | -37.4±9.4 | -56.1±2.6 | -51.5±20.2 | -12.9±1.8 | -10.3±0.6 | 0.2±5.0 | -4.3±1.4 | -3.0±0.7 | 27.9±6.8 |
| Yantai | -1.0±4.7 | -23.8±2.5 | *NA* | -14.3±6.8 | -39.8±3.6 | *NA* | -2.6±1.9 | -5.6±0.8 | *NA* | -1.5±0.8 | -1.8±0.3 | *NA* |
| Yuncheng | -58.5±3.5 | -60.1±2.9 | -78.9±10.4 | -73.8±4.4 | -80.6±3.4 | -103.6±10.5 | -5.6±0.9 | -9.0±0.6 | -15.6±2.0 | -2.5±0.8 | -4.5±0.4 | -10.8±2.1 |
| Zaozhuang | -14.9±8.3 | -42.6±3.0 | -52.7±12.9 | -56.0±16.7 | -65.4±3.5 | -78.2±9.9 | -5.8±2.9 | -10.3±0.8 | -16.2±2.2 | -4.4±0.9 | -4.7±0.4 | -3.9±1.4 |
| Zhangjiakou | -30.9±11.7 | *NA* | *NA* | 4.1±26.2 | *NA* | *NA* | -7.9±2.8 | *NA* | *NA* | -1.4±1.4 | *NA* | *NA* |
| Zhengzhou | -42.1±3.3 | -48.5±3.0 | -49.2±9.5 | -53.1±5.8 | -51.9±3.0 | -32.5±8.8 | -5.0±1.0 | -5.6±0.7 | 1.0±1.7 | -2.0±0.3 | -1.8±0.2 | -1.2±0.5 |
| Zhoukou | -31.0±9.4 | -51.7±4.0 | -73.8±8.5 | -38.7±7.8 | -46.4±3.4 | -65.1±6.2 | -8.3±0.8 | -5.7±0.6 | -10.5±1.5 | 0.1±0.8 | -1.8±0.2 | -0.2±0.6 |
| Zhumadian | -17.0±8.6 | -39.2±2.5 | *NA* | -24.7±10.4 | -54.2±3.0 | *NA* | -5.4±1.5 | -5.1±0.6 | *NA* | -1.1±0.4 | -1.6±0.1 | *NA* |
| Zibo | -54.3±7.0 | -43.3±2.2 | -46.4±6.6 | -83.9±11.7 | -62.3±2.9 | -63.9±7.7 | -17.6±1.7 | -11.7±0.6 | -13.2±1.5 | -9.5±1.2 | -5.9±0.4 | -5.4±1.2 |
| Xingtai | *NA* | -53.7±2.4 | -70.7±5.3 | *NA* | -66.0±2.9 | -94.8±6.5 | *NA* | -10.3±0.7 | -12.4±1.7 | *NA* | -4.9±0.3 | -5.6±0.7 |
