# Supplementary figures and images for: Significant benefits of pollution alerts for cleaner air and better health
Source: PNAS Nexus. 2026 Mar 3;5(3):pgag054. doi: 10.1093/pnasnexus/pgag054 (PMC12988777; doi:10.1093/pnasnexus/pgag054)

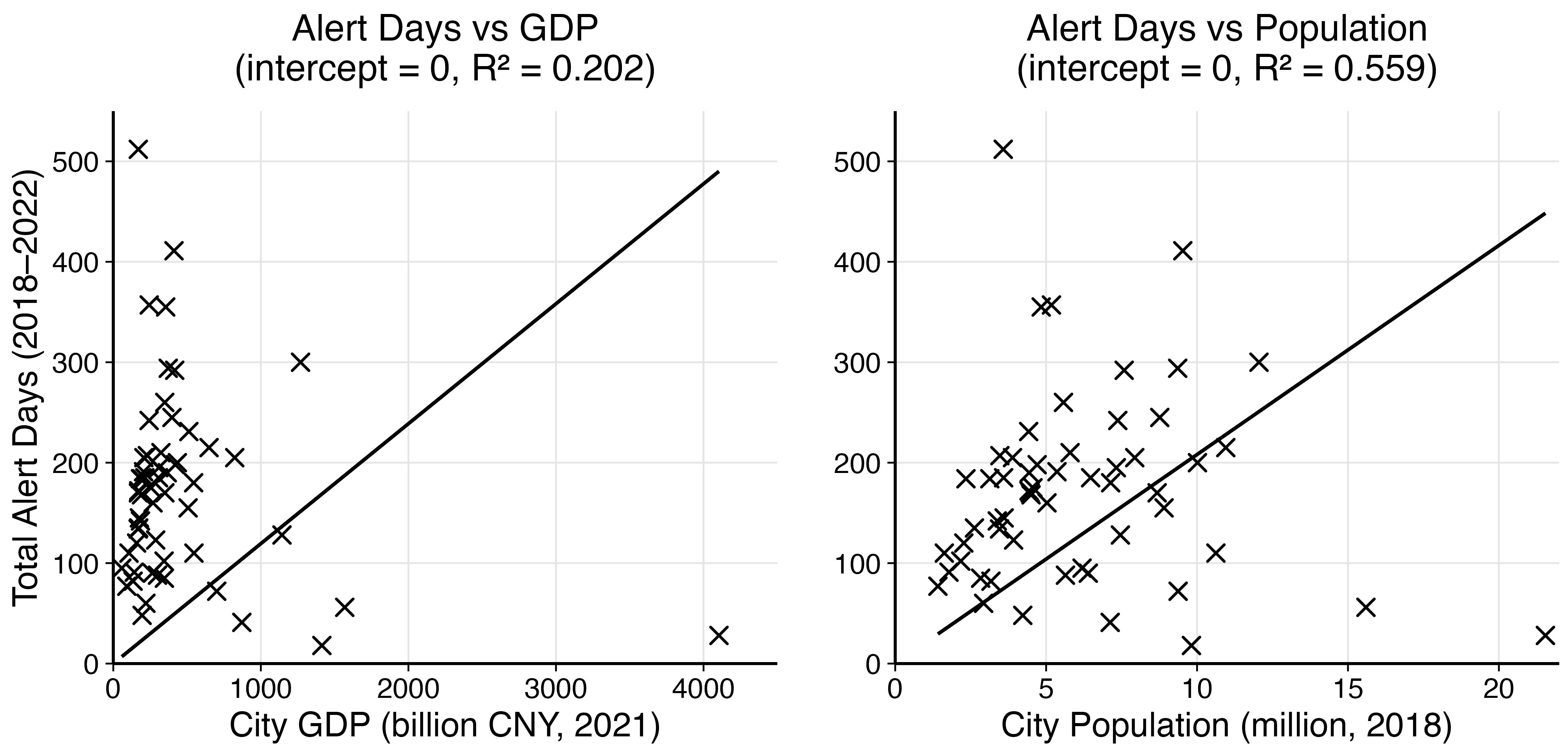

Supplement: pgag054_Supplementary_Data [file pgag054_supplementary_data.zip › PNASNEXUS-PNASNEXUS-2025-00851RR-s12.tif]

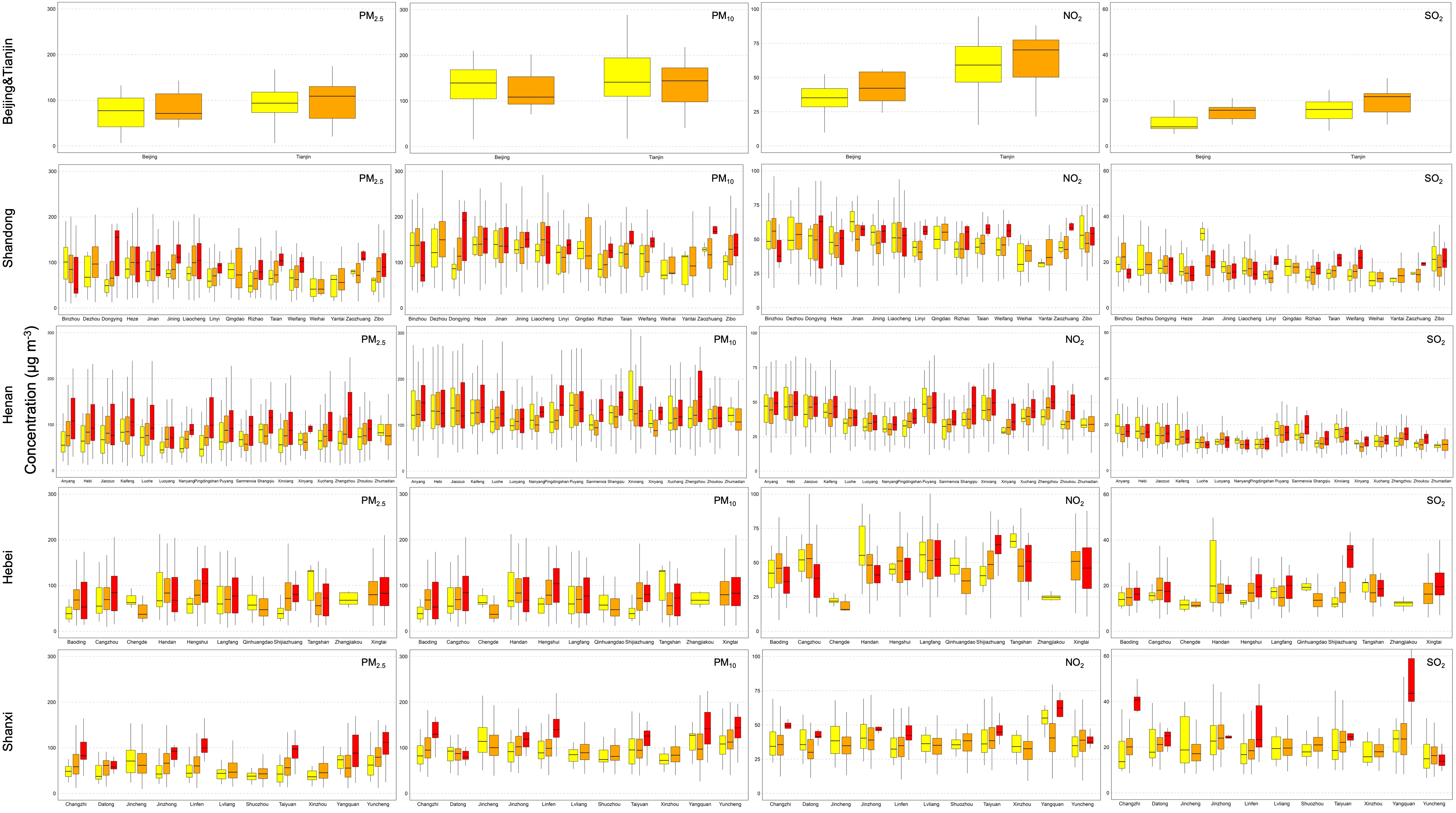

Supplement: pgag054_Supplementary_Data [file pgag054_supplementary_data.zip › PNASNEXUS-PNASNEXUS-2025-00851RR-s13.tif]

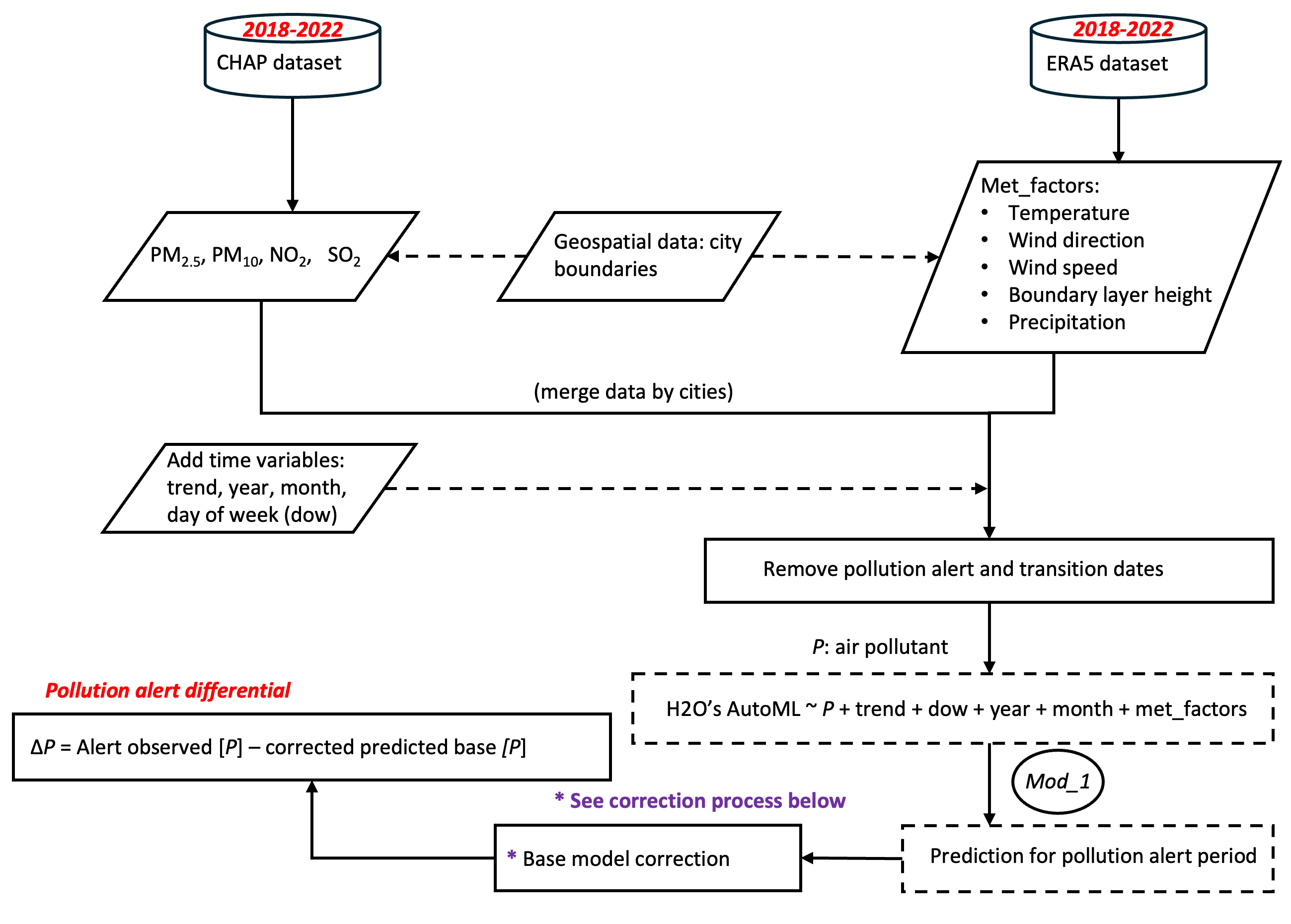

Supplement: pgag054_Supplementary_Data [file pgag054_supplementary_data.zip › PNASNEXUS-PNASNEXUS-2025-00851RR-s14.tif]

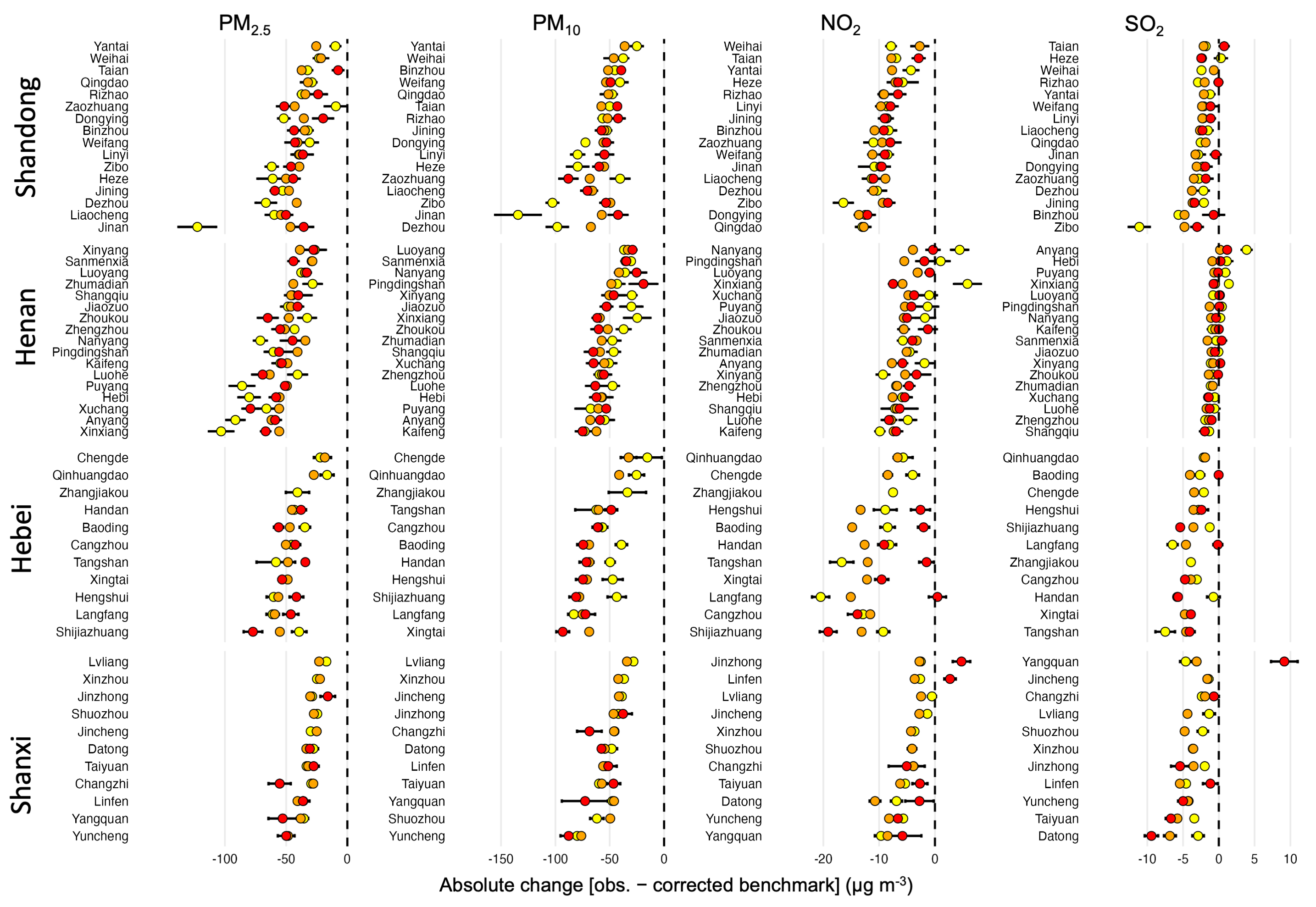

Supplement: pgag054_Supplementary_Data [file pgag054_supplementary_data.zip › PNASNEXUS-PNASNEXUS-2025-00851RR-s15.tif]

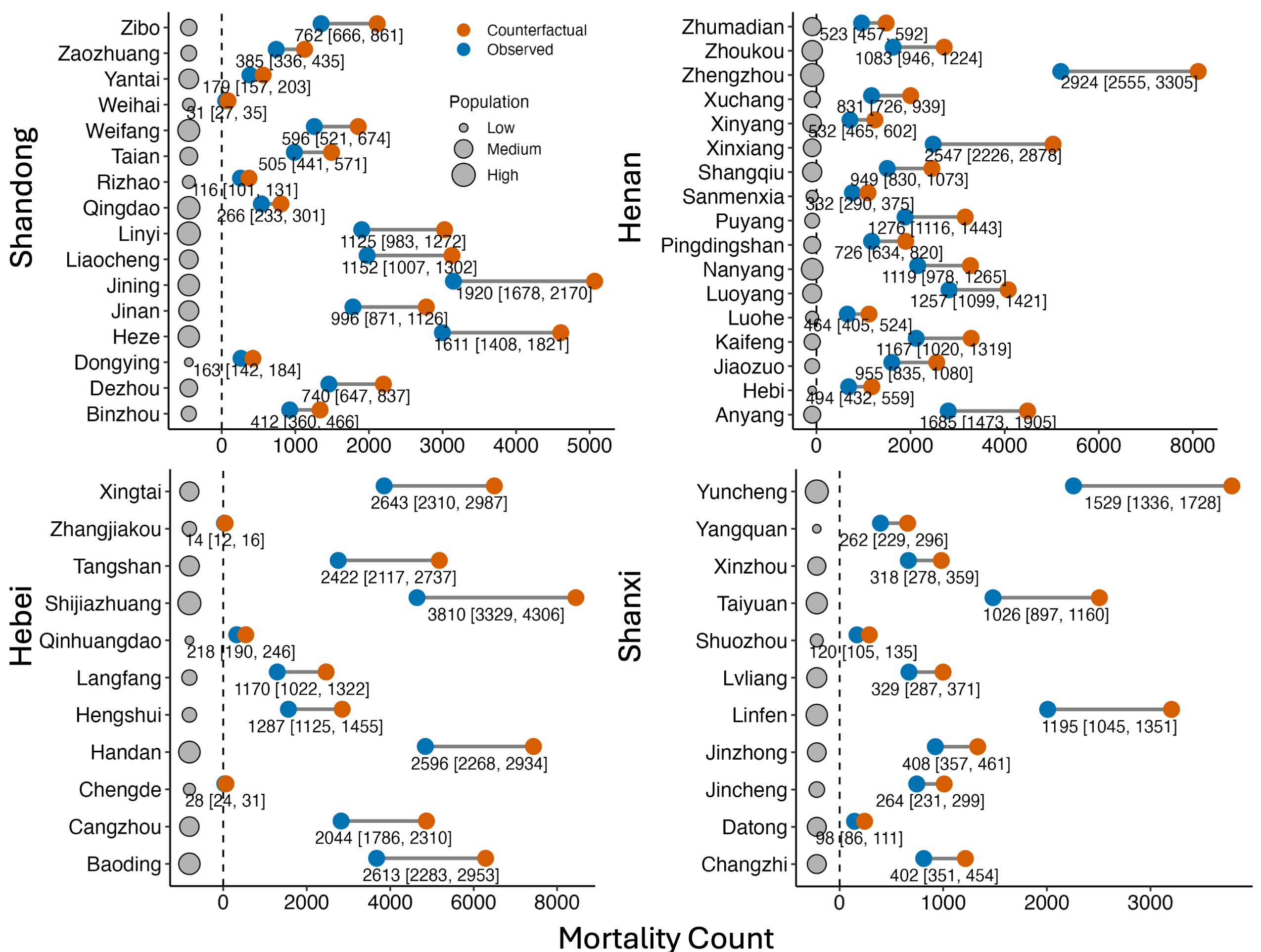

Supplement: pgag054_Supplementary_Data [file pgag054_supplementary_data.zip › PNASNEXUS-PNASNEXUS-2025-00851RR-s16.tif]

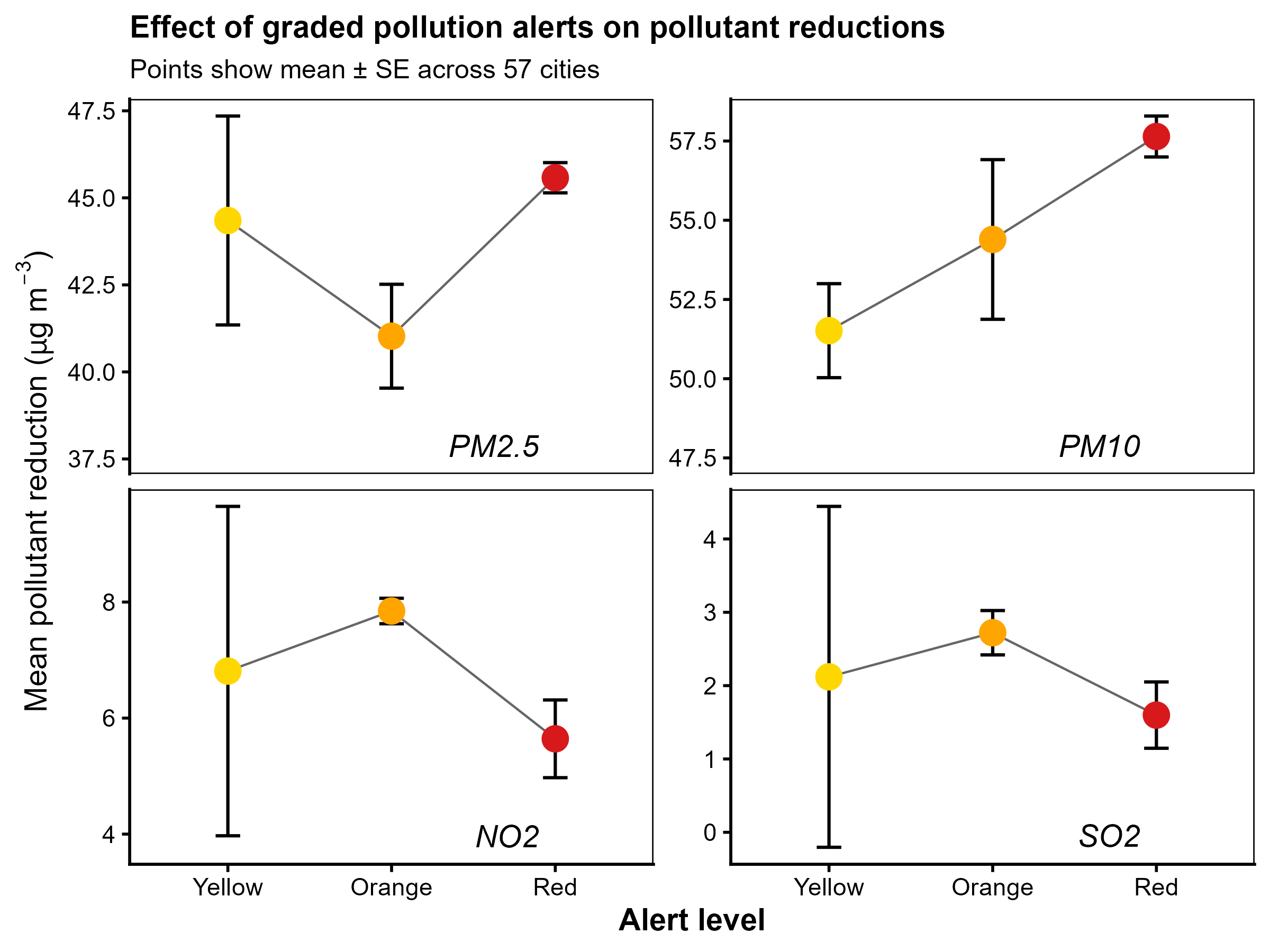

Supplement: pgag054_Supplementary_Data [file pgag054_supplementary_data.zip › PNASNEXUS-PNASNEXUS-2025-00851RR-s17.tif]

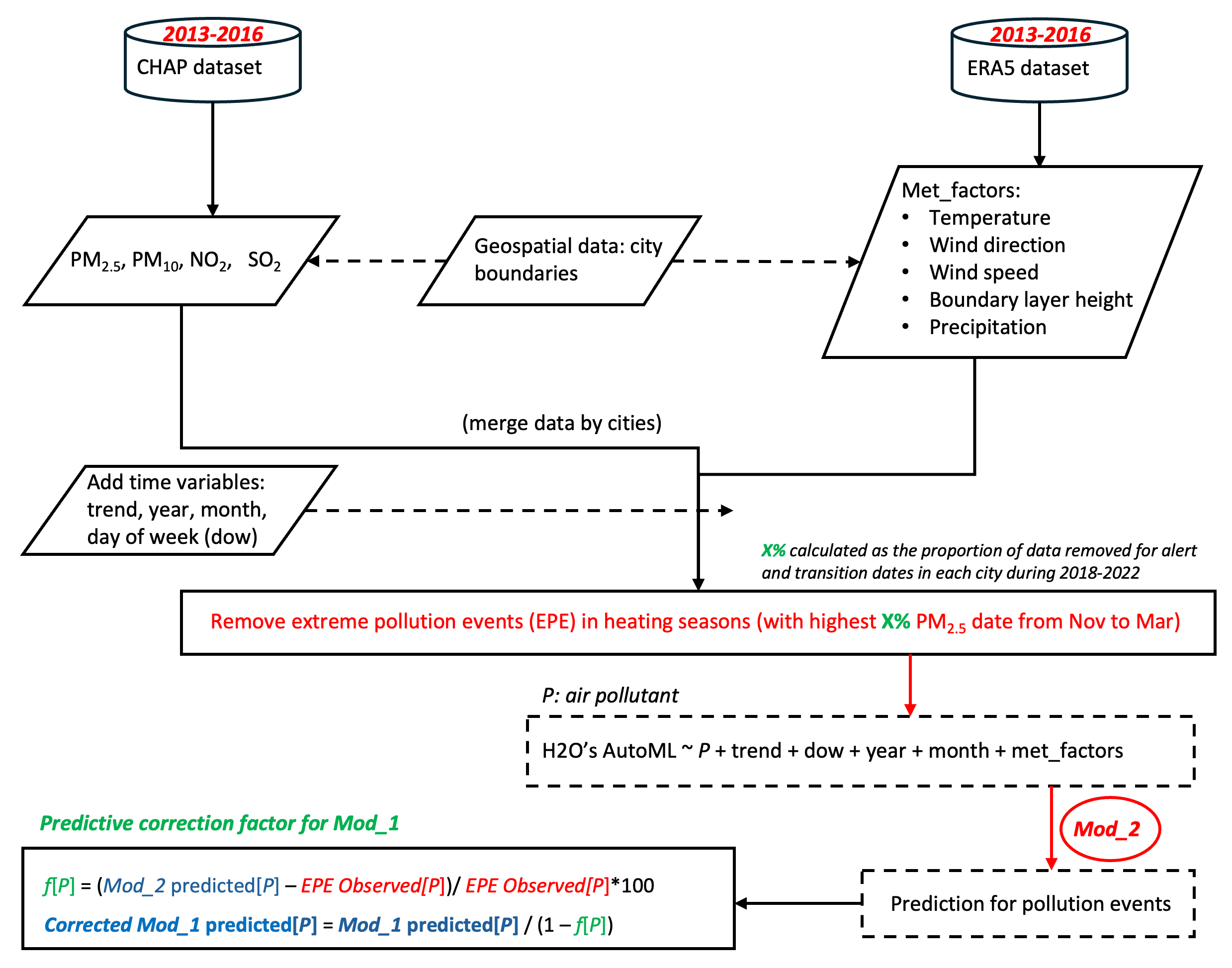

Supplement: pgag054_Supplementary_Data [file pgag054_supplementary_data.zip › PNASNEXUS-PNASNEXUS-2025-00851RR-s18.tif]

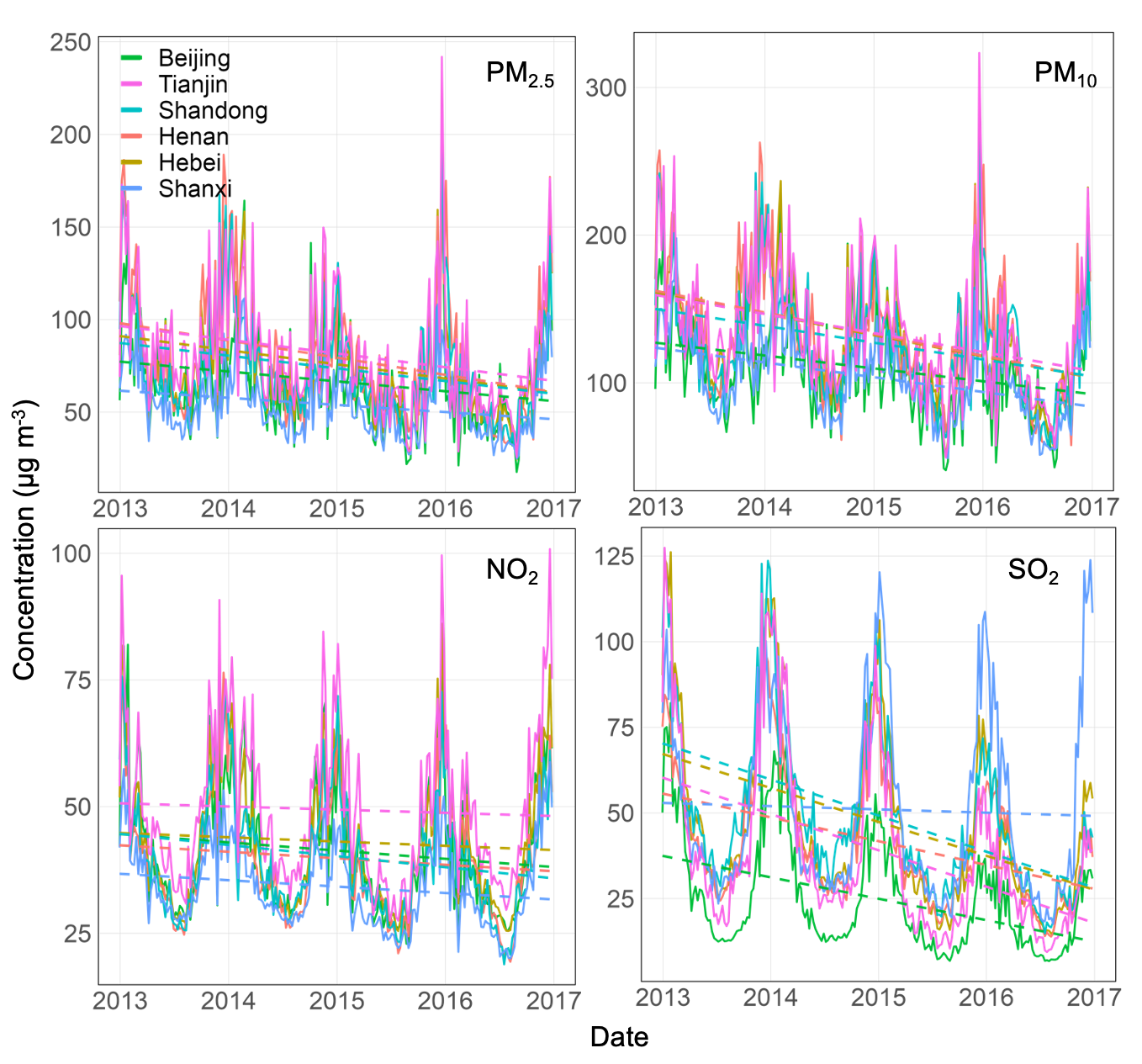

Supplement: pgag054_Supplementary_Data [file pgag054_supplementary_data.zip › PNASNEXUS-PNASNEXUS-2025-00851RR-s19.tif]

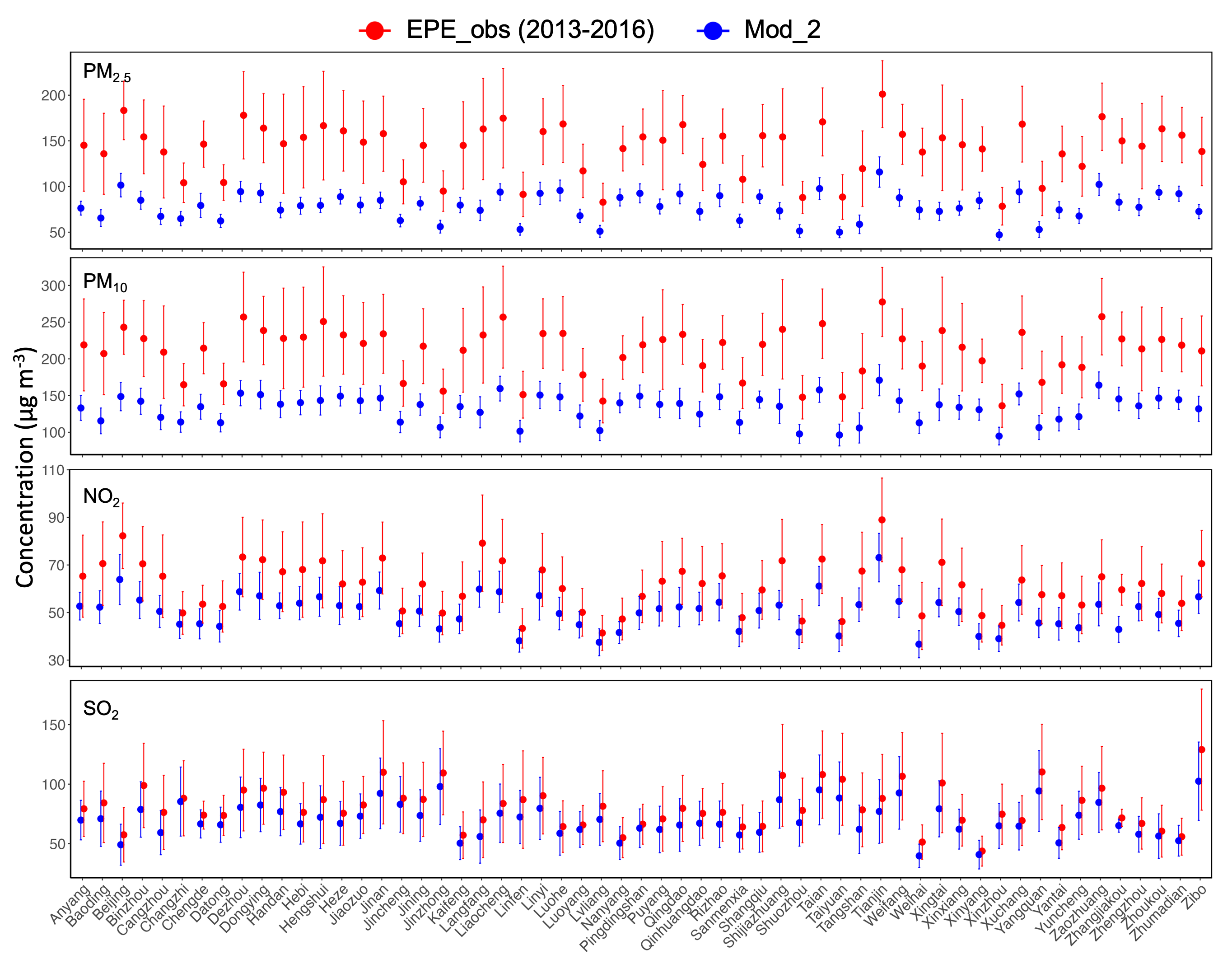

Supplement: pgag054_Supplementary_Data [file pgag054_supplementary_data.zip › PNASNEXUS-PNASNEXUS-2025-00851RR-s20.tif]

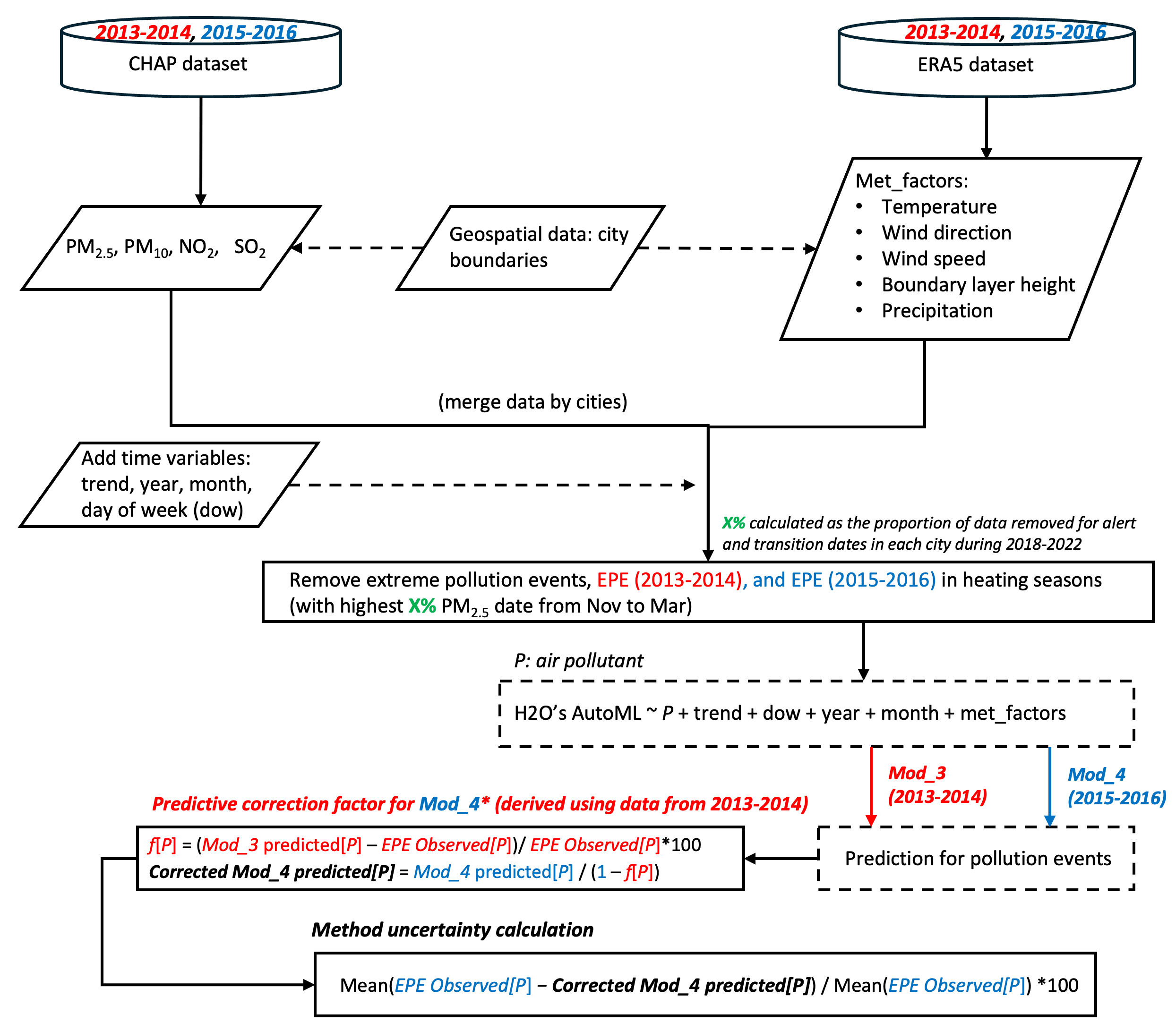

Supplement: pgag054_Supplementary_Data [file pgag054_supplementary_data.zip › PNASNEXUS-PNASNEXUS-2025-00851RR-s21.tif]

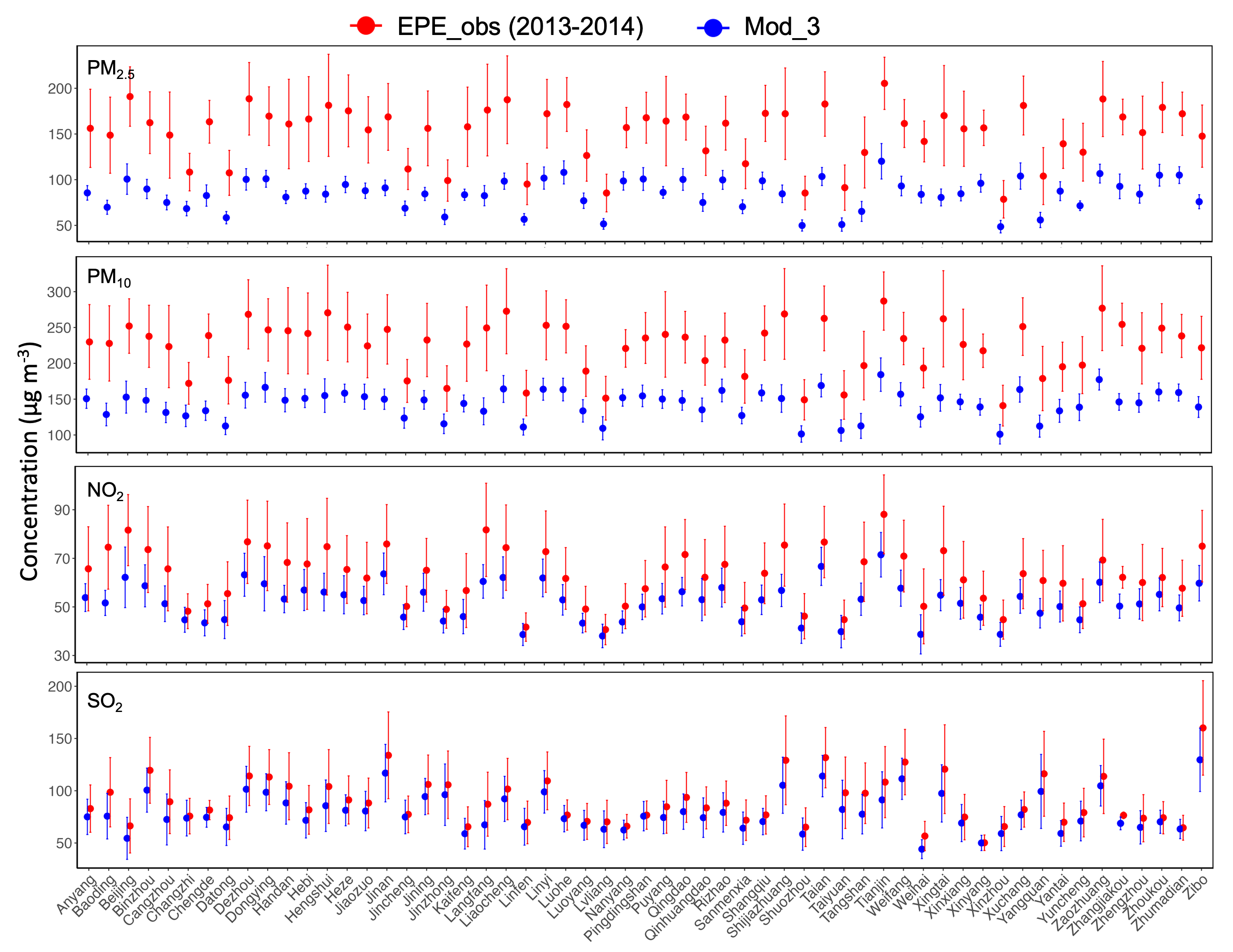

Supplement: pgag054_Supplementary_Data [file pgag054_supplementary_data.zip › PNASNEXUS-PNASNEXUS-2025-00851RR-s22.tif]

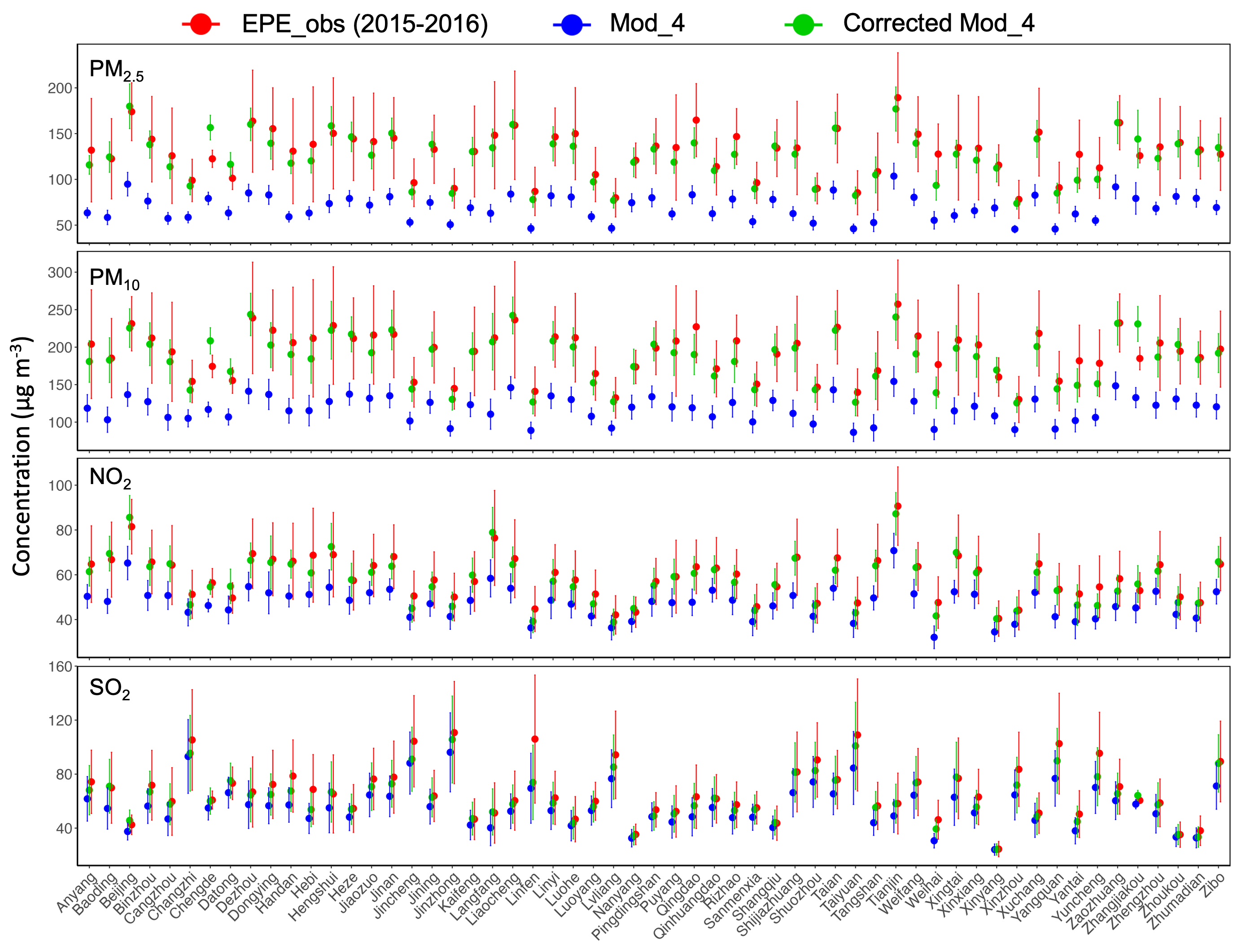

Supplement: pgag054_Supplementary_Data [file pgag054_supplementary_data.zip › PNASNEXUS-PNASNEXUS-2025-00851RR-s23.tif]

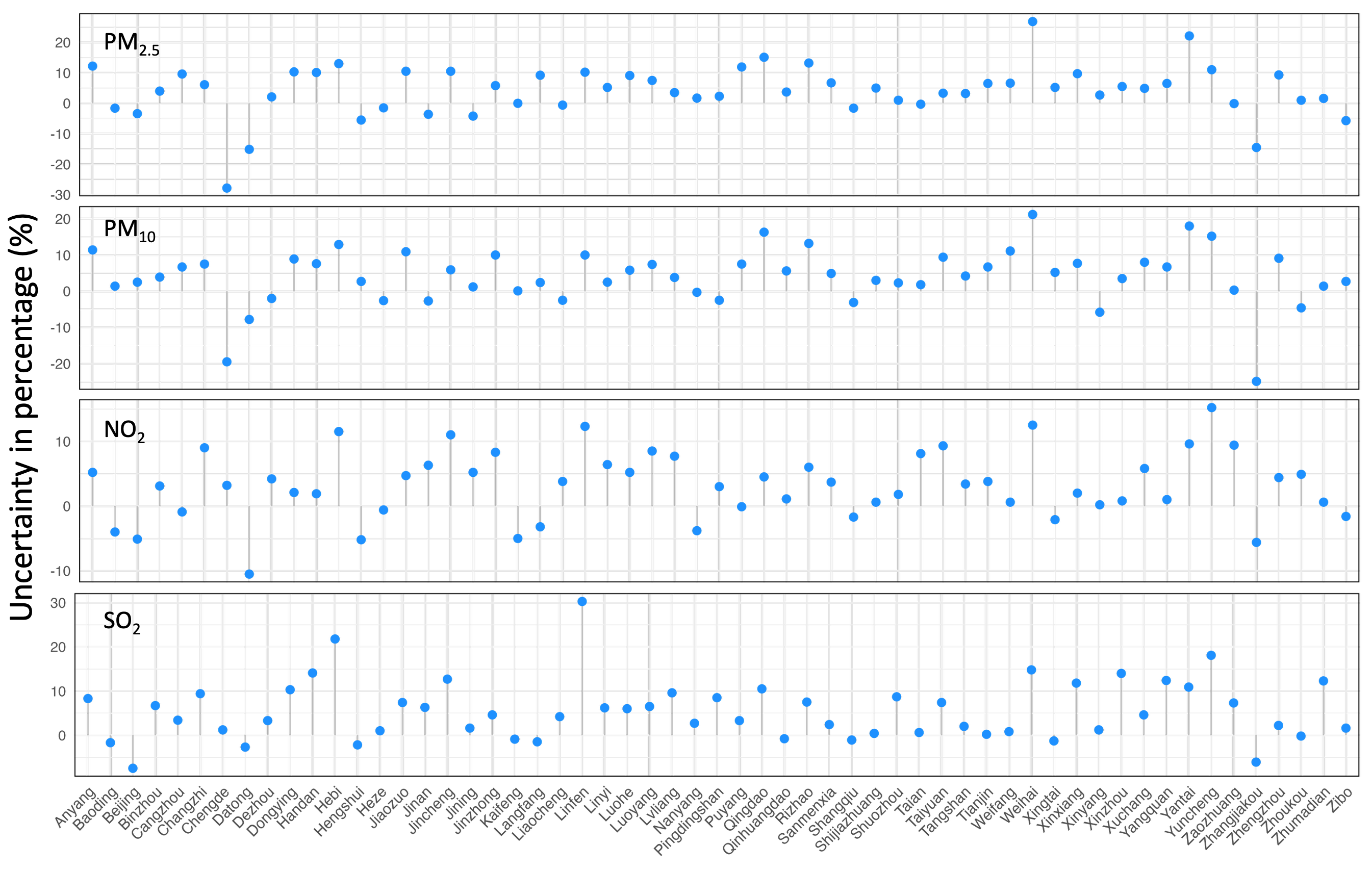

Supplement: pgag054_Supplementary_Data [file pgag054_supplementary_data.zip › PNASNEXUS-PNASNEXUS-2025-00851RR-s24.tif]

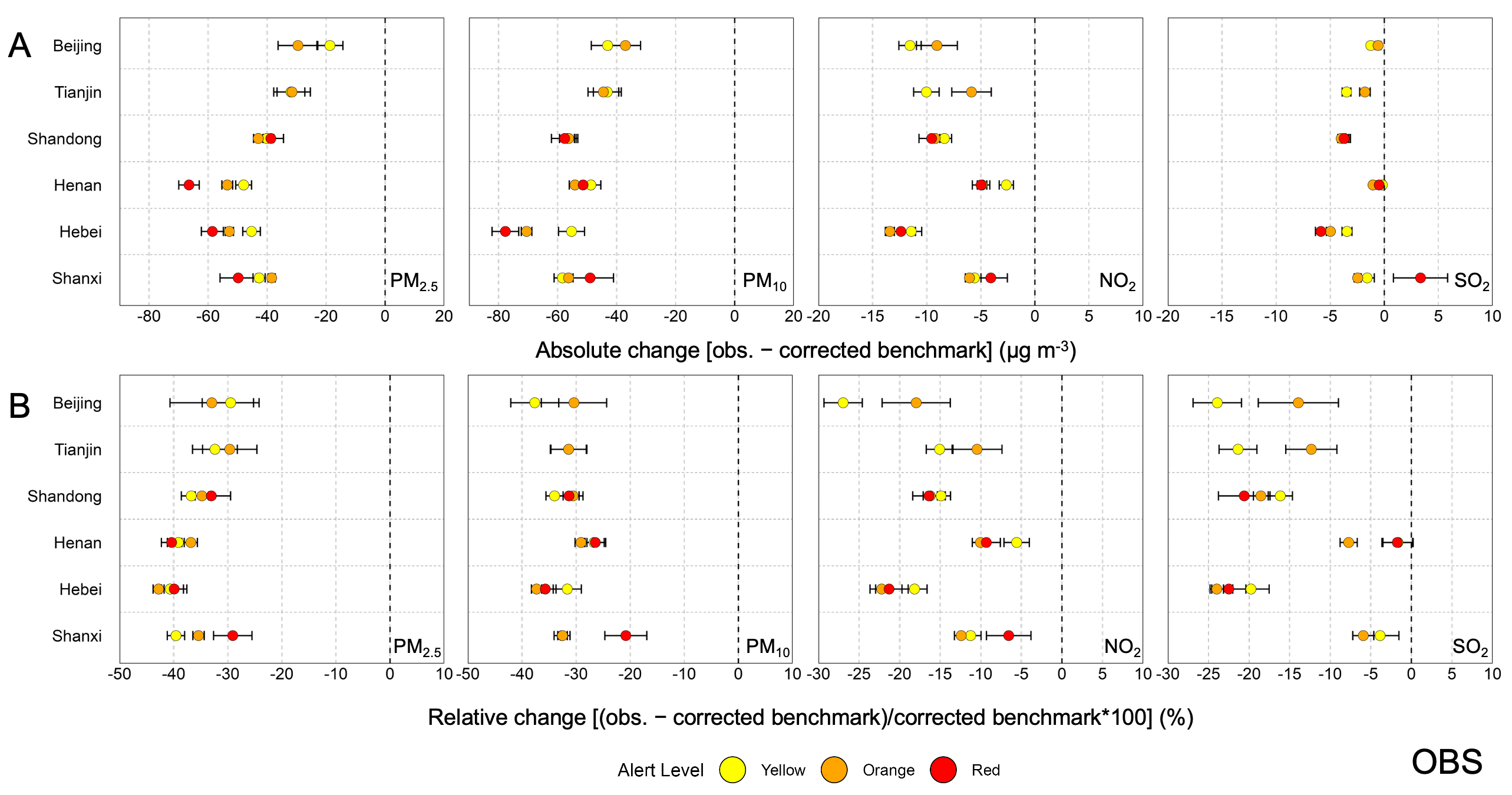

Supplement: pgag054_Supplementary_Data [file pgag054_supplementary_data.zip › PNASNEXUS-PNASNEXUS-2025-00851RR-s25.tif]

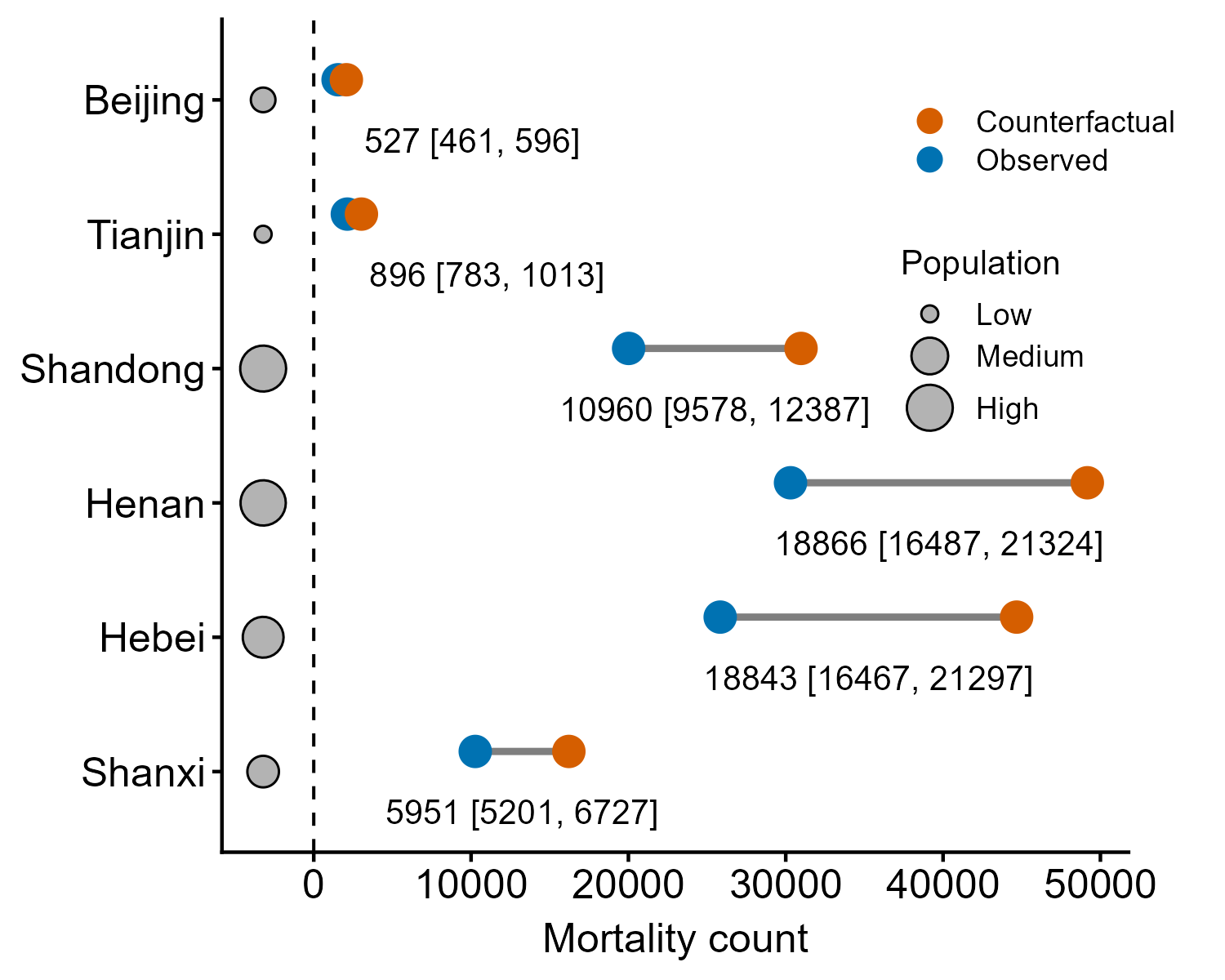

Supplement: pgag054_Supplementary_Data [file pgag054_supplementary_data.zip › PNASNEXUS-PNASNEXUS-2025-00851RR-s26.tif]

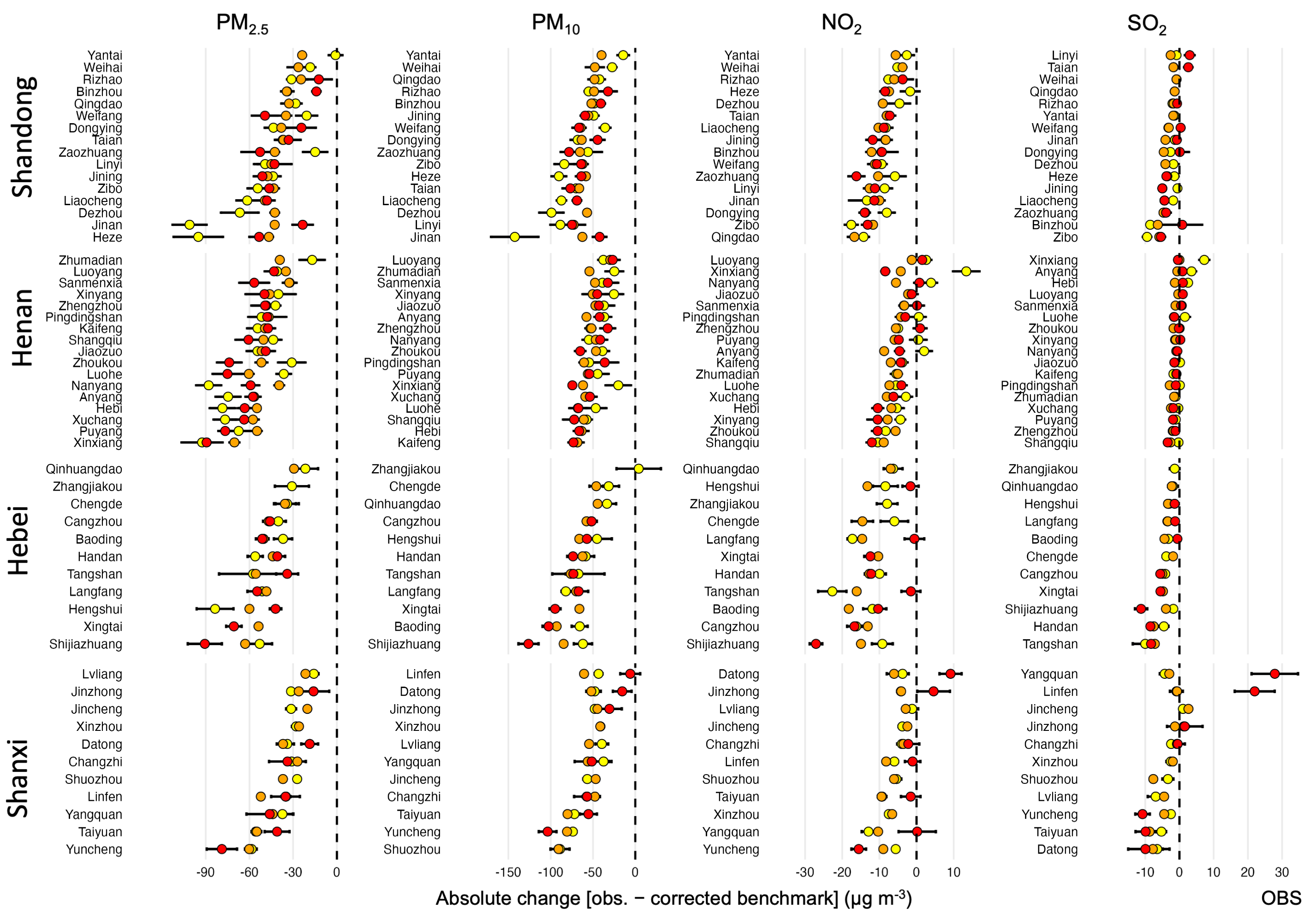

Supplement: pgag054_Supplementary_Data [file pgag054_supplementary_data.zip › PNASNEXUS-PNASNEXUS-2025-00851RR-s27.tif]
